# The Parkinson's Disease Drug Tolcapone and Analogues are Potent Glycomimetic Lectin Inhibitors of *Pseudomonas aeruginosa* LecA

**DOI:** 10.1002/anie.202508864

**Published:** 2025-11-02

**Authors:** Steffen Leusmann, Eike Siebs, Sakonwan Kuhaudomlarp, Annabelle Varrot, Anne Imberty, Bernd Kuhn, Christian Lerner, Uwe Grether, Alexander Titz

**Affiliations:** ^1^ Chemical Biology of Carbohydrates (CBCH) Helmholtz‐Institute for Pharmaceutical Research Saarland (HIPS), Helmholtz Centre for Infection Research 66123 Saarbrücken Germany; ^2^ Department of Chemistry PharmaScienceHub (PSH), Saarland University 66123 Saarbrücken Germany; ^3^ Deutsches Zentrum für Infektionsforschung (DZIF), Standort Hannover‐Braunschweig Germany; ^4^ Univ. Grenoble Alpes, Centre National de la Recherche Scientifique (CNRS), Centre de Recherches sur les Macromolécules Végétales (CERMAV) Grenoble 38000 France; ^5^ Department of Biochemistry Faculty of Science Mahidol University Bangkok 10400 Thailand; ^6^ Pharmaceutical Research and Early Development (pRED), Roche Innovation Center Basel F. Hoffmann‐La Roche AG Basel 4070 Switzerland

**Keywords:** Catechols, Drug repurposing, Glycomimetics, Inhibitors, Lectins

## Abstract

The notorious pathogen *Pseudomonas aeruginosa* relies on the lectin LecA for host cell adhesion, invasion, and biofilm formation. Motivated by the pressing need for new anti‐infective therapies caused by antimicrobial resistance, inhibitors of LecA are under investigation. Complementary to the use of carbohydrate‐based inhibitors, we have previously identified catechols as weak but specific ligands of LecA, constituting a novel class of non‐carbohydrate glycomimetics. By growing the initial millimolar fragment hits, we identified Tolcapone as a promising compound. To gain insight into the structure‐activity relationship (SAR) of catechols as LecA binders, more than 3,200 compounds of the Roche in‐house library were experimentally screened in a competitive binding assay at three concentrations. Of these, 48 compounds were chosen for further investigation, resulting in compounds equipotent to aryl galactosides, the current epitome of LecA inhibition. X‐ray crystallography and saturation transfer difference (STD) NMR spectroscopy revealed conserved interactions of the catechol moiety in the glycan binding site of LecA and rationalized the observed SAR. Our findings demonstrate that it is possible to develop potent non‐carbohydrate glycomimetic lectin inhibitors. This work paves the way for a new avenue of research towards innovative anti‐infective drugs. In a more general perspective, such small molecules also hold potential to challenge the hegemony of antibodies for lectin inhibition in clinical use.

## Graphical abstract

We present Tolcapone and derivatives as a new class of potent glycomimetics for lectin inhibition. Over 3200 Roche in‐house compounds were screened experimentally and a subset was biophysically evaluated. Our findings show the potential to develop potent non‐carbohydrate glycomimetic lectin inhibitors, opening a path toward innovative anti‐infective drug research.

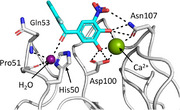

## Introduction

The antibiotic resistance crisis necessitates the development of new drugs and therapy regimes to fight the increasing threat of bacterial infections.^[^ ^1^ ^]^ In 2019, antimicrobial resistance was associated with 5 million deaths globally.^[^ ^2^ ^]^ This number is projected to increase to over 8 million deaths per year by 2050, coinciding with an annual loss of > 46 million life years due to antimicrobial resistance.^[^ ^3^ ^]^ To guide the development of novel antimicrobial drugs the World Health Organization classified carbapenem‐resistant *P. aeruginosa* as a pathogen of high concern.^[^ ^4^ ^]^ The opportunistic pathogen *P. aeruginosa* especially threatens predisposed individuals, e.g., patients in intensive care units or those suffering from cystic fibrosis. Therapy of the resulting lung, wound, urinary tract, or bloodstream infections is impeded by high rates of intrinsic and acquired antibiotic resistances.^[^ ^5^ ^]^

One innovative approach attracting growing attention is the development of antivirulence drugs, also called pathoblockers.^[^ ^6^ ^]^ In contrast to antibiotics, these molecules do not affect bacterial survival fitness but merely aim at disarming the pathogens which may also reduce the risk for resistance development. The inhibition of bacterial adhesion, host cell invasion and biofilm formation, key mechanisms of infection and chronicity, is a prime target of the antivirulence approach.

During the infection cycle *P. aeruginosa* utilizes one of its lectins, LecA, to bind to the mammalian glycolipid globotriaosylceramide (Gb3, CD77) to facilitate host cell adhesion and subsequent invasion.^[^ ^7^ ^]^ In addition to host cell invasion, LecA is of crucial importance for biofilm formation as evidenced by deletion experiments.^[^ ^8^ ^]^ Biofilms are a mechanism of adaptive resistance and act as diffusion barrier for antibacterial agents and the immune system.^[^ ^5^ ^]^ At the same time, the biofilm also reduces access to nutrients resulting in metabolically less active bacteria, which are less prone to the activity of most antibiotics that generally target active bacterial metabolism.^[^ ^9^ ^]^ Biofilms consist of bacteria embedded in a complex hydrogel of extracellular nucleic acids, proteins, lipids and exopolysaccharides.^[^ ^10^ ^]^ Soluble lectins such as LecA provide rigidity to the biofilm by crosslinking glycans of bacteria, host cells, and matrix components.^[^ ^11^ ^]^

The significant role of LecA in pathogenicity and the potential of LecA inhibition was demonstrated in murine in vivo infection experiments.^[^ ^12^, ^13^ ^]^ The administration of LecA inhibitors not only reduced lung damage and inflammation as well as bacterial dissemination but also showed synergistic effects with common antibiotics. Notably, these findings aligned closely with clinical observations in patients suffering from severe *P. aeruginosa* lung infections. Here, the inhalation of carbohydrate‐containing aerosols decreased bacterial colonization and infection severity.^[^ ^14^, ^15^ ^]^ Consequently, targeting LecA is considered a promising strategy to break antimicrobial resistance in *Pseudomonas* infections.^[^ ^11^, ^16^ ^]^

LecA is a homotetrameric lectin with affinity for d‐galactosides.^[^ ^17^, ^18^, ^19^ ^]^ Importantly, binding to LecA is highly dependent on the complexation of a single calcium–ion present in the carbohydrate binding site. The design of LecA inhibitors focused on the derivatization of d‐galactose present in its natural carbohydrate ligands such as Gb3. Addition of a β‐linked aromatic aglycon lowered the *K*_D_ value from 87 µM for d‐galactose to 14 µM in *para‐*nitrophenyl β‐d‐galactoside (*p*NPGal).^[^ ^20^ ^]^ Recently, Bruneau et al. reported on a benzophenone modified galactoside reaching an affinity of 1 µM.^[^ ^21^ ^]^ The improved potency of such aryl β‐d‐galactosides has been attributed to a CH–π interaction with His50 in close proximity to the carbohydrate‐binding site.^[^ ^20^ ^]^ Furthermore, the multivalent presentation of binding epitopes is a common strategy to improve the affinity of lectin ligands.^[^ ^22^ ^]^ While nanomolar affinities can be reached, the large size of multivalent glycosides prevents oral bioavailability and could lead to immunogenicity. Furthermore, in the case of LecA inhibitors, multivalent binding could lead to an undesired increase in biofilm cohesion and bacterial aggregation by crosslinking LecA tetramers with bacteria and host cells. Additionally, selectivity is a concern due to the presence of numerous host lectins recognizing the same carbohydrate epitope, e.g., the galectin family.^[^ ^16^ ^]^ A notable exception to large multivalent ligands are divalent precision ligands bridging the two neighboring sites in one homotetramer of LecA,^[^ ^23^, ^24^, ^25^, ^26^ ^]^ which results in nanomolar binders with promising antivirulence efficacy in diverse functional in vitro assays.^[^ ^27^, ^28^, ^29^ ^]^

Following a different rationale, we recently reported on catechols as orthosteric, non‐carbohydrate binders of calcium‐dependent lectins such as LecA.^[^ ^30^ ^]^ Catechols are generally considered to be pan‐assay interference compounds (PAINS).^[^ ^31^ ^]^ Nevertheless, a vigorous cascade of screening and hit validation confirmed electron‐deficient catechols as LecA binders with a clear binding mode without unspecific reactivity. Due to their more druglike characteristics, molecules of this compound class likely possess favorable pharmacokinetic properties. Here, we report on our efforts to increase the affinity of the known weakly binding catechol‐containing fragments towards LecA in order to establish a new class of potent glycomimetics.

## Results and Discussion

### Identification and Evaluation of Tolcapone as LecA Inhibitor

**Figure 1:**
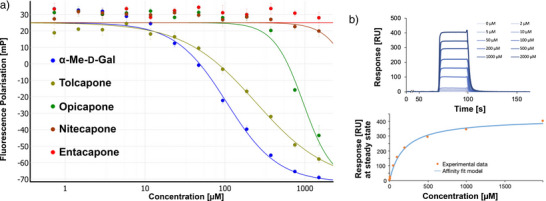
a) The inhibition of LecA by COMT inhibitors was analyzed in a competitive binding assay based on fluorescence polarization (one representative dataset shown, error bars from technical triplicates). b) SPR sensorgram and steady‐state analysis of binding of Tolcapone to LecA (one representative example is shown, replicates are depicted in the Supporting Information).

In our previous work^[^ ^30^ ^]^ on catechols as novel LecA binders two observations caught our attention: first, the affinity to LecA seemed to improve with increasing electron‐deficiency of the catechol as evidenced by cyano and nitro derivatives; second, a benzophenone motif greatly benefitted the binding affinity. Therefore, we focused our attention on commercially available nitro‐catechols. Prominent derivatives are inhibitors of the enzyme catechol‐*O*‐methyltransferase (COMT) and approved drugs for the treatment of Parkinson's disease. Four commercial COMT inhibitors were thus evaluated in a previously reported competitive binding assay based on fluorescence polarisation (FP, Table 1, Figure 1).^[^ ^32^ ^]^

**Table 1:** Approved COMT inhibitors and the investigational drug Nitecapone were evaluated for their LecA inhibition in a competitive binding assay up to 1.5 mM. Averages and standard deviation from *n* ≥ 2 experiments are given.

ID	Name	Structure	IC_50_ [µM]
1	Nitecapone	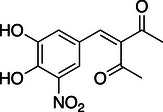	no inhibition
2	Entacapone	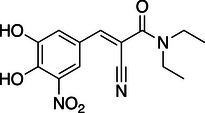	no inhibition
3	Opicapone	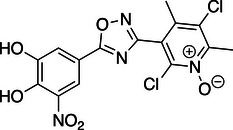	842 ± 134
4	Tolcapone	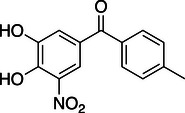	257 ± 40

While Nitecapone and Entacapone carry acyclic vinyl residues, Opicapone and Tolcapone are substituted with (hetero‐)aromatic systems. Importantly, Tolcapone combines the strongly electron‐withdrawing nitro group with a benzophenone motif. Interestingly, structurally similar Nitecapone and Entacapone were inactive at the highest tested concentration of 1.5 mM despite carrying the nitro‐catechol pharmacophore. Opicapone showed an IC_50_ value of 842 ± 134 µM, which is a significant improvement compared to the initial millimolar screening hits. Finally, Tolcapone showed the highest inhibition of LecA with an IC_50_ value of 257 ± 40 µM.

To confirm the affinity of Tolcapone toward LecA in an orthogonal assay, the compound was then evaluated in a direct binding experiment by surface plasmon resonance (SPR). A *K*_d_ of 181 ± 6 µM was determined, which is in good agreement with the IC_50_ of 257 ± 40 µM from the FP assay (Figure 1). Tolcapone significantly surpasses the affinity of our previous hits 4‐nitrocatechol (*K*_D_  =  560 ± 340 µM) and 3,4‐dihydroxybenzophenone (*K*_D_  =  3460 ± 410 µM),^[^ ^30^ ^]^ which underlines the hypothesis of beneficial contributions of a nitro group and the benzophenone motif.

### Binding Mode of Tolcapone in Complex with LecA by X‐ray Crystallography and STD NMR

**Figure 2:**
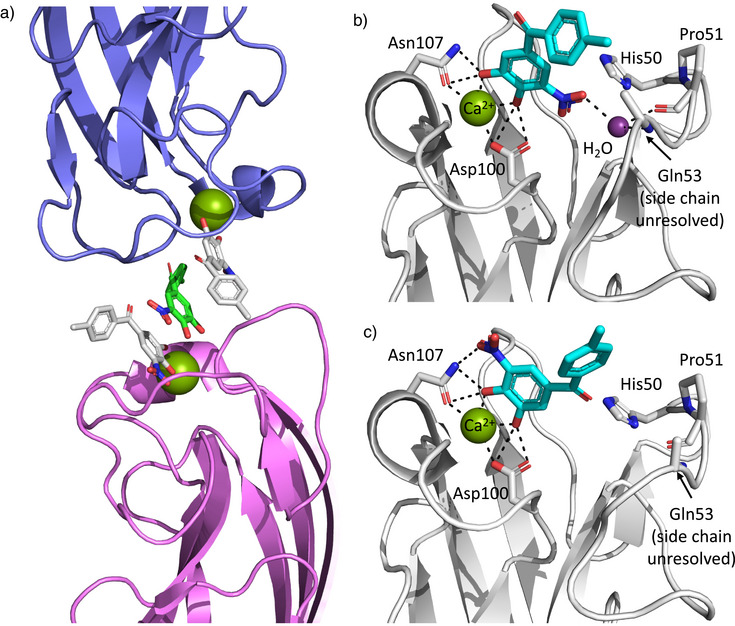
The crystal structure of LecA complexed with Tolcapone (pdb code 8GUV). a) Two different binding modes of Tolcapone were observed for carbohydrate binding sites of neighboring asymmetric units (pink and blue). A third Tolcapone molecule is stacked between the two protein‐bound ligands (green). b) Binding pose I: the nitro group engages in water‐mediated hydrogen bonding to the protein backbone and the ketone points away from the protein. c) Binding pose II: the nitro group is in hydrogen bonding distance with Asn107 and the carbonyl group points toward the protein. Dashed lines indicated hydrogen bonding distance or metal complexation.

To rationalize the high affinity of Tolcapone to LecA we conducted X‐ray crystallography studies. Crystals of LecA in complex with Tolcapone were obtained and solved at a resolution of 1.32 Å (pdb code 8GUV). In this structure, electron density could be unambiguously assigned to Tolcapone present in the carbohydrate binding sites of LecA. Interestingly, crystal contacts between protomers of neighboring asymmetric units led to two different binding poses of Tolcapone (Figure 2). In addition, a third Tolcapone molecule is stacked between the two protein–bound ligands (Figure 2a). A comparison with the co‐crystal structure of LecA and 3‐cyanocatechol (pdb code 6YO3) revealed that the catechol motif is always bound in a conserved manner with the phenyl ring and hydroxy groups occupying the same space independent of the binding pose of Tolcapone (Figure S2A). Importantly, in both LecA‐bound poses Tolcapone mimics the key calcium complexation and hydrogen bonding interactions to Asp100 and Asn107 established by OH3 and OH4 of the native ligand galactose (Figure S2B).^[^ ^19^ ^]^ Additionally, the backbone carbonyl and side chain of Thr104 as well as the backbone carbonyl of Tyr36 are located within hydrogen bonding distance (< 3.5 Å) of the catechol. With a p*K*_a_ value of ∼ 4.6 of one of the hydroxy groups, Tolcapone is likely deprotonated in these interactions. However, a conclusion on the protonation state in the crystal cannot be drawn.

Notably, Tolcapone is horizontally flipped by 180° between the two observed poses. While the ketone is exposed to the solvent in one orientation (Figure 2b), it points to the protein surface in the other (Figure 2c).

In binding pose I, Tolcapone's nitro group extends into a pocket usually occupied by OH6 of galactose where the same water‐mediated interaction to the protein backbone of Gln53 and Pro51 is formed (Figure 2b). The same interaction was observed in the complex of LecA with 3‐cyanocatechol (pdb code 6YO3).^[^ ^30^ ^]^ While OH6 of galactose engages in two more hydrogen bonds with His50 and Gln53, these interactions were not observed for the nitro group of Tolcapone, in part because the side chain of Gln53 was not resolved. The carbonyl group of Tolcapone points away from the protein toward the solvent with the *p*‐tolyl ring likely engaging in weak hydrophobic interactions with the protein surface. The CH–π interaction with His50, which is the rationale for the high affinity of aryl galactosides is absent (distance ∼7 Å).

In contrast, the carbonyl group of Tolcapone points toward the protein in binding pose II (Figure 2c). Notably, the water molecule allowing the indirect hydrogen bonding to the protein backbone from pose I was not observed in this orientation. However, a superposition of both poses of Tolcapone suggests the ketone is in a suitable position to form this interaction in case the water molecule is present (Figure S2A, cf. Figure 6). Additionally, the ketone is located within 3.3 Å of the Nτ of His50. Due to an intramolecular hydrogen bond of His50 Nπ to the main chain carbonyl of Pro51, a protonated state of His50 is required for an additional interaction with Tolcapone. The flipped orientation furthermore positions the nitro group in hydrogen bonding distance to Asn107. The pocket of LecA that is usually occupied by galactose‐OH6 remains unoccupied. A rigid body movement of the loop around Gln53 is observed (up to 2.4 Å for Gln53‐C⍺), potentially caused by a steric effect of the tolyl residue or the crystal artifact of the third Tolcapone molecule. An overlay of the binding poses highlighting these changes is shown in Figure S3.

**Figure 3:**
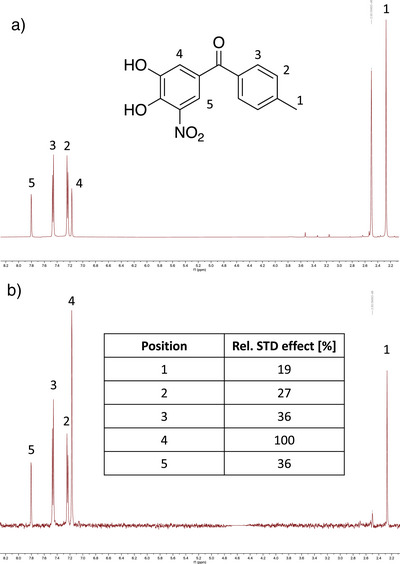
Saturation transfer difference (STD) NMR spectroscopy of Tolcapone in presence of LecA. a) Off resonance spectrum without protein saturation. b) Resulting STD NMR spectrum and calculated STD effect for protons of the ligand. The peak at 2.5 ppm corresponds to residual DMSO.

In the solid state, two different binding poses for Tolcapone were observed. To shed light on the situation in solution, we conducted STD NMR spectroscopy with LecA (Figure 3). Assignment of the resonances of Tolcapone was carried out with ^1^H,^13^C‐HMBC spectra and a titration with NaOD (Figures S4 and S5). In the STD‐NMR spectrum, the strongest transfer of saturation was observed to the proton at position 4 of Tolcapone (assigned to 100% STD effect). Additionally, the difference spectrum shows the second strongest signal for the protons at position 3 (36%). Considering that this position contains two chemically equivalent protons, it is likely one proton receives higher saturation than the other one. These results are in agreement with binding pose II, where the proton at position 5 is further away from the protein and thus receives less saturation transfer. Taken together, pose II was identified as the dominant orientation in solution. Binding of Tolcapone to LecA is therefore driven by the central interactions of the catechol moiety with the protein‐bound calcium ion, an additional hydrogen bonding or dipole interaction established by the nitro group and a possible interaction of the carbonyl group with the protein. Furthermore, the tolyl residue presumably engages in hydrophobic interactions as seen by saturation transfer to its protons. Assuming a similar binding mode for the nitro‐catechol, the vinyl‐containing Nitecapone and Entacapone lack a hydrogen bond acceptor in the same position as the ketone in Tolcapone. Furthermore, possible clashes by their branched substituents with the protein surface could serve as an explanation for the lack of binding to LecA (Table 1).

### Screening of Tolcapone Derivatives

**Figure 4:**
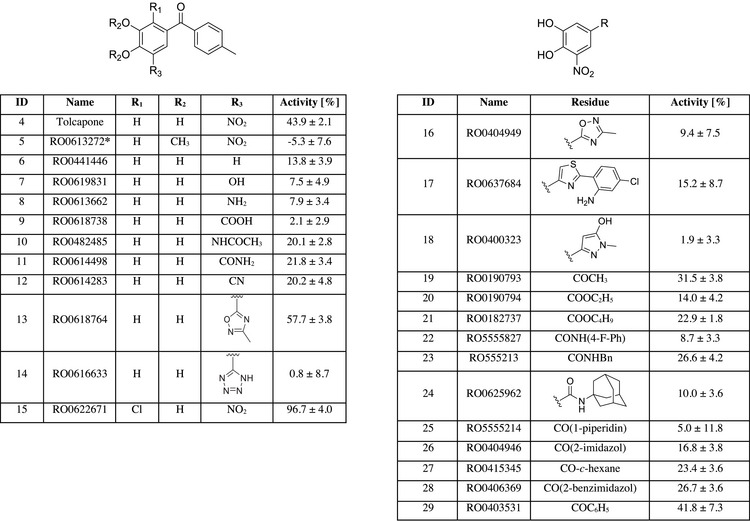
Selected examples highlight key lessons learned from the screening of 3267 catechols in the competitive binding assay. Activity is depicted as % inhibition at a compound concentration of 100 µM (125 µM when marked with *) relative to 500 µM *p*NPGal. Averages and standard deviations were calculated from three replicates (100 µM) or four replicates (125 µM).

Next, we screened a library of catechols from F. Hoffmann‐La Roche, the developer of Tolcapone, in the competitive binding assay to establish a structure–activity relationship. To this end, two sets of compounds with a molecular cut‐off of 500 Da were chosen: a first set of 342 compounds contained derivatives of Tolcapone, while a second set of 3222 compounds comprised catechols covering a larger chemical space. Some compounds were part of both sets, which resulted in 3267 unique compounds tested for LecA inhibition. Tests were run in a 384‐well‐plate format at three concentrations (first set at 125, 32, and 4 µM, second set at 100, 25, and 3 µM) in presence of 10% DMSO and 250 µM 2‐mercaptoethanol to prevent interference by a potential unspecific reactivity of electron‐rich catechols as established earlier.^[^ ^30^ ^]^ Inhibitory activity is reported as relative potency (% inhibition) compared to the known LecA binder *p*NPGal at 500 µM, which was defined as 100% inhibition. We investigated the structure‐activity relationship (SAR) of this data using matched molecular pair analysis.^[^ ^33^ ^]^ Key SAR trends are summarized in Figure 4.

The crucial role of the calcium complexation by the free catechol was underlined by the complete lack of activity of (partially) protected compounds such as **5** with a methyl protected catechol. Furthermore, replacement of the nitro group by various other functionalities (**6**–**14**) resulted in a loss of activity with the notable exception of oxadiazole **13**. Especially, the lower activity of nitrile **12** is noteworthy considering the cyano group is both strongly electron‐withdrawing and a hydrogen bond acceptor. The lack of affinity by compounds containing other hydrogen bond acceptors shows that the contributions of the nitro group toward binding are likely twofold: it serves as an interaction partner with Asn107 and reduces the electron density of the catechol, which was previously shown to be crucial. In line with the lower affinity of Opicapone compared to Tolcapone, the replacement of the ketone with small heterocycles abolished the activity (**16**–**18**). To quantify the contributions of additional residues to the binding, acetophenone **19** can serve as a reference point. No gain of affinity is achieved by converting the ketone in **19** to esters or amides (**20**–**25**). Additionally, replacement of the phenyl ring with other (hetero‐)cycles was generally disfavored (**26**–**29**). Encouragingly, the most potent derivatives (e.g., **15**, Figure 4) showed almost identical activity as the carbohydrate‐based reference *p*NPGal at 500 µM.

Compounds displaying >50% inhibition were selected for a more detailed characterization, while those with > 105% inhibition were excluded as false positives due to precipitation. This resulted in 48 compounds selected for an extended dose‐response analysis in the competitive binding assay based on fluorescence polarization (Table S1). The purity of all compounds was quantified by LCMS and 43/48 test molecules showed a purity of > 95%. One compound was excluded due to insufficient purity resulting in a final number of 47 compounds tested. The final DMSO concentration was increased to 20% because higher inhibitor concentrations were required to obtain full titration curves. 2‐Mercaptoethanol was excluded from the assay since all compounds possess electron‐deficient catechol moieties, which previously did not lead to unspecific reactivity.

**Figure 5:**
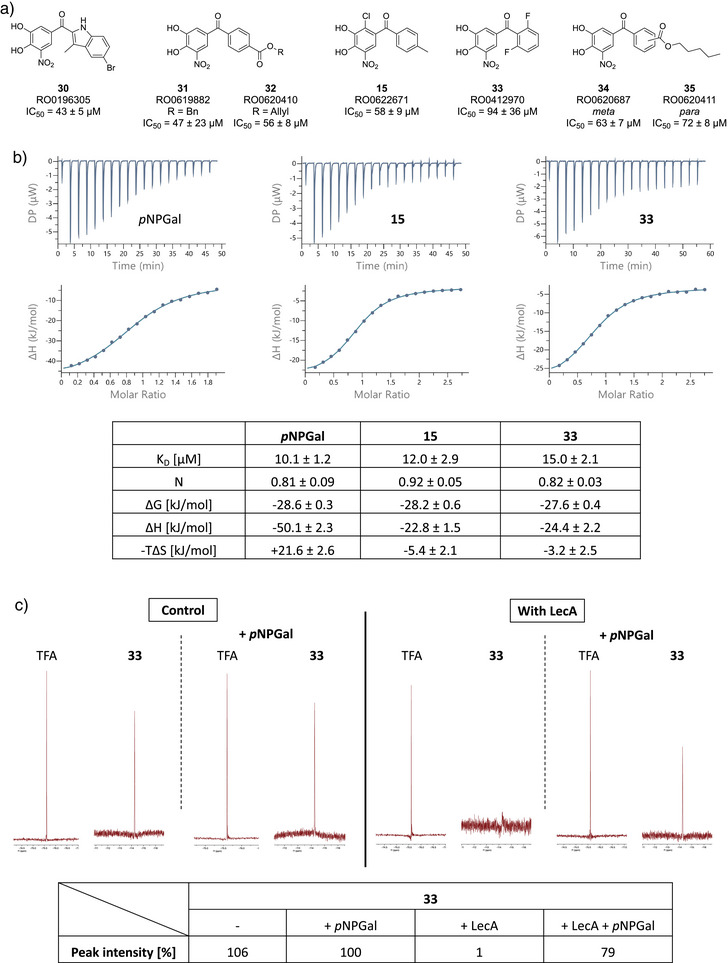
a) Catechols with an IC_50_ below 100 µM determined in the competitive binding assay (averages and standard deviations of three experiments), b) ITC titration data and thermodynamic parameters of catechols **15** and **33** in comparison to the modified carbohydrate *p*NPGal (based on three experiments per compound), and c) binding of **33** (50 µM) to LecA (100 µM) by ligand‐observed ^19^F NMR spectroscopy and its competitive displacement with addition of *p*NPGal (1 mM).

Trends observed in the initial screening were also reflected in the determined IC_50_ values. In total, 26 of the 47 compounds showed a stronger LecA inhibition than Tolcapone and seven compounds reached IC_50_ values below 100 µM (Figure 5a), which was preserved in the presence of detergent (0.01% Triton X‐100) to account for false positives from possible compound aggregation (Table S2). The replacement of the nitro group in Tolcapone with an oxadiazole in **13** resulted in a higher IC_50_ value compared to Tolcapone (346 ± 36 µM versus 257 ± 40 µM), which was in contrast to the somewhat higher inhibition in the initial screening set (Figure 4). The phenyl ring as a second aromatic system was generally preferred over other aromatic substituents. As one exception, brominated indole **30** (IC_50_  = 43 µM) was among the best performing compounds. Here, the bromine substituent appeared to be important since the derivative lacking the halogen substituent showed a 10‐fold reduced inhibition. Another interesting modification was the replacement of the ketone in Tolcapone with a diketone. Both compounds with this modification showed a higher affinity than Tolcapone (RO0619291, 127.7 µM and RO0621161, 152.8 µM; Table S1). Substitution at the second phenyl ring with small substituents was favored in the ortho position over para and meta as highlighted by **33**. A possible explanation for this observation is a substituent‐induced change in the angle of the two phenyl ring planes of the benzophenone motif, which may allow improved hydrophobic interactions with the protein surface. Compound **15** (IC_50_ = 58 µM) bearing an additional chlorine substituent at the catechol ring may profit from the same effect. Furthermore, the electron‐density of the catechol is further decreased by the electronegative chlorine and the small pocket in LecA usually occupied by galactose‐OH6 could get partially occupied. Larger substituents at the distal phenyl ring were preferred in the para position over meta analogs, with an additional preference of esters over amides. This is evidenced by the two potent compounds **31** and **32**, which were among the most potent out of the tested compounds (IC_50_  = 47 µM, IC_50_ = 56 µM, respectively).

To confirm the high inhibition of LecA by these non‐carbohydrate glycomimetics, **15** and **33** were then further tested in an orthogonal binding assay by isothermal titration calorimetry (Figure 5b). As a control, the binding of *p*NPGal to LecA was determined and a *K*_D_ = 10.1 µM was in good agreement with the value of 14.1 µM determined by Kadam et al.^[^ ^20^ ^]^ The thermodynamics of the *p*NPGal – LecA interaction are coined by a high binding enthalpy of ‐50.1 kJ/mol, which is offset by an entropy cost (‐T△*S* = +21.6 kJ/mol). Gratifyingly, the two analyzed catechols showed *K*_D_ values of 12–15 µM, which is in the same range as the potent glycoside *p*NPGal. Interestingly, while their binding enthalpy is reduced (△*H*  =  ‐22.8 to ‐24.4 kJ/mol), the binding of both catechols is also driven by favourable entropic contributions (‐T△*S* = ‐5.4 and ‐3.2 kJ/mol). This is a major difference compared to carbohydrate‐based ligands and reflects the more hydrophobic character of the interaction.

The presence of two fluorine atoms in the high affinity ligand **33** enabled another orthogonal LecA binding experiment (Figure 5c). In analogy to Denavit et al.,^[^ ^34^ ^]^ ligand‐observed ^19^F NMR spectra of 50 µM **33** were recorded in absence and in presence of LecA (100 µM). In the presence of LecA, a strongly reduced ^19^F‐NMR peak intensity of **33** down to the noise level highlights potent binding to LecA. Importantly, the ligand's fluorine signal was restored when *p*NPGal was added as competitor molecule, indicating the specific and reversible catechol – LecA interaction.

### X‐ray Crystallography of LecA in Complex with Tolcapone Derivatives

**Figure 6:**
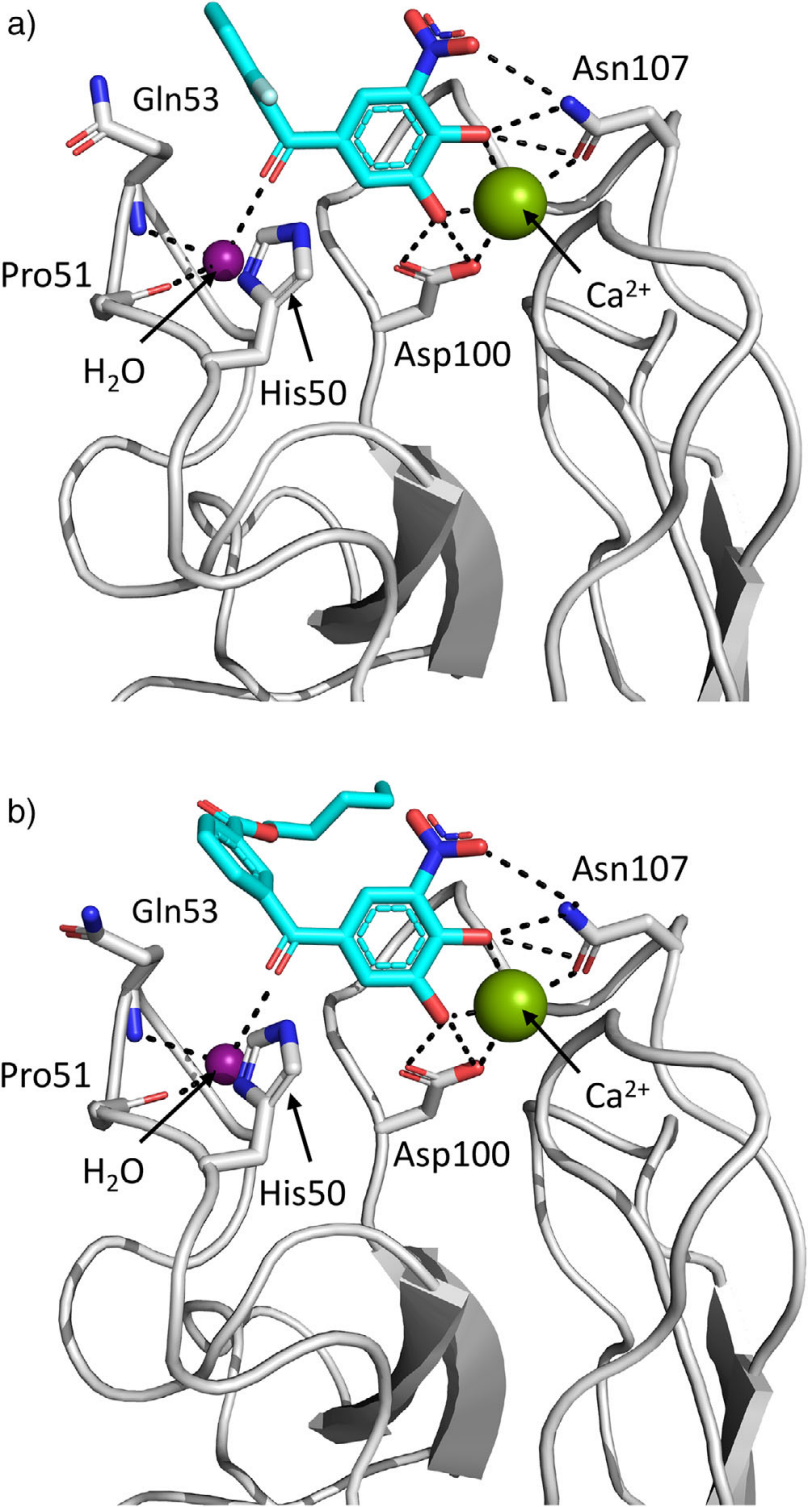
Crystal structures of LecA complexed with a) Catechol **33** (pdb code 9I7Z) and b) Catechol **34** (pdb code 9I80). Dashed lines indicated hydrogen bonding distance or metal complexation.

Additional X‐ray crystallography studies were conducted with selected derivatives of Tolcapone. Co‐crystal structures of **33** (pdb code 9I7Z) and **34** (pdb code 9I80) were solved at a resolution of 1.85 and 1.95 Å, respectively (Figure 6). In contrast to Tolcapone, here only single binding poses were observed for both compounds and no crystal contacts were found between neighboring asymmetric units. Gratifyingly, both compounds bound to LecA in a similar fashion to Tolcapone in binding pose II (Figure 2c), which was determined to be the dominant pose in solution by STD‐NMR (Figure 3). Consequently, the key interactions of calcium complexation and H bonding to Asp100 and Asn107 for the catechol moiety are the same as discussed above. A notable difference is the observed presence of the usually conserved water molecule, which was absent from binding pose II in the crystal structure of Tolcapone. As hypothesized before, the ketone of both compounds is in the correct position to form a water‐mediated hydrogen bond to the backbone of Pro51 and Gln53. It thereby acts as a mimetic of the OH6 of galactose. The high torsion of phenyl rings in **33** allows for improved hydrophobic interactions with the side chain of Gln53 (Figure 6a), in line with the entropic contributions determined by ITC. However, the distance of the carbonyl to the Nτ of His50 is increased (3.8 Å) compared to the distance observed for Tolcapone (3.3 Å). In contrast to this, the distance is also 3.3 Å in the case of **34** (Figure 6b). Here, the angle of the ester residue varied between the observed monomers with the alkyl chain aligning with the protein surface.

The molecules evaluated in this work originate from a COMT inhibitor development program. Therefore, it is not surprising that these compounds are also inhibitors of this enzyme. While this inhibition could in principle lead to side effects, it would on the other hand also be beneficial for compound stability and prevent its inactivation. Furthermore, tolcapone is used as a long‐term treatment in Parkinson's disease with the acceptable adverse effects of an approved drug. Thus, serious adverse effects seem unlikely during a short‐term anti‐infective regime. On top, none of these compounds have been developed for LecA. Therefore, a focus on selectivity during lead optimization holds additional promise to further strengthen their pharmacodynamic profile.

## Conclusion

In our efforts to find potent non‐carbohydrate glycomimetic lectin inhibitors, we investigated commercially available nitro‐catechols for their inhibition of LecA. A substantial increase in affinity of the Parkinson's drug Tolcapone over the initial screening hits was determined by direct (SPR) and competitive (FP) binding assays, while other drugs of this class proved inactive. Tolcapone was then crystallized with LecA to reveal its interactions at atomic level. With the aid of STD NMR spectroscopy, the dominant binding pose in solution could be identified. Important interactions of Tolcapone with LecA include calcium complexation through the catechol moiety, multiple direct and indirect hydrogen bonds to Asp100, Asn107, Pro51, and Gln53 as well as hydrophobic contacts established by the tolyl residue. The structure–activity relationship of (acylated) catechols was investigated by initial screening of a Roche library of 3267 compounds in our competitive LecA inhibition assay. 48 potent derivatives were selected for extended dose‐response analysis yielding several compounds with double‐digit micromolar IC_50_ values. Notably, these non‐carbohydrate catechol glycomimetics now showed the same affinity range as conventional carbohydrate‐based LecA inhibitors. Isothermal microcalorimetry revealed that the binding of catechols is driven by both enthalpy and entropy, which is in contrast to the enthalpy‐driven binding of *p*NPGal. While ^19^F NMR spectroscopy highlighted the competitive and reversible binding of a fluorine‐bearing nitro‐catechol, crystal structures obtained with two Tolcapone derivatives unambiguously revealed their binding modes and key interactions with LecA.

In this work, we demonstrated that non‐carbohydrate fragments with weak lectin binding activity can be optimized into potent non‐carbohydrate glycomimetics. In contrast to glycosides, these molecules occupy a more druglike chemical space – with several of the tested analogues being actual approved drugs. Finally, the structure–activity relationship paired with structural data for LecA binding of catechols will guide future optimization and selectivity assessment to generate a lead structure to fight antimicrobial resistance.

## Supporting Information

Experimental details, protocols on assays and diverse figures can be found in the electronic supporting information.

## Conflict of Interests

B.K., C.L., and U.G. are full employees of F. Hoffmann‐La Roche. The remaining authors have no conflicts of interest to declare.

## Supporting information



Supporting information

## Data Availability

The data that support the findings of this study are available in the Supporting Information of this article.
