# Supplementary material for: The Parkinson's Disease Drug Tolcapone and Analogues are Potent Glycomimetic Lectin Inhibitors of Pseudomonas aeruginosa LecA
Source: Angew Chem Int Ed Engl. 2025 Nov 2;64(50):e202508864. doi: 10.1002/anie.202508864 (PMC12684298; doi:10.1002/anie.202508864)
Supplement: Supplementary file 1 — Supporting information [file ANIE-64-e202508864-s001.docx]

**The Parkinson's Disease Drug Tolcapone and Analogues are Potent Glycomimetic Lectin Inhibitors of *Pseudomonas aeruginosa* LecA**

Steffen Leusmann^#1,2,3^, Eike Siebs^#1,2,3^, Sakonwan Kuhaudomlarp^4,5^, Annabelle Varrot^4^ Anne Imberty^4^, Bernd Kuhn^6^, Christian Lerner^6^, Uwe Grether^6^, Alexander Titz^*1,2,3^

^1^ Chemical Biology of Carbohydrates (CBCH), Helmholtz-Institute for Pharmaceutical Research Saarland (HIPS), Helmholtz Centre for Infection Research, 66123 Saarbrücken, Germany

^2^ Department of Chemistry, PharmaScienceHub (PSH), Saarland University, 66123 Saarbrücken, Germany

^3^ Deutsches Zentrum für Infektionsforschung (DZIF), Standort Hannover-Braunschweig, 38124 Braunschweig, Germany

^4^ Univ. Grenoble Alpes, Centre National de la Recherche Scientifique (CNRS), Centre de Recherches sur les Macromolécules Végétales (CERMAV), 38000 Grenoble, France

^5^ Department of Biochemistry, Faculty of Science, Mahidol University, Bangkok 10400, Thailand

^6^ Pharmaceutical Research and Early Development (pRED), Roche Innovation Center Basel, F. Hoffmann-La Roche AG, 4070 Basel, Switzerland

^#^ contributed equally, ^*^ alexander.titz@helmholtz-hips.de

Table of Contents

[Experimental methods 3](#_Toc210314561)

[General experimental details 3](#_Toc210314562)

[Recombinant LecA expression and purification 3](#_Toc210314563)

[Competitive Binding Assay using Fluorescence Polarization (FP) 4](#_Toc210314564)

[Library screening using the FP Assay 4](#_Toc210314565)

[Surface plasmon resonance (SPR) 5](#_Toc210314566)

[Crystallization and structure determination 5](#_Toc210314567)

[^1^H STD NMR spectroscopy 6](#_Toc210314568)

[Isothermal titration calorimetry (ITC) 7](#_Toc210314569)

[^19^F NMR spectroscopy 7](#_Toc210314570)

[Purity control of compounds selected for dose-response tests 7](#_Toc210314571)

[Tables and Experimental Data 9](#_Toc210314572)

[Figure S1: Individual replicates of SPR experiments of Tolcapone binding to LecA. 9](#_Toc210314573)

[Figure S2: Binding mode of Tolcapone compared to 3-cyanocatechol and d‑galactose in LecA. 10](#_Toc210314574)

[Figure S3: Rigid body movement of LecA around Gln53 depending on the binding pose of Tolcapone 11](#_Toc210314575)

[Figure S4: ^1^H,^13^C-HMBC NMR spectrum of Tolcapone in DMSO-d_6_ 12](#_Toc210314576)

[Figure S5: Titration of Tolcapone with NaOD in DMSO-d_6_ observed by ^1^H NMR spectroscopy 13](#_Toc210314577)

[Table S1. Compounds evaluated in dose-response analyses and calculated IC_50_s 14](#_Toc210314578)

[Table S2: IC_50_ determination of selected catechols with LecA in the presence of Triton X-100 (FP assay) 17](#_Toc210314579)

[Figure S6: Isothermal titration calorimetry 18](#_Toc210314580)

[Figure S7: ^19^F NMR spectra of catechol 33 (RO0412970) 21](#_Toc210314581)

[Table S3: ^19^F NMR peak intensities and signal percentage 23](#_Toc210314582)

[Table S4: Experimental data and statistics of the X-ray crystallography 24](#_Toc210314583)

[HPLC-UV purity determination of compounds evaluated in the dose-response panel 25](#_Toc210314584)

[References 48](#_Toc210314585)

# Experimental methods

## General experimental details

Commercial chemicals and solvents were used without further purification. Deuterated solvents were bought from Eurisotop (Saarbrücken, Germany). Tolcapone was acquired from Selleckchem (99% purity), Entacapone from BLDpharm (95% purity), Opicapone from Cayman Chemical Company (98% purity) and Nitecapone from SigmaAldrich (98% purity). All NMR spectra were recorded on a Bruker Avance III 500 UltraShield spectrometer at 298 K.

## Recombinant LecA expression and purification

LecA for crystallisation and surface plasmon resonance experiments was obtained according to the procedure described by Kuhaudomlarp et al.^[1]^ Expression and purification of LecA for FP assays, ITC and NMR experiments was performed as previously described with small adjustments.^[2]^ Briefly, *E. coli* BL21 (DE3) harbouring the plasmid pET25pa11 was grown in LB medium (10 g NaCl, 10 g tryptone, 5 g yeast extract; adjusted to 1 L, pH 7.4) supplemented with ampicillin (100 µg/mL) at 37 ºC and 180 rpm until an OD_600_ of 0.5 – 0.6 was reached. IPTG was added (250 µM) to induce the expression and the culture was cultivated at 30 ºC and 180 rpm for 4 hours. The cells were harvested by centrifugation (4000 g, 10 min, 4 ºC), the supernatant was discarded and the pellet was washed with TBS/Ca (20 mM Tris, 147 mM NaCl, 2.6 mM KCl at pH 7.4 supplemented with 1 mM CaCl_2_). Afterwards, the pellet was resuspended in TBS/Ca, PMSF (1 mM) and lysozyme (0.4 mg/mL) were added and the cells lysed using a Microfluidizer (4 cycles). Following a centrifugation (42000 g, 60 min, 4 °C) to remove the cell debris, the supernatant was loaded onto a melibiose-modified Sepharose CL-6B column using an Äkta Start system.^[3,4]^ After washing out unbound proteins, bound LecA was eluted with d-galactose (100 mM) in TBS/Ca. The eluted fractions were extensively dialyzed against TBS/Ca buffer and the protein was stored at -20 ºC. The concentration was determined by UV spectroscopy at 280 nm (e = 27960 M^-1^cm^-1^, MW = 12893 g/mol). Alternatively, the protein was dialyzed against ddH_2_O and lyophilized.

## Competitive Binding Assay using Fluorescence Polarization (FP)

The competitive binding assay was performed in analogy to Joachim et al.^[2]^ 10 µL of a solution containing LecA (40 µM) and sulfo-Cy5-Gal (20 nM, synthesis described in Kuhaudomlarp, Siebs et al.^[5]^) in TBS/Ca buffer (20 mM Tris, 147 mM NaCl, 2.6 mM KCl at pH 7.4 supplemented with 1 mM CaCl_2_) were distributed in into wells of a black 384-well microtiter plate (Greiner Bio-One, Germany, cat no 781900). Then, 10 µL of serial dilutions of compounds (3 mM to 1.5 µM, dilution factor 0.5) in TBS/Ca with 40% DMSO were added in technical triplicates. Final concentrations after mixing were the following: 1.5 mM – 0.73 µM compound, 20 µM LecA, 10 nM sulfo-Cy5-Gal and 20% DMSO. The plate was sealed (EASYseal, Greiner Bio-One, cat no 676001), briefly centrifuged and incubated at room temperature in the dark under shaking conditions. Fluorescence intensity was measured after 1 h and 4 h using a PheraStar FS plate reader (BMG Labtech GmbH, Germany) with spectral filters for excitation at 590 nm and emission at 675 nm and polarization was calculated after blank values of 20 µM LecA in TBS/Ca buffer with 20% DMSO were subtracted from the test compounds. The data were analysed using the four-parameter variable slope model of the MARS Data Analysis software. The top and bottom plateau were assigned based on the control compound methyl α-d-galactoside and the data was reanalysed with these fixed values. If not mentioned otherwise, the experiment was repeated at least three times for each compound and the average and standard deviation are given. In case Triton X-100 was used it was added to the TBS/Ca buffer before the assay.

## Library screening using the FP Assay

The screening followed the same assay by Joachim et al.^[2]^ as described above. Compounds in DMSO (1 µL with the appropriate concentration) were plated using a robot into black 384-well microtiter plates (Greiner Bio-One, Germany, cat no 781900) by the Roche compound handling facility at four replicates per concentration and compound. A mixture of LecA, sulfo-Cy5-Gal and 2-mercaptoethanol in TBS/Ca (20 mM Tris, 147 mM NaCl, 2.6 mM KCl at pH 7.4 supplemented with 1 mM CaCl_2_) with DMSO was added at HIPS. Final concentrations in both screening rounds were the following: 5 µM LecA, 250 µM 2-mercaptoethanol, 10 nM sulfo-Cy5-Gal and 10% DMSO. Final concentrations of the 342 compounds of the first screening round were 125 µM, 32 µM and 4 µM. The 3222 compounds of the second screening round were tested at final concentrations of 100 µM, 25 µM and 3 µM. The plate was sealed (EASYseal, Greiner Bio-One, cat no 676001), briefly centrifuged and incubated at room temperature in the dark under shaking conditions. Fluorescence intensities were measured after 1 h and 24 h using a PheraStar FS plate reader (BMG Labtech GmbH, Germany) with spectral filters for excitation at 590 nm and emission at 675 nm and polarization was calculated after blank values of 5 µM LecA in TBS/Ca buffer with 250 µM 2-mercaptoethanol and 10% DMSO were subtracted from the test compounds. The data were analysed using the MARS Data Analysis software. The relative binding potency (% inhibition) compared to *p*-nitrophenyl β-d-galactopyranoside at 500 µM (assigned to 100% inhibition, present in each individual plate) was determined. All stated values are the average including the standard deviation based on three replicates for 100 µM and four replicates in the case of compounds at 125 µM.

## Surface plasmon resonance (SPR)

Using a BIACORE X100, LecA was immobilised on a CM7 chip by standard amine coupling at 25 ºC as described previously.^[5]^ From a 200 mM solution of Tolcapone in 100% DMSO, a 10 mM solution in PBS (supplemented with 0.05% Tween20 and 100 µM CaCl_2_) was prepared. The final dilutions (0 – 2000 µM) were then prepared by dilution with running buffer (PBS supplemented with 0.05% Tween 20, 100 µM CaCl_2_ and 5% DMSO). Increasing concentrations of Tolcapone were injected onto the immobilised LecA in steady-state affinity studies (30 s association, 60 s dissociation, flow rate 30 µL/min). Data was corrected by subtraction of the reference channel (no immobilised LecA) and analysed with the BIACORE X100 evaluation software (version 2.0). The average binding affinity and standard deviation of three independent experiments is given.

## Crystallization and structure determination

Lyophilised LecA was dissolved in 20 mM Hepes pH 7.5, 100 mM NaCl and 100 µM CaCl_2_ to a concentration of 8 mg/mL. Dry soaking and vapor diffusion methods were used with hanging drops at 19 °C. First 0.5 µL of 200 mM Tolcapone in 100% DMSO were deposited on siliconized coverslips and allowed to dry at room temperature. Then, 1 µl of protein and 1 µl of crystallization solution were added. Single cube crystals were obtained in a few days from a solution containing 24% Peg 2K MME, 100 mM KSCN and 100 mM sodium acetate pH 4.5. A crystal was directly mounted in a cryoloop and flash-frozen in liquid nitrogen. For RO0412970 and RO0620687, 500 mM compound in 100% DMSO-d_6_ were diluted 1/10 with LecA (11.6 mg/mL) in 20 mM Hepes pH 7.5 with 100 µM CaCl_2_. Plate or cube crystals were obtained with 18% Peg 6K, 1 M LiCl and 100 mM sodium acetate pH 4.5 for RO0412970 and RO0620687, respectively. Single crystals were transferred in a solution containing 26% Peg 6K, 1 M LiCl and 100 mM sodium acetate pH 4.5 prior mounting in a cryoloop and flash freezing in liquid nitrogen. All diffraction data were collected at 100 K at 0.9786 Å wavelength at the Synchrotron SOLEIL (Saint Aubin, France) on Proxima-1 beamlines using an Eiger-X 16M detector.

XDS^[6]^ and XDSme^[7]^ were used to process the data and further steps were performed with CCP4, version 8^[8]^. Data quality statistics are summarized in Table S4.

The structures of LecA with Tolcapone and RO0412970 were solved by molecular replacement using PHASER and the chain A coordinates of PDB-ID 1OKO as search model searching for 2 and 4 molecules, respectively. For the structure of LecA-RO0620687, the tetramer of 1OKO was used as search model searching for 2 tetramers.^[9]^ The LecA-Tolcapone model was initially rebuild using ARP/WARP,^[10]^ whilst for the others Buccaneer was used.^[11]^ Then, all structures were refined with restrained maximum likelihood refinement using REFMAC 5.8^[12]^ and local NCS restraints iterated with manual rebuilding in Coot^[13]^. For LecA-RO0620687 intensity based twin refinement coupled with TLS were used in REFMAC 5.8. Five percent of the observations were set aside for cross-validation analysis, and hydrogen atoms were added in their riding positions and used for geometry and structure-factor calculations. Incorporation of the ligand was performed after inspection of the 2Fo-DFc weighted maps. The ligand libraries were constructed with Acedrg^[14]^ in CCP4i. The models were validated with the wwPDB Validation server: http://wwpdb-validation.wwpdb.org. The coordinates were deposited in the Protein Data Bank under code 8GUV, 9I7Z and 9I80.

All figures of LecA – ligand crystal structures were created using PyMOL 2.5.2.

## ^1^H STD NMR spectroscopy

A sample containing 20 µM LecA and 1.5 mM Tolcapone in deuterated PBS/Ca with 15% DMSO-d_6_ (142 mM NaCl, 10 mM Na_2_HPO_4_, 2.8 mM KCl, 1.8 mM KH_2_PO_4_ at pH 7.4, supplemented with 96 µM CaCl_2_) was irradiated at 0 ppm (on-resonance) and -40 ppm (off-resonance). 256 scans were recorded with a saturation time of 2 s, an acquisition time of 1 s and a relaxation delay of 4 s. The same experiment was repeated in absence of LecA to exclude STD effects due to direct saturation of Tolcapone. Spectra were analysed in MestReNova 14.2.0. The spectra were referenced based on the residual solvent peak of DMSO-d_6_ (2.50 ppm). Assignment of the resonances of Tolcapone was carried out with the help of 1H,13C- HMBC spectra and a titration of Tolcapone with NaOD (Figure S4-S6).

## Isothermal titration calorimetry (ITC)

Isothermal titration calorimetry was performed on a MicroCal PEAQ-ITC (Malvern Panalytical) at 25 ºC and 750 rpm stirring. The cell was filled with LecA (75 - 111 µM) in TBS/Ca buffer (20 mM Tris, 147 mM NaCl, 2.6 mM KCl at pH 7.4 supplemented with 1 mM CaCl_2_) with 20% DMSO and titrated with ligand (0.75 mM or 1.5 mM, 19 injections) in the same buffer. Data were analysed using the one binding site model in the MicroCal PEAQ-ITC analysis software (Malvern Panalytical). The first injection of each titration was excluded and the baseline subtracted for the final figures. Three titration per compound were performed and the average as well as standard deviation are given.

## ^19^F NMR spectroscopy

RO0412970 was mixed with trifluoroacetic acid (TFA) as internal standard in non-deuterated TBS/Ca (20 mM Tris, 147 mM NaCl, 2.6 mM KCl at pH 7.4 supplemented with 1 mM CaCl_2_) and 10% DMSO-d_6_. From this mixture, one sample with LecA and one without LecA (as control) were prepared. Final concentrations were 50 µM RO0412970, 100 µM TFA and, if present, 100 µM LecA. ^19^F NMR spectra (512 scans) were recorded and afterwards *para*-nitrophenyl β-d-galactopyranoside (*p*NPGal, 100 mM) in DMSO-d_6_ was added to both samples to a final conc. of 1 mM. Directly after the addition, spectra of both samples were recorded and the data was analysed with MestReNova 14.2.0. The spectra were referenced to TFA (-75.6 ppm) and the peak intensity of RO412970 compared to the internal standard TFA was calculated. The ratio of the sample without protein after addition of *p*NPGal was defined as 100%.

## Purity control of compounds selected for dose-response tests

Compound stock solutions of the 48 compounds selected for dose-response analysis (Table S3) were analysed by HPLC-UV/MS on a Thermo Dionex Ultimate 3000 HPLC coupled to a Bruker amaZon SL mass spectrometer with UV detection at 254 nm. A RP-18 column (100/2 Nucleoshell RP18plus, 2.7 µm from Macherey-Nagel, Germany) was used as stationary phase with water and acetonitrile each containing 0.1% formic acid as mobile phases. A gradient of 5 – 95% acetonitrile within 7 minutes was used with a flow of 600 µL/min. For quantification, a blank was measured after each sample, which was subsequently subtracted from the UV chromatogram of the respective sample using OpenChrom.^[15]^ Peaks were detected automatically or by hand and integration using the peak integrator trapezoid was caried out. UV Chromatograms are depicted below. Determined purity is given in Table S3.

# Tables and Experimental Data

## Figure S1: Individual replicates of SPR experiments of Tolcapone binding to LecA.

## Figure S2: Binding mode of Tolcapone compared to 3-cyanocatechol and d‑galactose in LecA.

(A) Overlay of the binding poses of 3-cyanocatechol (pink, pdb code 6YO3) and the two poses of Tolcapone observed in pdb code 8GUV (pose I, green and pose II, blue). (B) Overlay of the binding poses of d-galactose (pink, pdb code 1OKO) and the two protein-bound poses of Tolcapone observed in pdb code 8GUV (green and blue).

## Figure S3: Rigid body movement of LecA around Gln53 depending on the binding pose of Tolcapone

LecA and ligand of Pose I are shown in brown, LecA and ligand of Pose II in teal. The essential calcium ion of the binding site is shown in light green. PDB code 8GUV.


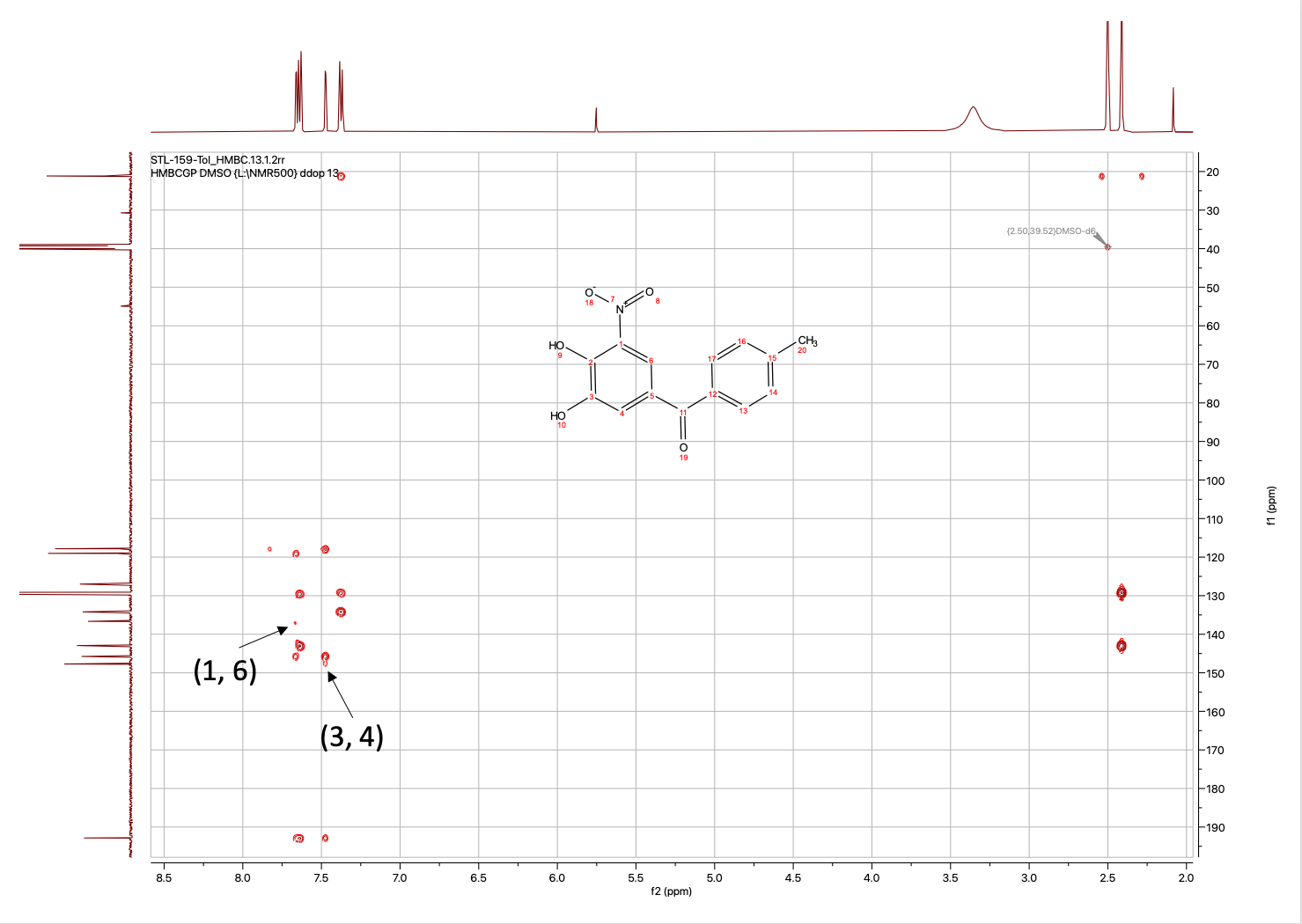


## Figure S4: ^1^H,^13^C-HMBC NMR spectrum of Tolcapone in DMSO-d_6_


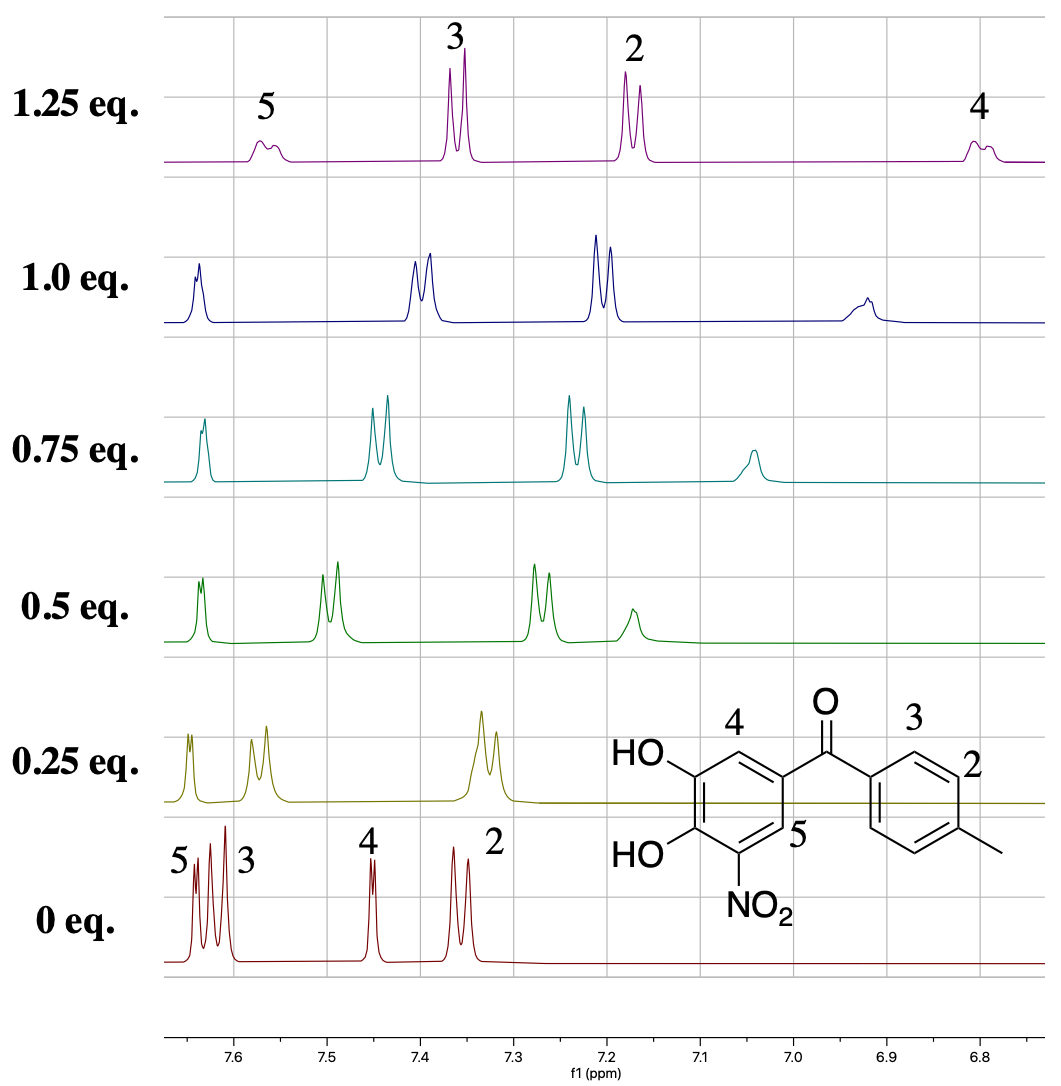


## Figure S5: Titration of Tolcapone with NaOD in DMSO-d_6_ observed by ^1^H NMR spectroscopy

3.8 mg Tolcapone (0.0139 mmol) were dissolved in ~470 µL DMSO-d_6_. A 1:10 dilution of 40% wt. NaOD in D_2_O was prepared and 2.5 µL of this dilution (equal to 0.25 eq. of Tolcapone) were added step-wise to the NMR sample. Spectra were recorded after each addition. Using MestReNova 14.2.0., the spectra were referenced to the peak H_2_O in DMSO (3.33 ppm) and stacked.

## Table S1. Compounds evaluated in dose-response analyses and calculated IC_50_s

| ID | Name | SMILES | IC_50_ [µM] | std. dev. [µM] | UV Purity |
| --- | --- | --- | --- | --- | --- |
|  | RO0412713 | [O-][N+](c1cc(C(c2cccc(F)c2)=O)cc(O)c1O)=O | 675 | 19 | 99% |
|  | RO0615662 | CC(C)(C)c(cc1)ccc1C(c(cc1O)cc([N+]([O-])=O)c1O)=O | 144 | 34 | 99% |
| 30 | RO0196305 | Cc1c(C(c(cc2[N+]([O-])=O)cc(O)c2O)=O)[nH]c(cc2)c1cc2Br | 43 | 5 | 97% |
|  | RO0413268 | N#Cc(cccc1)c1C(c(cc1O)cc([N+]([O-])=O)c1O)=O | 214 | 33 | 95% |
|  | RO0621004 | [O-][N+](c(cc(cc1O)C(c2cc(C(NCC3CC3)=O)ccc2)=O)c1O)=O | 363 | 38 | 99% |
| 33 | RO0412970 | [O-][N+](c1cc(C(c(c(F)ccc2)c2F)=O)cc(O)c1O)=O | 94 | 36 | 99% |
|  | RO0408176 | [O-][N+](c(cc(cc1O)C(c2cncc3ccccc23)=O)c1O)=O | 449 | 66 | 97% |
| 31 | RO0619882 | [O-][N+](c(cc(cc1O)C(c(cc2)ccc2C(OCc2ccccc2)=O)=O)c1O)=O | 47 | 23 | 99% |
|  | RO0619973 | COC(/C=C/c1cccc(C(c(cc2O)cc([N+]([O-])=O)c2O)=O)c1)=O | 258 | 31 | 88% |
|  | RO0627603 | CCCN(CCC)C(c(c([N+]([O-])=O)c(c(O)c1)O)c1Br)=O | 285 | 85 | 99% |
|  | RO0616411 | Cc(cc1)ccc1C(c(cc1O)cc(S(C(F)(F)F)(=O)=O)c1O)=O | 338 | 38 | 95% |
|  | RO0620573 | CCN(CC)C(c(cc1)ccc1C(c(cc1O)cc([N+]([O-])=O)c1O)=O)=O | 438 | 31 | 95% |
|  | RO0199733 | OC1=C(O)C([N+]([O-])=O)=CC(C2=CN3N=CSC3=N2)=C1 | 4112 | 2182 | 97% |
| 34 | RO0620687 | CCCCCOC(c1cccc(C(c(cc2O)cc([N+]([O-])=O)c2O)=O)c1)=O | 63 | 7 | 99% |
|  | RO0620808 | COC(c1cccc(C(c(cc2O)cc([N+]([O-])=O)c2O)=O)c1)=O | 279 | 18 | 99% |
|  | RO0624122 | COCCOCc(cc1)ccc1C(c(cc1O)cc([N+]([O-])=O)c1O)=O | 289 | 8 | 97% |
| 35 | RO0620411 | CCCCCOC(c(cc1)ccc1C(c(cc1O)cc([N+]([O-])=O)c1O)=O)=O | 72 | 8 | 96% |
| 32 | RO0620410 | C=CCOC(c(cc1)ccc1C(c(cc1O)cc([N+]([O-])=O)c1O)=O)=O | 56 | 8 | 99% |
|  | RO0620570 | CCCNC(c(cc1)ccc1C(c(cc1O)cc([N+]([O-])=O)c1O)=O)=O | 347 | 9 | 98% |
|  | RO0626783 | [O-][N+](c(cc(cc1O)C(c2cc(C(NC3CCCCC3)=O)ccc2)=O)c1O)=O | 430 | 23 | 99% |
|  | RO0407028 | [O-][N+](c1cc(C(c(cc2)ccc2F)=O)cc(O)c1O)=O | 370 | 40 | 99% |
| 13 | RO0618764 | Cc1noc(-c2cc(C(c3ccc(C)cc3)=O)cc(O)c2O)n1 | 346 | 36 | 99% |
|  | RO0621239 | CC(CCOC(c1cccc(C(c(cc2O)cc([N+]([O-])=O)c2O)=O)c1)=O)O | 192 | 32 | 94% |
|  | RO0620810 | C=CCOC(c1cccc(C(c(cc2O)cc([N+]([O-])=O)c2O)=O)c1)=O | 139 | 26 | 98% |
|  | RO0621109 | CC(C)OC(c1cccc(C(c(cc2O)cc([N+]([O-])=O)c2O)=O)c1)=O | 156 | 37 | 99% |
|  | RO0621116 | CCCN(CCC)C(c1cccc(C(c(cc2O)cc([N+]([O-])=O)c2O)=O)c1)=O | 173 | 36 | 99% |
|  | RO0621006 | CCCCNC(c1cccc(C(c(cc2O)cc([N+]([O-])=O)c2O)=O)c1)=O | 283 | 24 | 99% |
|  | RO0619291 | Cc(cc1)ccc1C(C(c(cc1O)cc([N+]([O-])=O)c1O)=O)=O | 128 | 21 | 99% |
|  | RO0621265 | COC(c(cc1)ccc1C(c(cc1O)cc([N+]([O-])=O)c1O)=O)=O | 330 | 60 | 98% |
|  | RO0626778 | CCCCOC(c1cccc(C(c(cc2O)cc([N+]([O-])=O)c2O)=O)c1)=O | 117 | 13 | 97% |
|  | RO0413301 | [O-][N+](c1cc(C(c(cccc2)c2Cl)=O)cc(O)c1O)=O | 108 | 19 | 98% |
|  | RO0622624 | [O-][N+](c1cc(C(c2cccc(C(OCCC(C(F)(F)F)O)=O)c2)=O)cc(O)c1O)=O | 135 | 36 | 97% |
|  | RO0615928 | CC(C)c(cc1)ccc1C(c(cc1O)cc([N+]([O-])=O)c1O)=O | 129 | 21 | 98% |
|  | RO0621161 | [O-][N+](c(cc(cc1O)C(C(c2ccccc2)=O)=O)c1O)=O | 153 | 23 | 99% |
|  | RO0626786 | [O-][N+](c(cc(cc1O)C(c2cc(C(NCc3ccccc3)=O)ccc2)=O)c1O)=O | 356 | 67 | 99% |
|  | RO0412734 | Cc(cccc1)c1C(c(cc1O)cc([N+]([O-])=O)c1O)=O | 116 | 7 | 99% |
|  | RO0621005 | CCCNC(c1cccc(C(c(cc2O)cc([N+]([O-])=O)c2O)=O)c1)=O | 422 | 63 | 99% |
|  | RO0621115 | CCN(CC)C(c1cccc(C(c(cc2O)cc([N+]([O-])=O)c2O)=O)c1)=O | 229 | 26 | 99% |
|  | RO0626777 | CC(C)(C)COC(c1cccc(C(c(cc2O)cc([N+]([O-])=O)c2O)=O)c1)=O | 206 | 41 | 74% |
|  | RO0403499 | O=C(C1=CC=NC=C1)C2=CC([N+]([O-])=O)=C(O)C(O)=C2 | 597 | 63 | 97% |
| 15 | RO0622671 | Cc(cc1)ccc1C(c(c(Cl)c1O)cc([N+]([O-])=O)c1O)=O | 58 | 9 | 95% |
|  | RO0413162 | [O-][N+](c1cc(C(c2c(C(F)(F)F)cccc2)=O)cc(O)c1O)=O | 128 | 25 | 99% |
|  | RO0620809 | CCOC(c1cccc(C(c(cc2O)cc([N+]([O-])=O)c2O)=O)c1)=O | 244 | 35 | 99% |
|  | RO0405919 | [O-][N+](c(cc(cc1O)C(c2ccnc3ccccc23)=O)c1O)=O | 174 | 13 | 98% |
|  | RO0614977 | Cc(cc1)ccc1C(c(c([N+]([O-])=O)c1O)ccc1O)=O | 154 | 23 | 98% |
|  | RO0403335 | Cc1c(C(c(cc2O)cc([N+]([O-])=O)c2O)=O)[nH]c2c1cccc2 | 434 | 61 | 68% |
|  | RO0623430 | [O-][N+](c(cc(cc1O)C(c2cc(CO)ccc2)=O)c1O)=O | 446 | 85 | 96% |

## Table S2: IC_50_ determination of selected catechols with LecA in the presence of Triton X-100 (FP assay)

| Compound | IC_50_ in absence of Triton X-100 [µM] | IC_50_ in presence of 0.057% Triton X-100 [µM] ^*^ | IC_50_ in presence of 0.01% Triton X-100 [µM] ^*^ |
| --- | --- | --- | --- |
| RO0196305 | 43 ± 5 | n.d. | 59 |
| RO0619882 | 47 ± 23 | 16 | 17 |
| RO0620410 | 56 ± 8 | 50 | 49 |
| RO0622671 | 58 ± 9 | 56 | 67 |
| RO0620687 | 63 ± 7 | 61 | 88 |
| RO0620411 | 73 ± 8 | 66 | n.d. |
| RO0412970 | 94 ± 36 | 91 | 81 |

**^*^** Assays with Triton performed in one replicate each, n.d. = not determined

## Figure S6: Isothermal titration calorimetry

|  | Repl. 1 | Repl. 2 | Repl. 3 | Average | Std. dev. |
| --- | --- | --- | --- | --- | --- |
| Conc. syringe [µM] | 750 | 750 | 750 | - | - |
| Conc. cell [µM] | 34 | 75 | 75 | - | - |
| N (sites) | 0.722 | 0.801 | 0.908 | 0.810 | 0.093 |
| K_D_ [µM] | 10.8 | 8.8 | 10.8 | 10.1 | 1.2 |
| 𝚫G [kJ/mol] | -28.4 | -28.9 | -28.4 | -28.6 | 0.3 |
| 𝚫H [kJ/mol] | -52.7 | -48.3 | -49.4 | -50.1 | 2.3 |
| -T𝚫S [kJ/mol] | 24.4 | 19.4 | 21.0 | 21.6 | 2.6 |

**Figure S6a: Individual ITC titrations of LecA with 4-nitrophenyl β-d-galactopyranoside (*p*NPGal)**

|  | Repl. 1 | Repl. 2 | Repl. 3 | Average | Std. dev. |
| --- | --- | --- | --- | --- | --- |
| Conc. syringe [µM] | 750 | 1500 | 1500 | - | - |
| Conc. cell [µM] | 75 | 111 | 110 | - | - |
| N (sites) | 0.947 | 0.957 | 0.862 | 0.922 | 0.052 |
| K_D_ [µM] | 15.1 | 9.3 | 11.5 | 12.0 | 2.9 |
| 𝚫G [kJ/mol] | -27.6 | -28.8 | -28.2 | -28.2 | 0.6 |
| 𝚫H [kJ/mol] | -24.1 | -21.1 | -23.1 | -22.8 | 1.5 |
| -T𝚫S [kJ/mol] | -3.5 | -7.7 | -5.1 | -5.4 | 2.1 |

**Figure S6b: Individual ITC titrations of LecA with catechol 15 (RO0622671)**

|  | Repl. 1 | Repl. 2 | Repl. 3 | Average | Std. dev. |
| --- | --- | --- | --- | --- | --- |
| Conc. syringe [µM] | 750 | 1500 | 1500 | - | - |
| Conc. cell [µM] | 75 | 105 | 104 | - | - |
| N (sites) | 0.846 | 0.788 | 0.827 | 0.820 | 0.030 |
| K_D_ [µM] | 13.2 | 14.4 | 17.3 | 15.0 | 2.1 |
| 𝚫G [kJ/mol] | -27.9 | -27.7 | -27.2 | -27.6 | 0.4 |
| 𝚫H [kJ/mol] | -22.2 | -24.4 | -26.5 | -24.4 | 2.2 |
| -T𝚫S [kJ/mol] | -5.7 | -3.3 | -0.7 | -3.2 | 2.5 |

**Figure S6c: Individual ITC titrations of LecA with catechol 33 (RO0412970)**

## Figure S7: ^19^F NMR spectra of catechol 33 (RO0412970)

**Figure S7a: ^19^F NMR spectrum of catechol 33**

**Figure S7b: ^19^F NMR spectrum of 33 in presence of *p*NPGal**

**Figure S7c: ^19^F NMR spectrum of 33 in presence of 100 µM LecA**

**Figure S7d: ^19^F NMR spectrum of 33 in presence of 100 µM LecA and *p*NPGal**

## Table S3: ^19^F NMR peak intensities and signal percentage

|  | Intensity TFA | Intensity catechol 33 | Ratio | Percentage |
| --- | --- | --- | --- | --- |
| No LecA, before *p*NPGal | 69,833,773,792 | 20,411,628,694 | 0.2923 | 106% |
| No LecA, after *p*NPGal | 63,175,004,482 | 17,384,245,550 | 0.2752 | 100 |
| LecA, before *p*NPGal | 66,217,595,493 | 236,024,213 | 0.0036 | 1% |
| LecA, after *p*NPGal | 57,333,209,902 | 12,386,244,562 | 0.2160 | 79% |

## Table S4: Experimental data and statistics of the X-ray crystallography

|  | **LecA-Tolcapone** | **LecA-RO0412970** | **LecA-RO0620687** |
| --- | --- | --- | --- |
| **Data Collection** |  |  |  |
| Beamline | Proxima1 (SOLEIL) | Proxima1(SOLEIL) | Proxima1(SOLEIL) |
| Wavelength (Å) | 0.9786 | 0.9786 | 0.9786 |
| Detector | Eiger-X 16M | Eiger-X 16M | Eiger-X 16M |
| Resolution (Å)^a^ | 42.80-1.32 (1.34-1.32) | 42.10-1.85 (1.89-1.85) | 47.14-1.95 (1.99-1.95) |
| Space Group | C222_1_ | P2_1_ | P4_1_ |
| a, b, c (Å) | 57.65, 164.63, 50.11 | 60.58, 50.73, 71.69 | 81.21, 81.21, 165.03 |
| α, β, γ (°) | 90.0, 90.0, 90.0 | 90.0, 100.01, 90.0 | 90.0, 90.0, 90.0 |
| Total observations^a^ | 722,712 | 201,020 (11,750) | 596,083 (32,008) |
| Unique reflections^a^ | 56,290 | 36,840 (2,274) | 77,579 (4,594) |
| Multiplicity^a^ | 12.8 (11.1) | 5.5 (5.2) | 7.7 (7.0) |
| Mean *I*/σ(*I*)^a^ | 25.2 (4.1) | 12.4 (2.4) | 8.7 (2.3) |
| Completeness (%)^a^ | 99.8 (96.4) | 99.9 (100) | 100 (99.9) |
| *R*_merge_^a,b^ | 0.052 (0.451) | 0.080 (0.563) | 0.126 (0.723) |
| *CC*_½_^a,c^ | 0.999 (0.960) | 0.998 (0.814) | 0.996 (0.798) |
| **Refinement** |  |  |  |
| Reflections: working/free^d^ | 56,272 / 2,793 | 34,977 / 1,850 | 73,603 / 3,915 |
| *R*_work_/ *R*_free_^e^ | 0.198 / 0.222 | 0.158 / 0.204 | 0.166 / 0.202 |
| R.m.s.d bonds (Å) | 0.0119 | 0.0139 | 0.0139 |
| R.m.s.d angles (°) | 1.722 | 1.821 | 1.925 |
| Nb Atoms protein/ligand^f^/waters | 1,826 / 62 / 282 | 3,601 / 84 / 399 | 7,256/ 207 / 911 |
| Mean *B*-factors (Å^2^): protein/ligand^f^/waters | 15.1 / 23.9 / 25.5 | 26.2 / 33.85 / 34.75 | 30.6 / 30.8 / 37.0 |
| Ramachandran plot (%):  allowed/favoured/outliers | 100 / 98 / 0 | 100 / 96.8 / 0 | 100 / 96.8 / 0 |
| PDB code | 8GUV | 9I7Z | 9I80 |

^a^ Values for the outer resolution shell are given in parentheses.

^b^ *R*_merge_ = ∑_hkl_ ∑_i_ |I_i_(hkl) − 〈I(hkl)〉|/ ∑_hkl_ ∑_i_I_i_(hkl).

^c^ *CC*_½_ is the correlation coefficient between symmetry-related intensities taken from random halves of the dataset.

^d^ The data set was split into "working" and "free" sets consisting of 95 and 5% of the data, respectively. The free set was not used for refinement.

^e^ The R-factors *R*_work_ and *R*_free_ are calculated as follows: *R* = ∑(| *F*_obs_ - *F*_calc_ |)/∑| *F*_obs_ |, where *F*_obs_ and *F*_calc_ are the observed and calculated structure factor amplitudes, respectively

^f^ refers to ligands bound in the active site and potential surface binding sites

## HPLC-UV purity determination of compounds evaluated in the dose-response panel


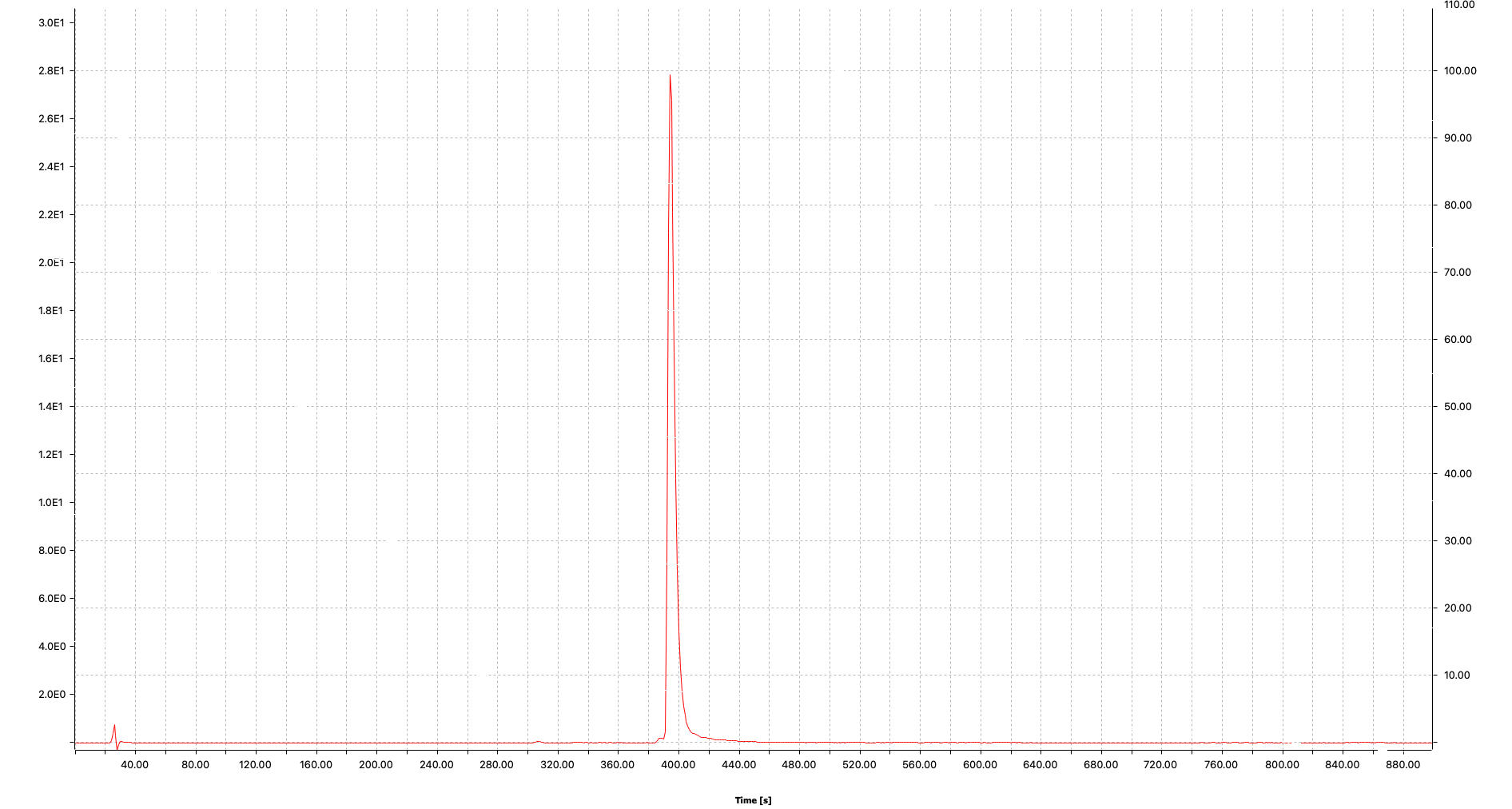


**Fig. S8: HPLC-UV chromatogram of RO0412713**


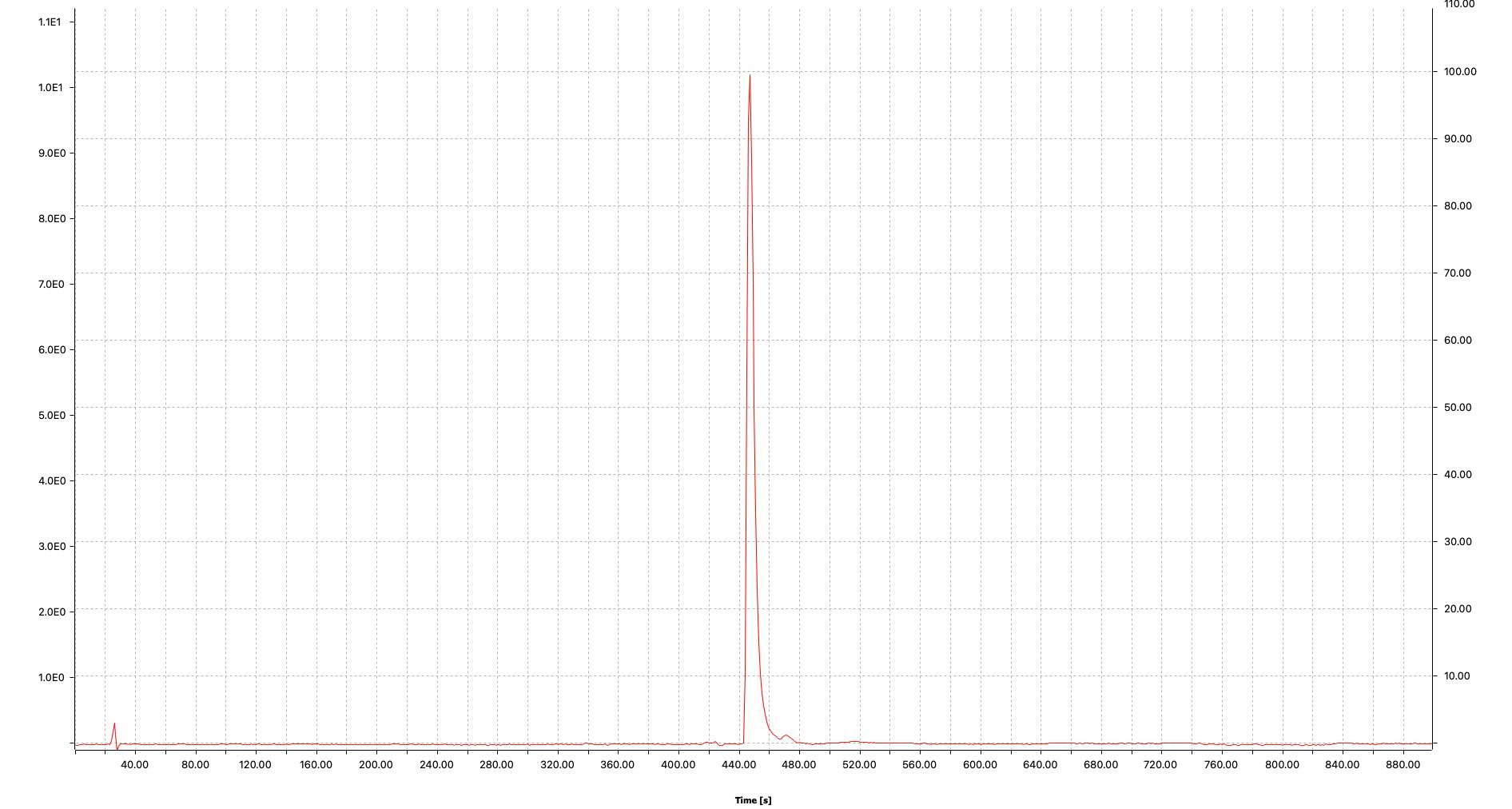


**Fig. S9: HPLC-UV chromatogram of RO0615662**


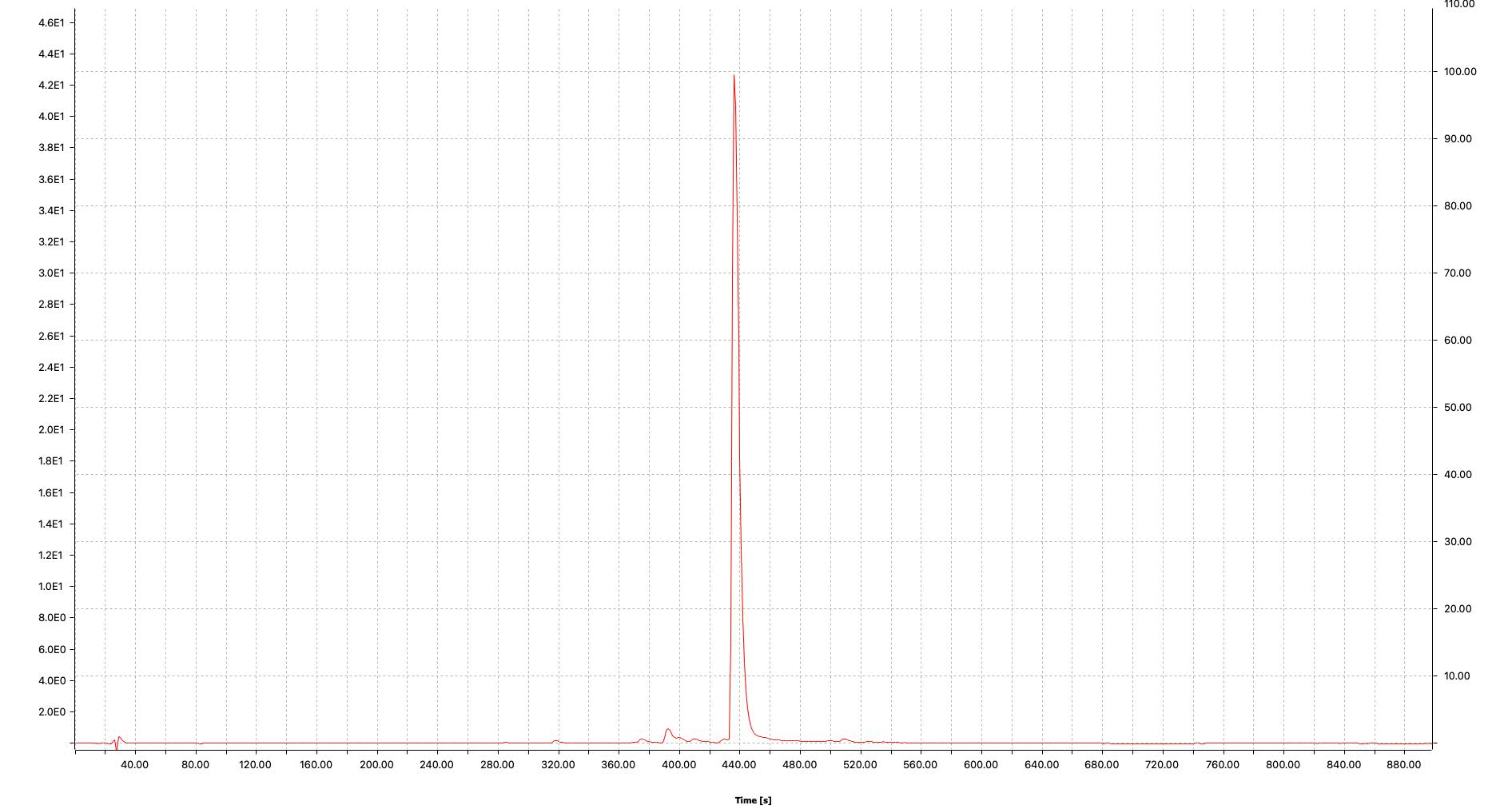


**Fig. S10: HPLC-UV chromatogram of RO0196305**


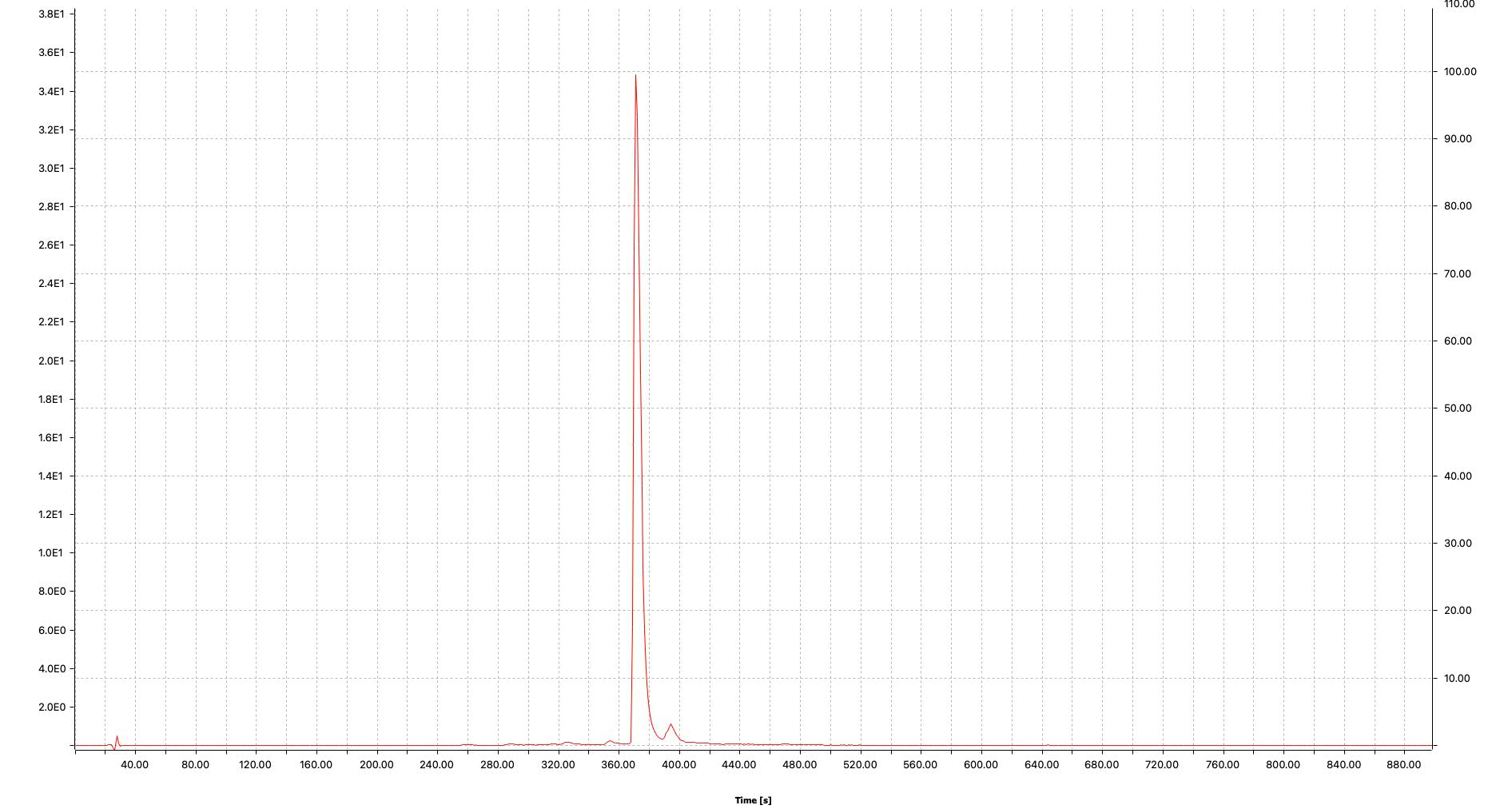


**Fig. S11: HPLC-UV chromatogram of RO0413268**


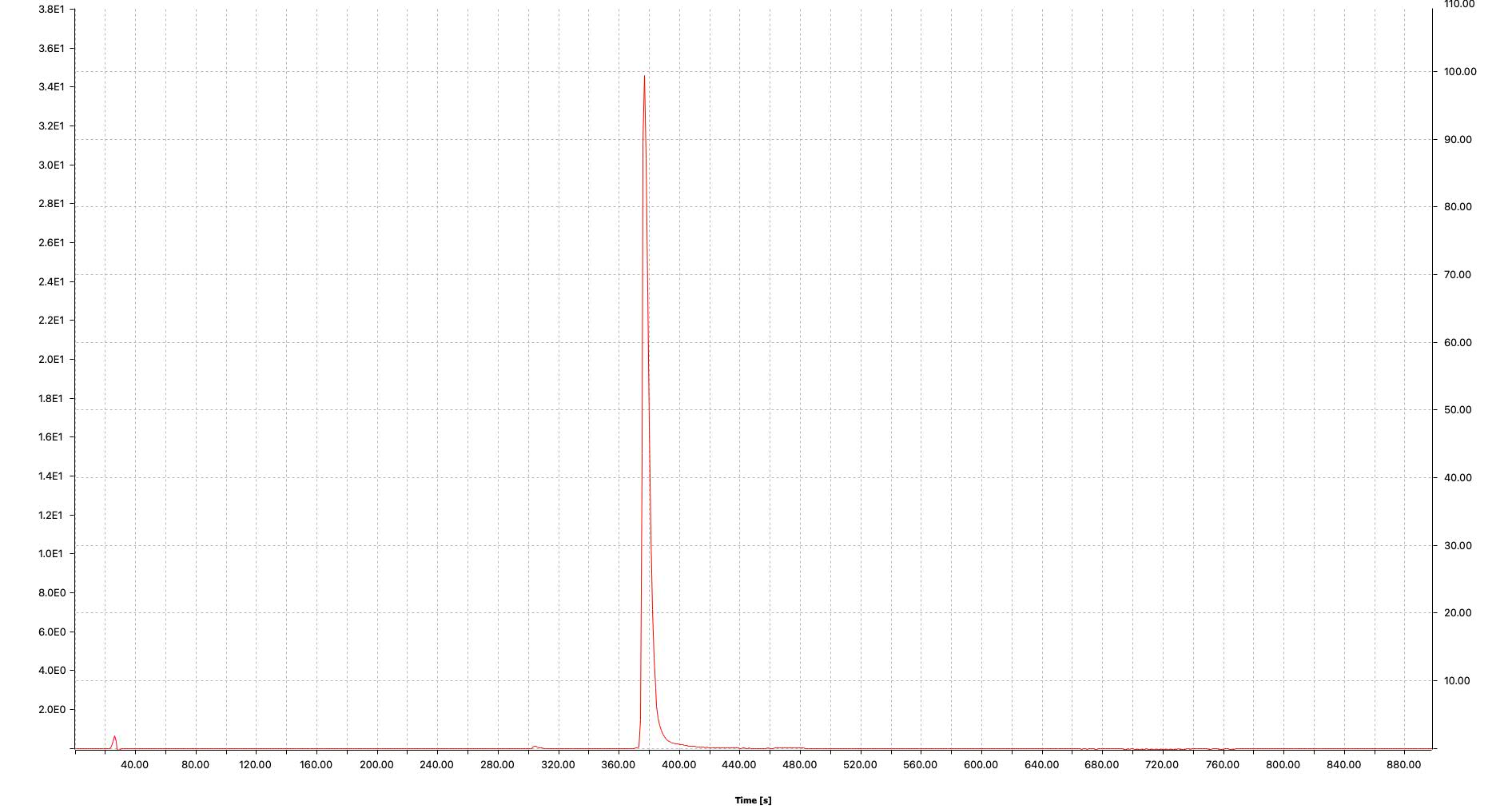


**Fig. S12: HPLC-UV chromatogram of RO0621004**


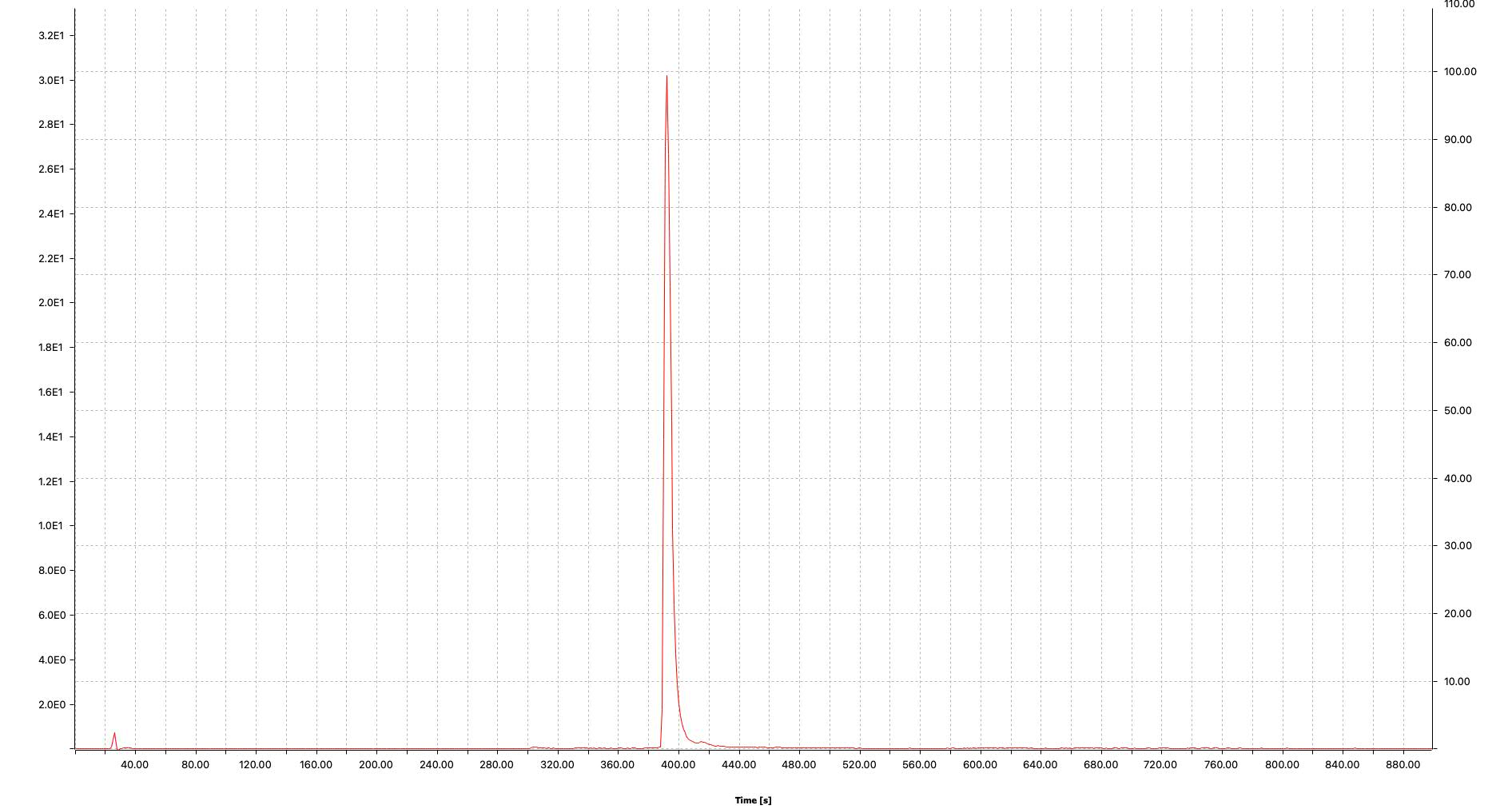


**Fig. S13: HPLC-UV chromatogram of RO0412970**


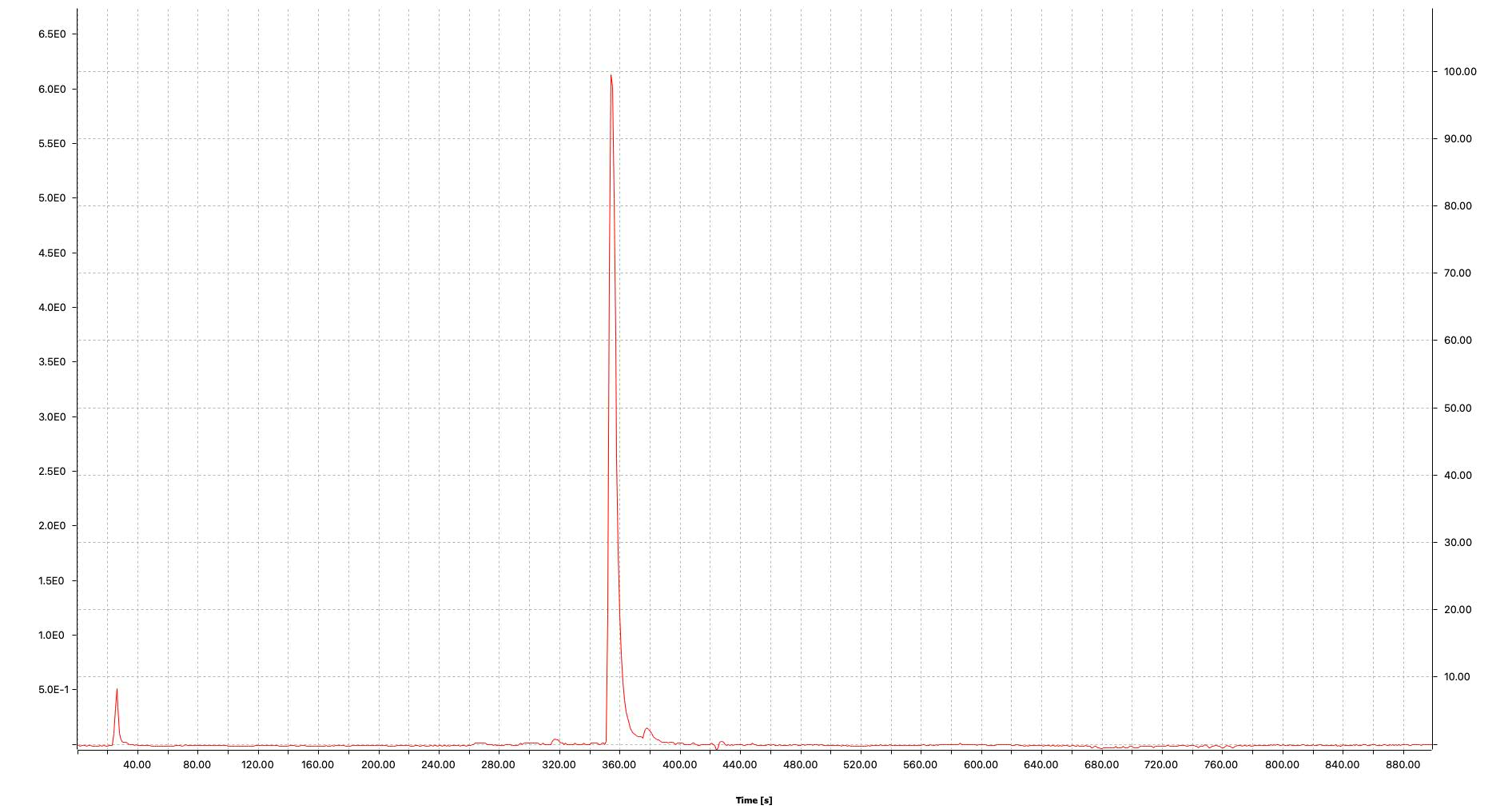


**Fig. S14: HPLC-UV chromatogram of RO0408176**


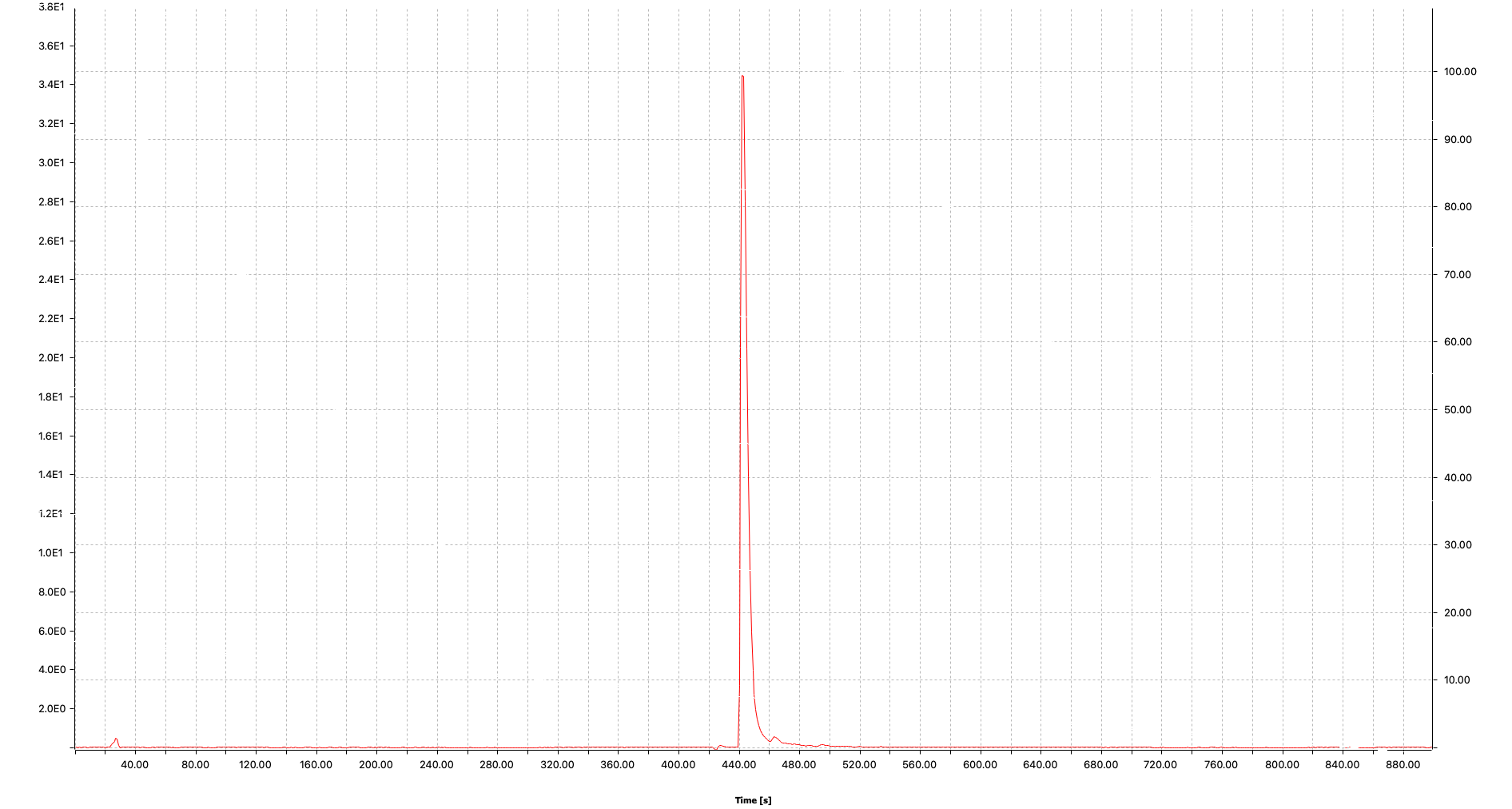


**Fig. S15: HPLC-UV chromatogram of RO0619882**


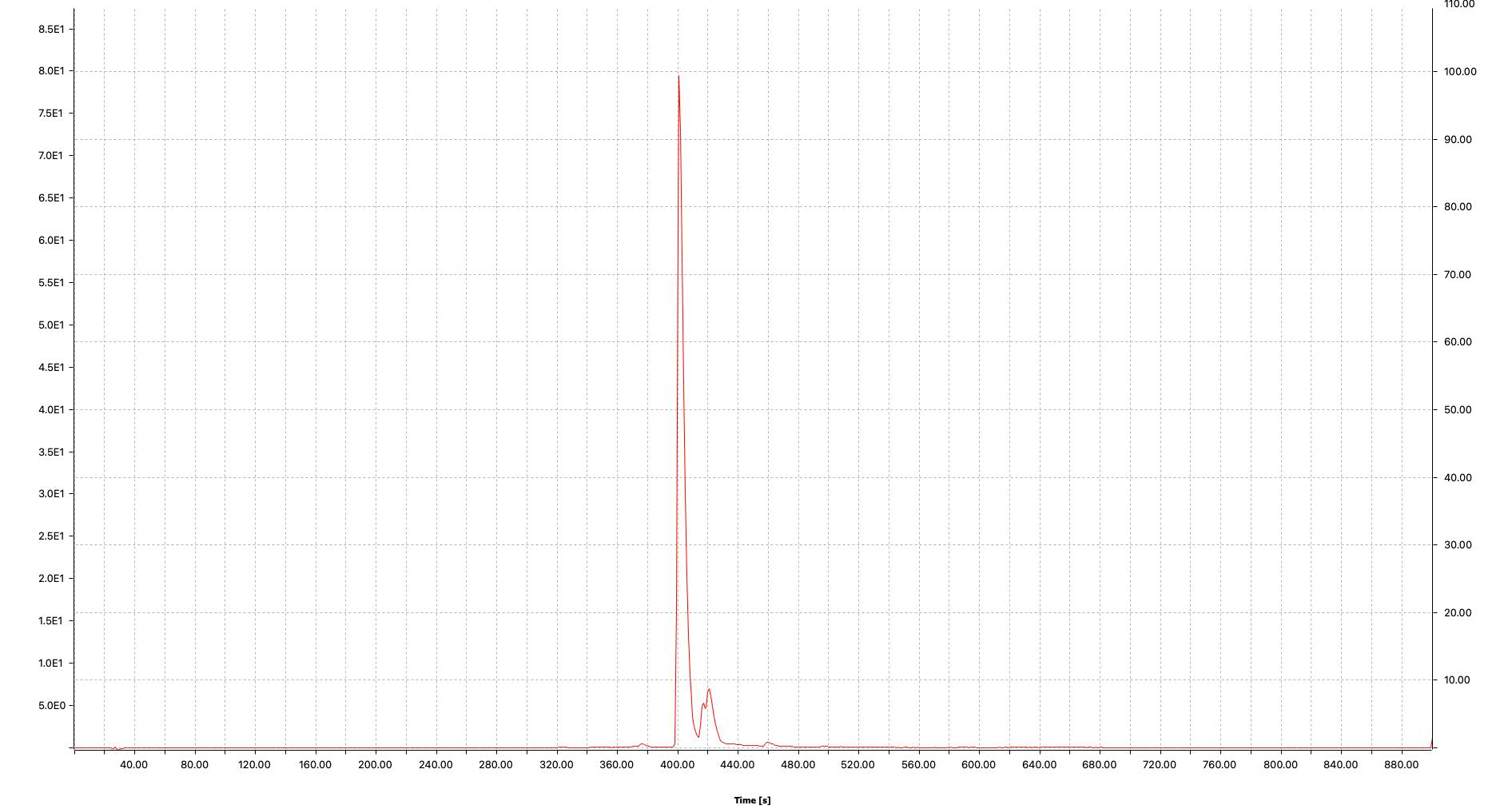


**Fig. S16: HPLC-UV chromatogram of RO0619973**


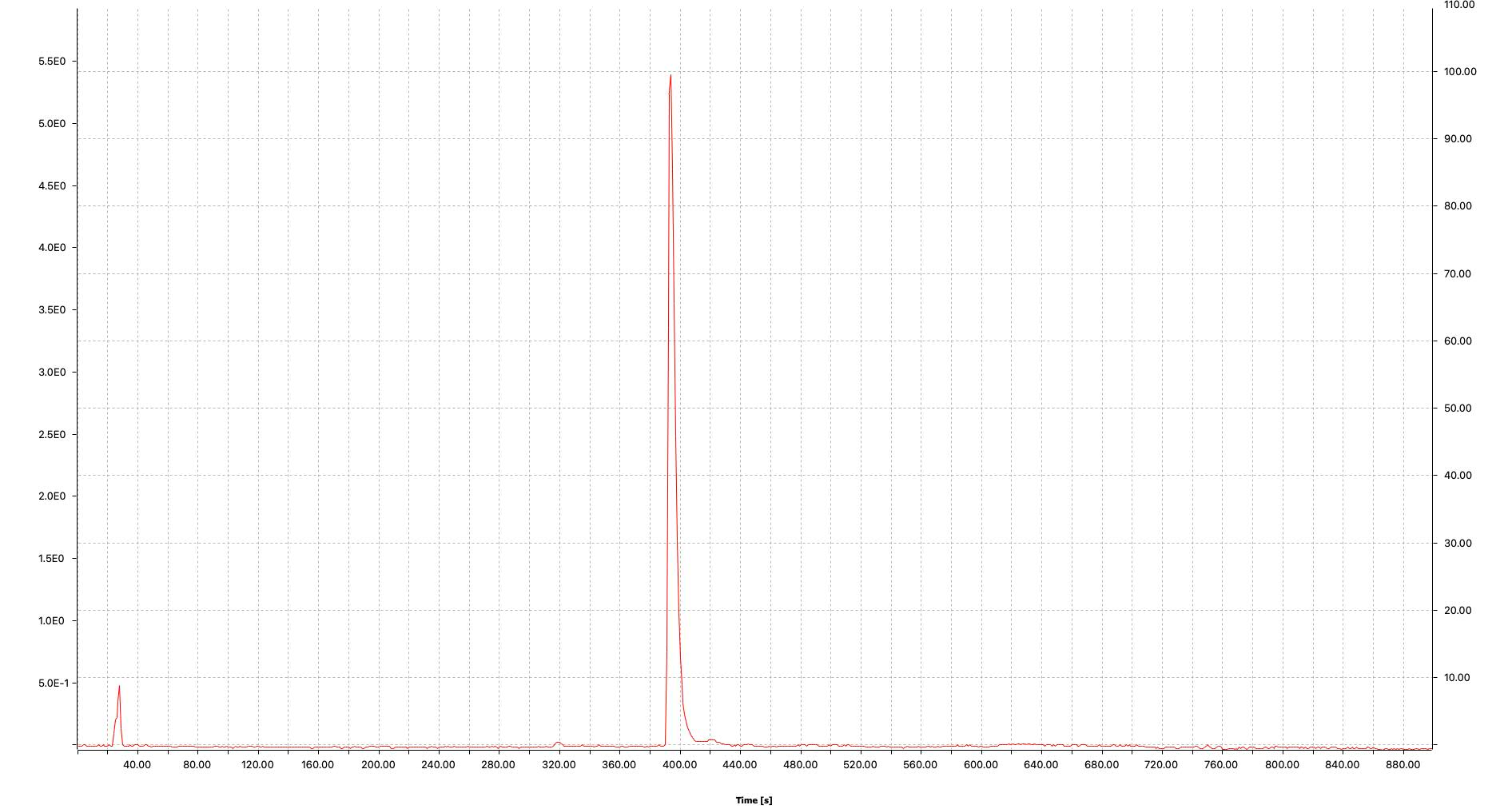


**Fig. S17: HPLC-UV chromatogram of RO0627603**


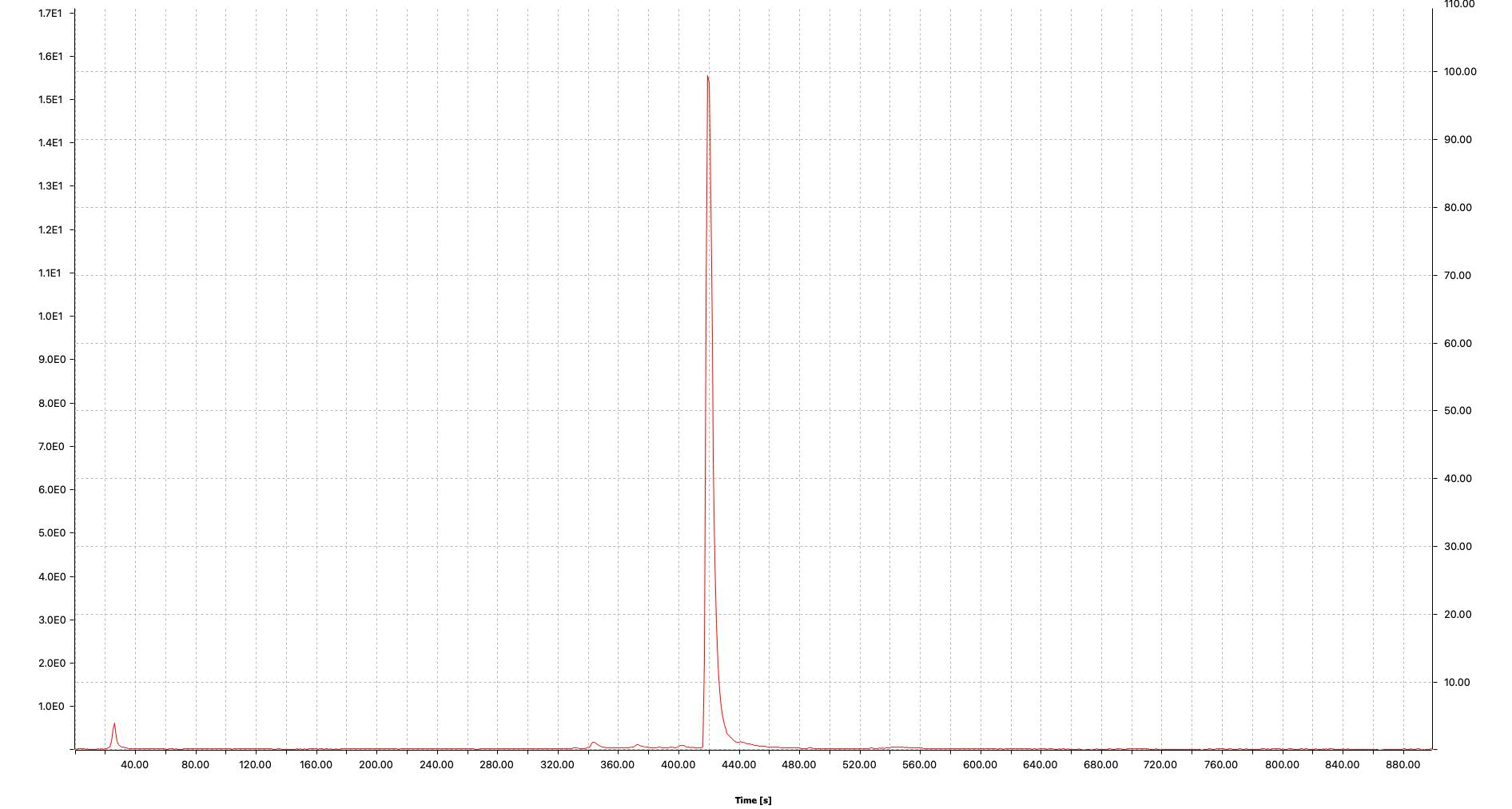


**Fig. S18: HPLC-UV chromatogram of RO0616411**


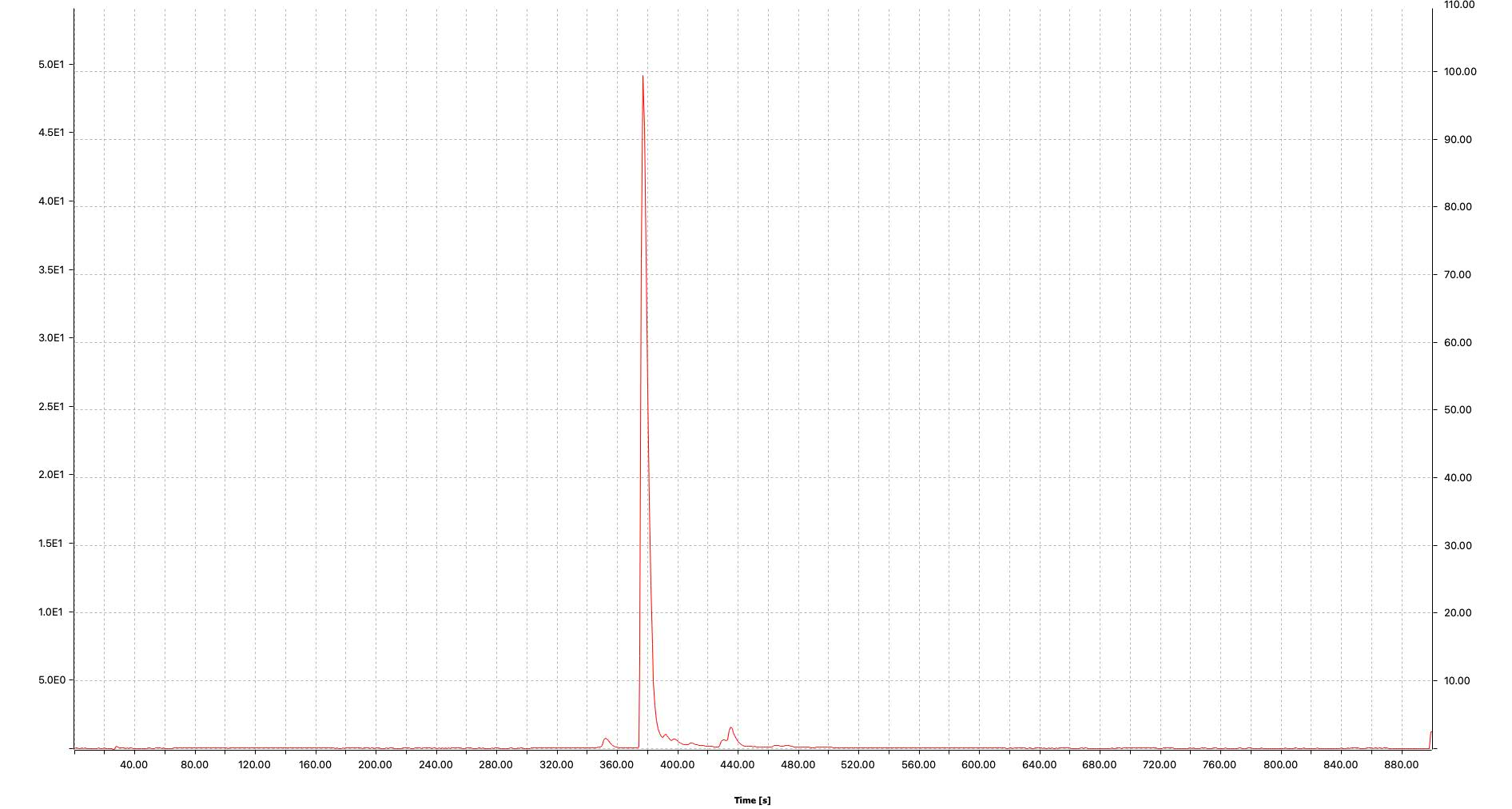


**Fig. S19: HPLC-UV chromatogram of RO0620573**


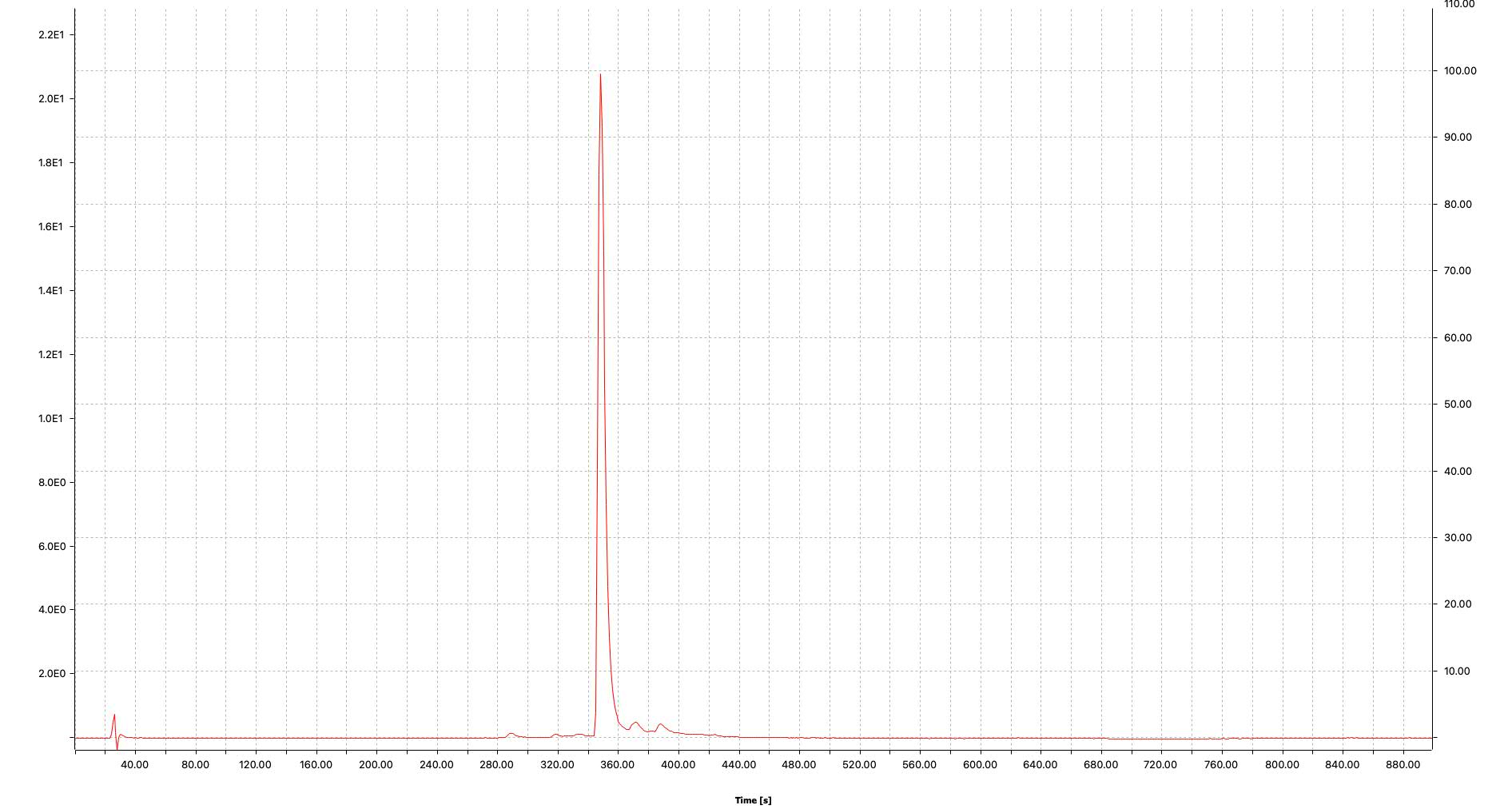


**Fig. S20: HPLC-UV chromatogram of RO0199733**


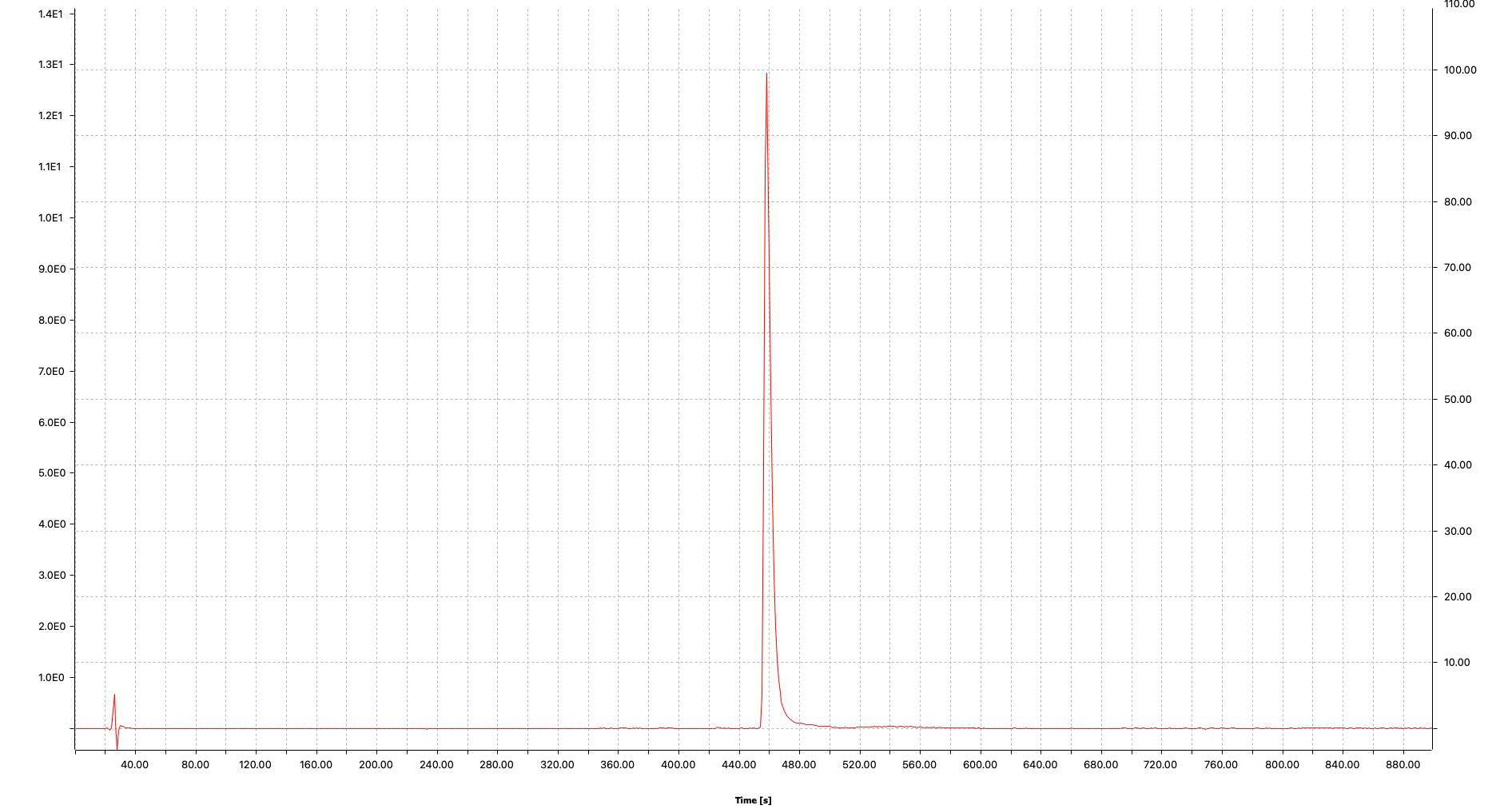


**Fig. S21: HPLC-UV chromatogram of RO0620687**


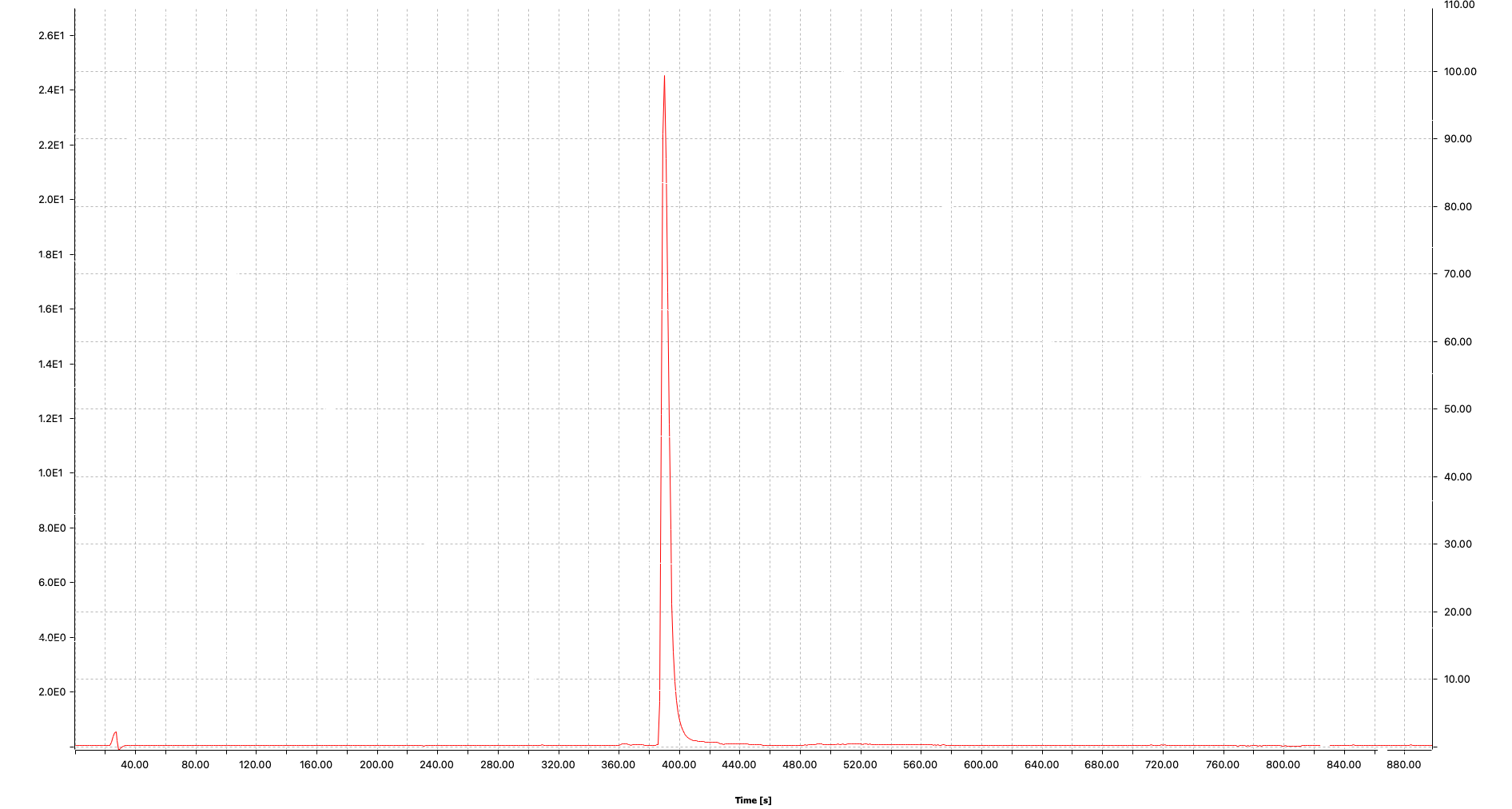


**Fig. S22: HPLC-UV chromatogram of RO0620808**


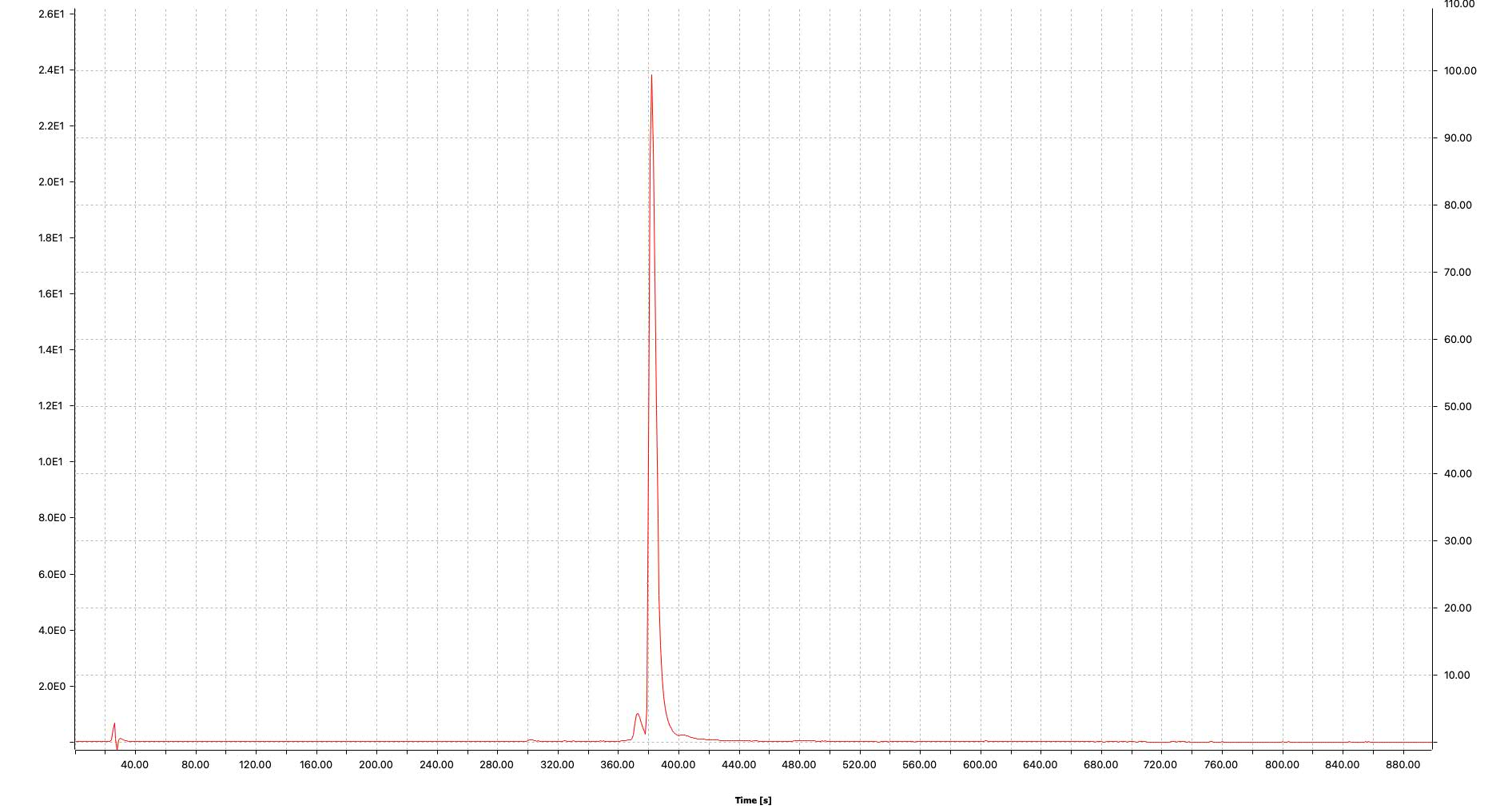


**Fig. S23: HPLC-UV chromatogram of RO0624122**


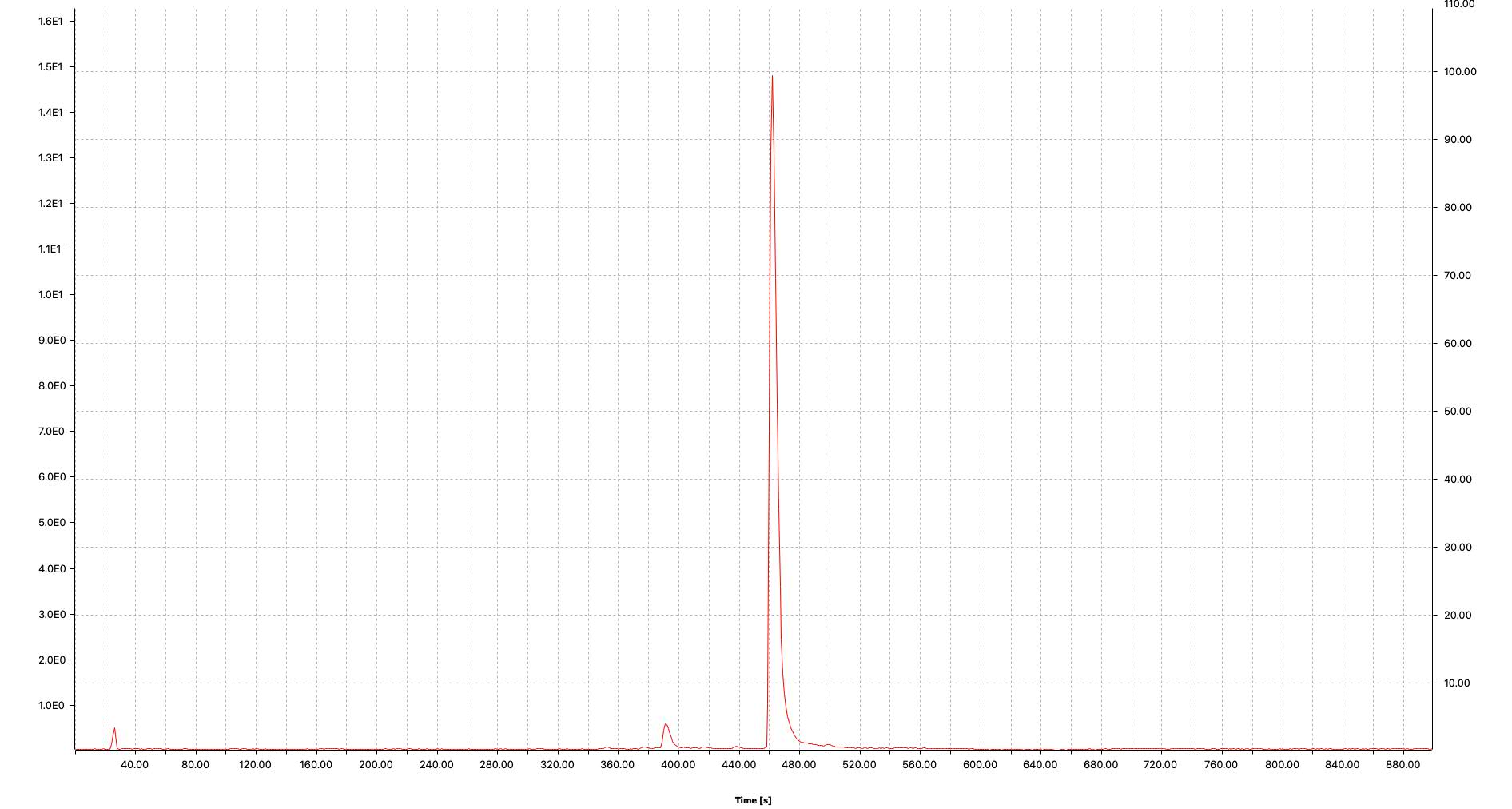


**Fig. S24: HPLC-UV chromatogram of RO0620411**


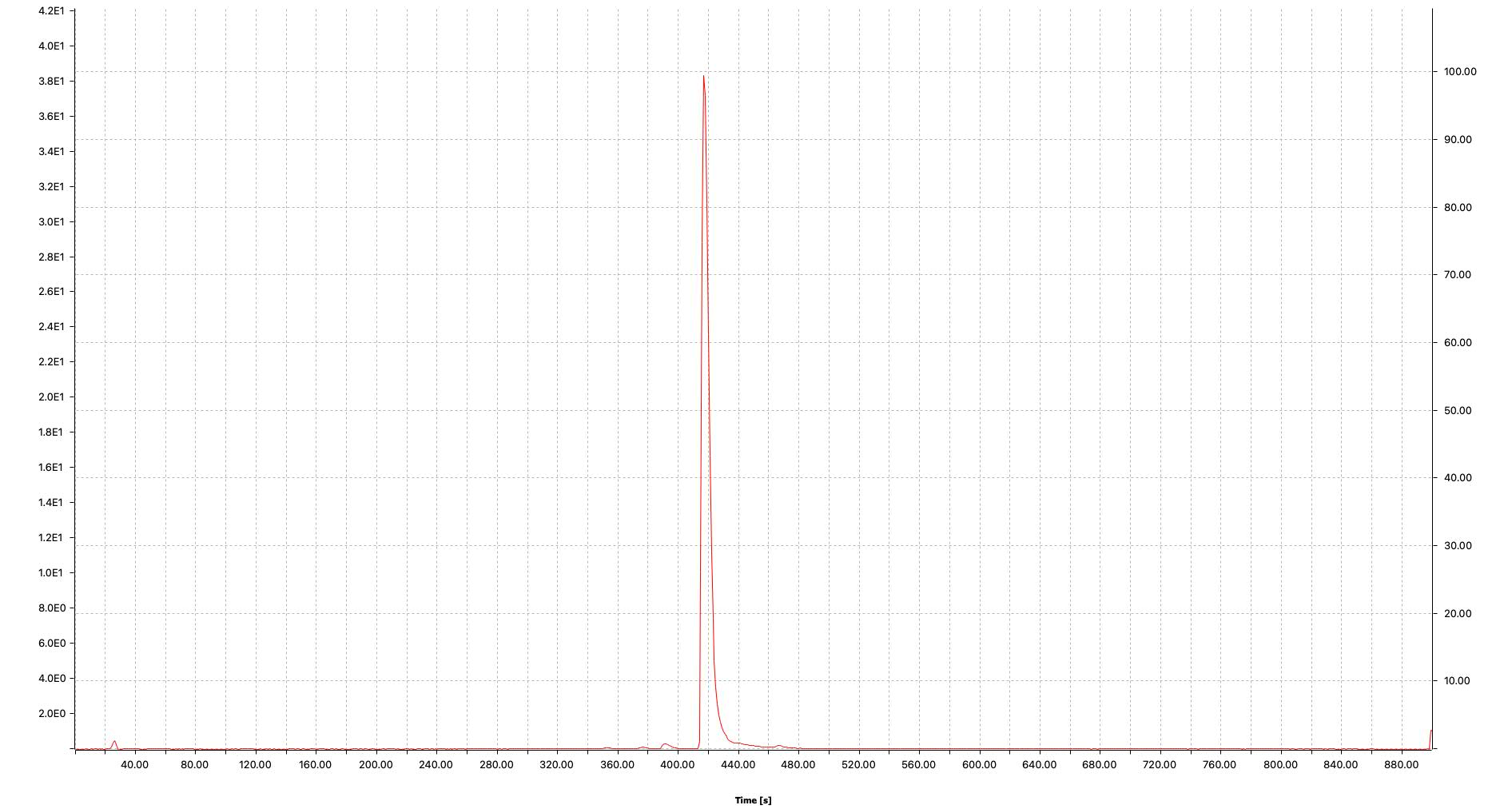


**Fig. S25: HPLC-UV chromatogram of RO0620410**


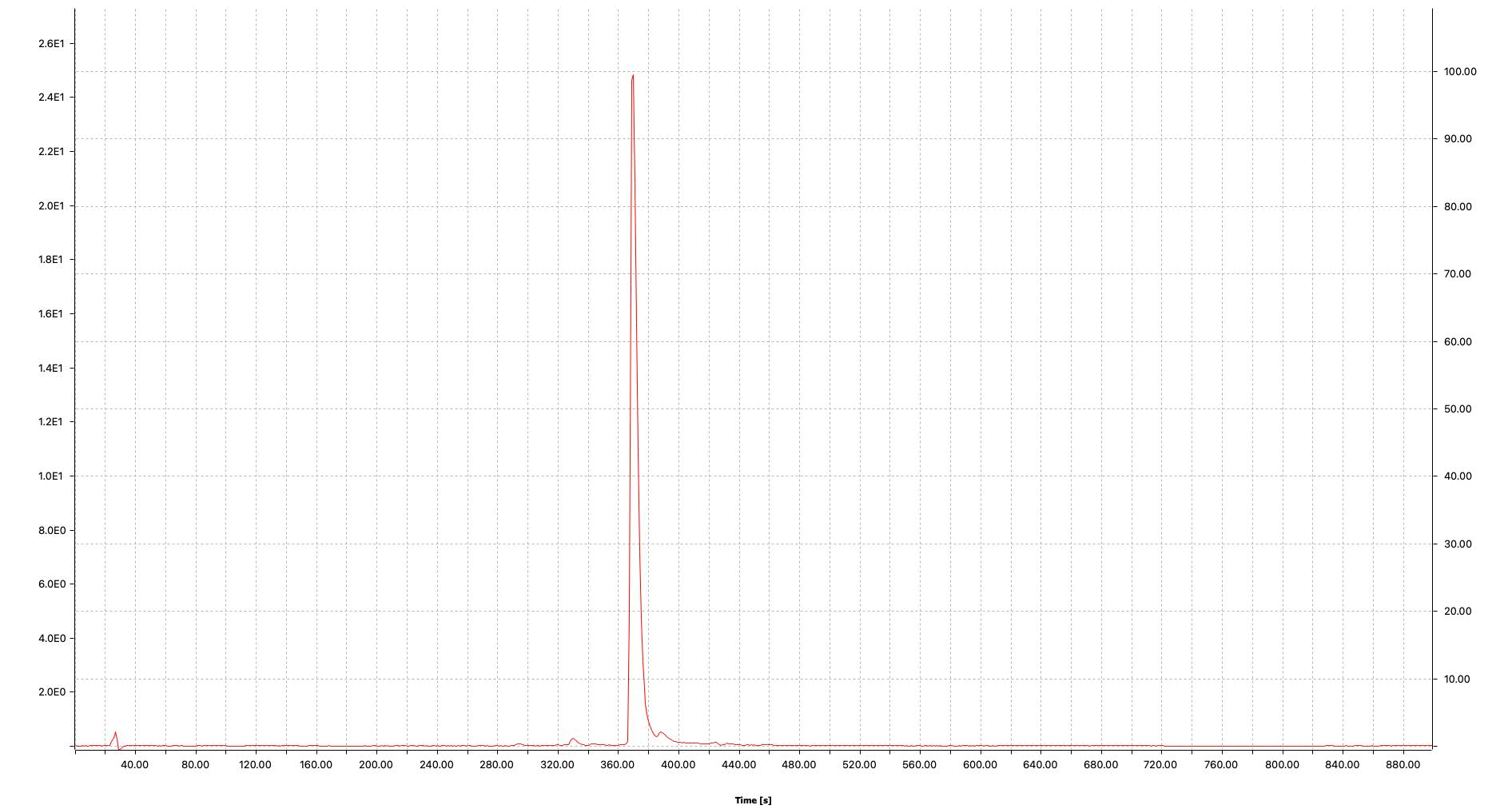


**Fig. S26: HPLC-UV chromatogram of RO0620570**


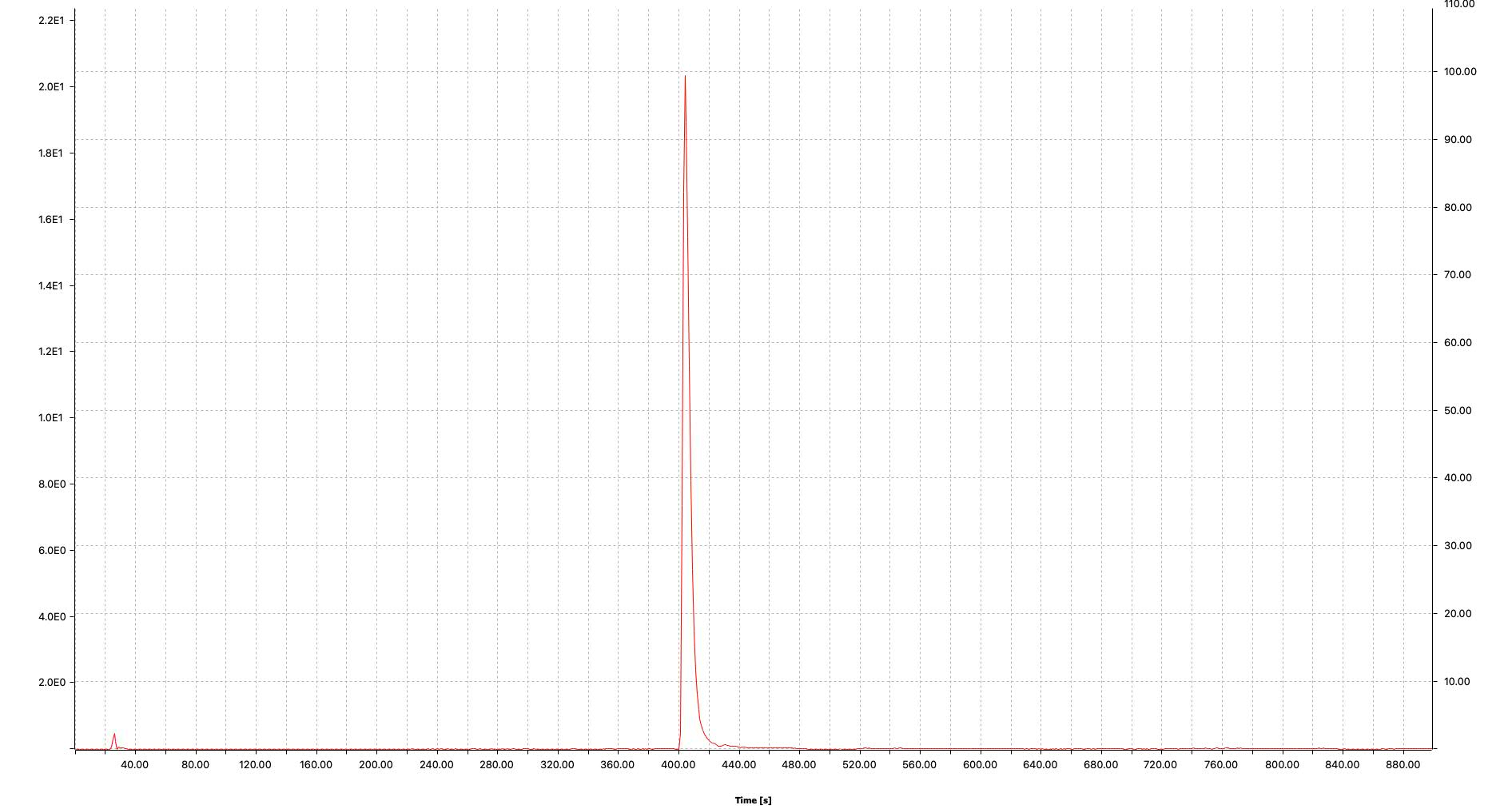


**Fig. S27: HPLC-UV chromatogram of RO0626783**


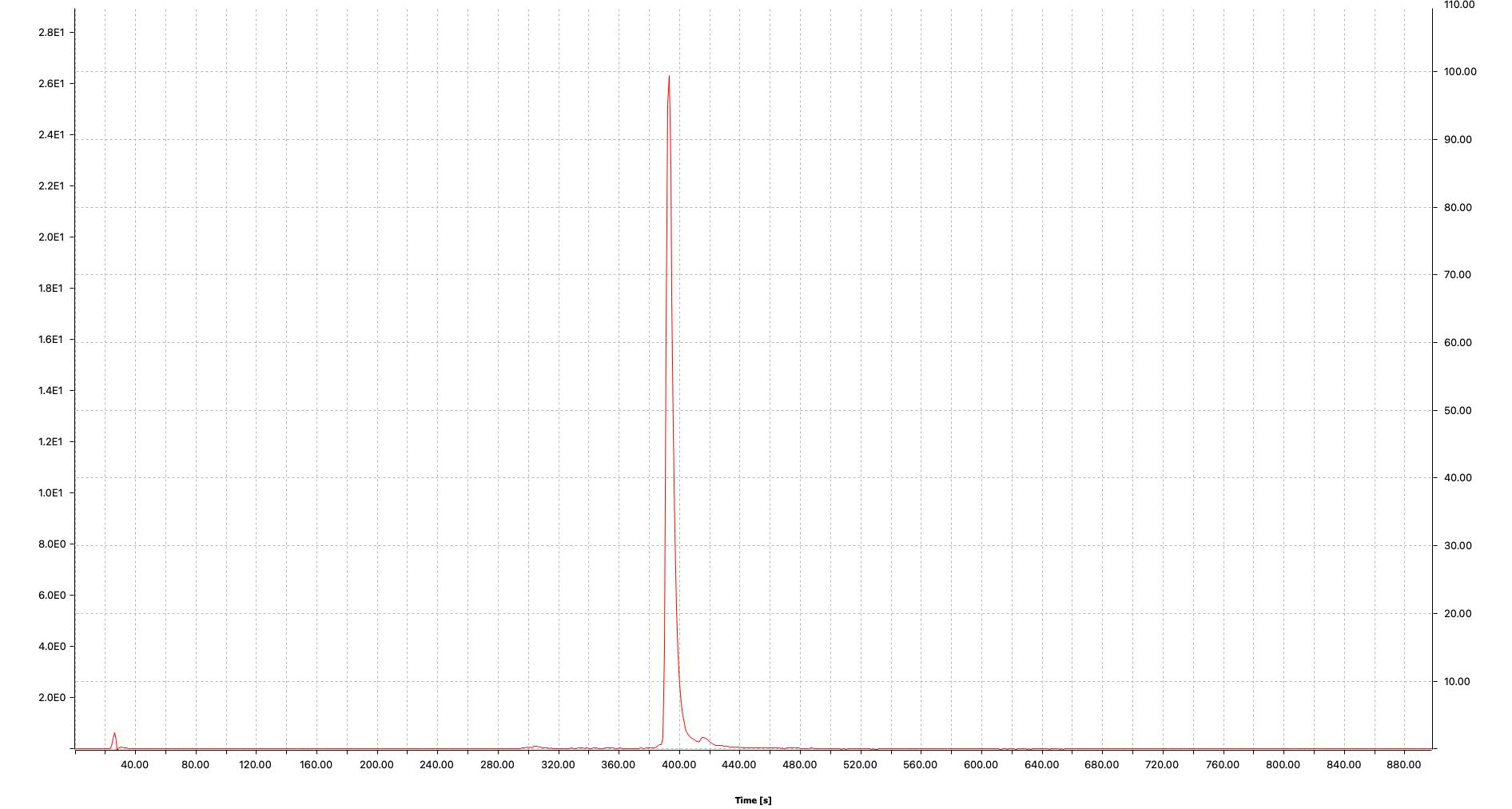


**Fig. S28: HPLC-UV chromatogram of RO0407028**


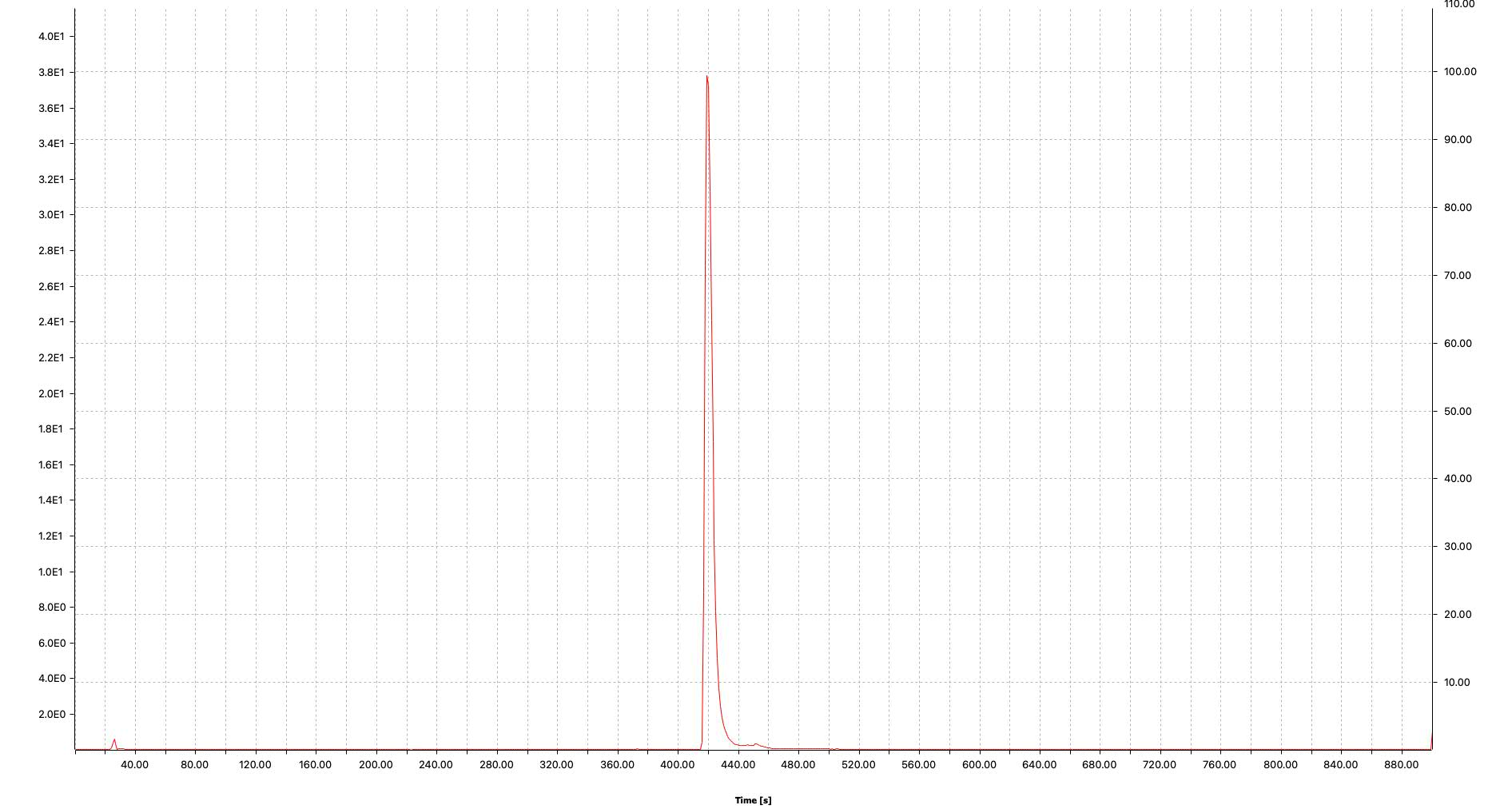


**Fig. S29: HPLC-UV chromatogram of RO0618764**


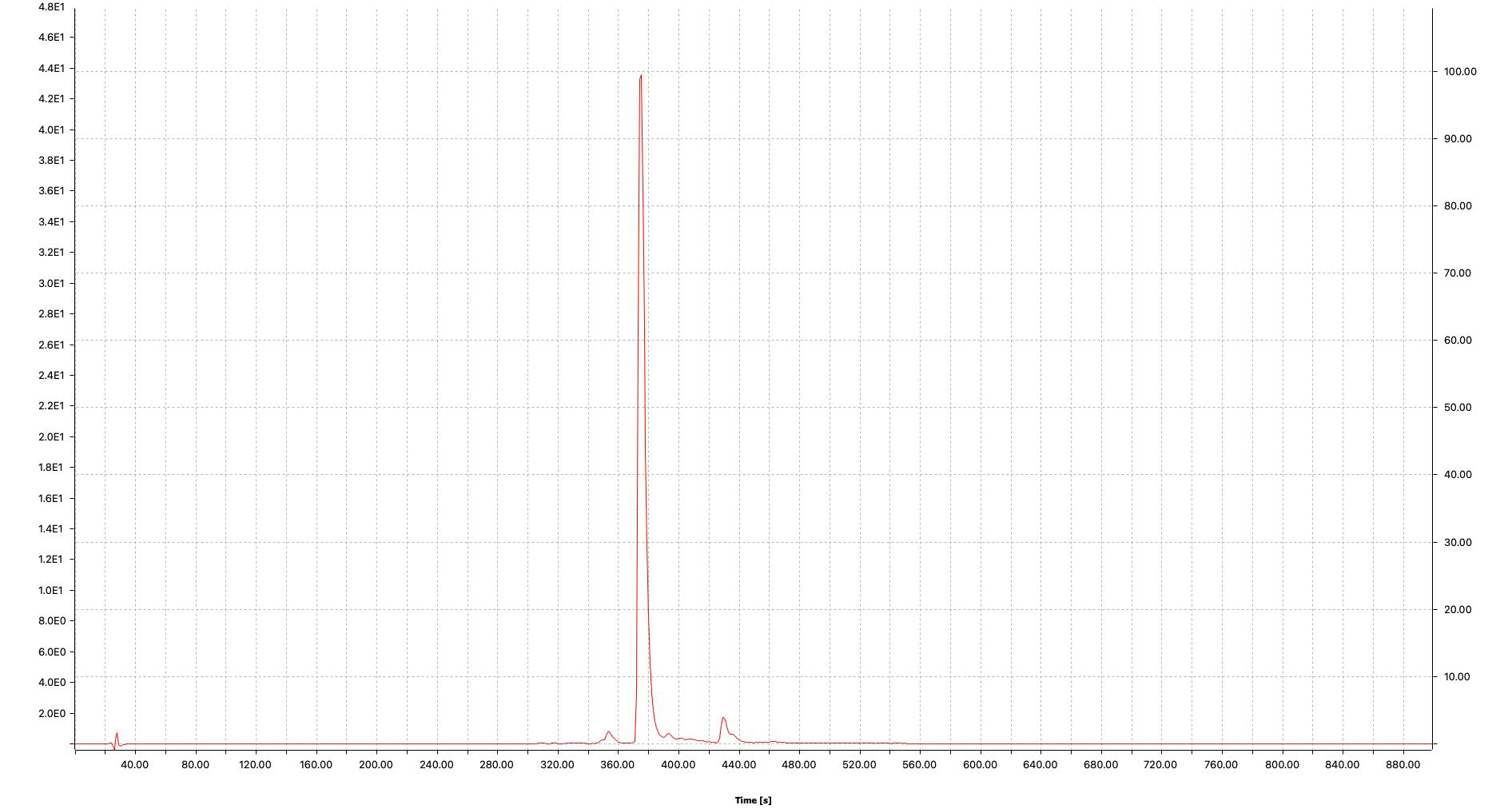


**Fig. S30: HPLC-UV chromatogram of RO0621239**


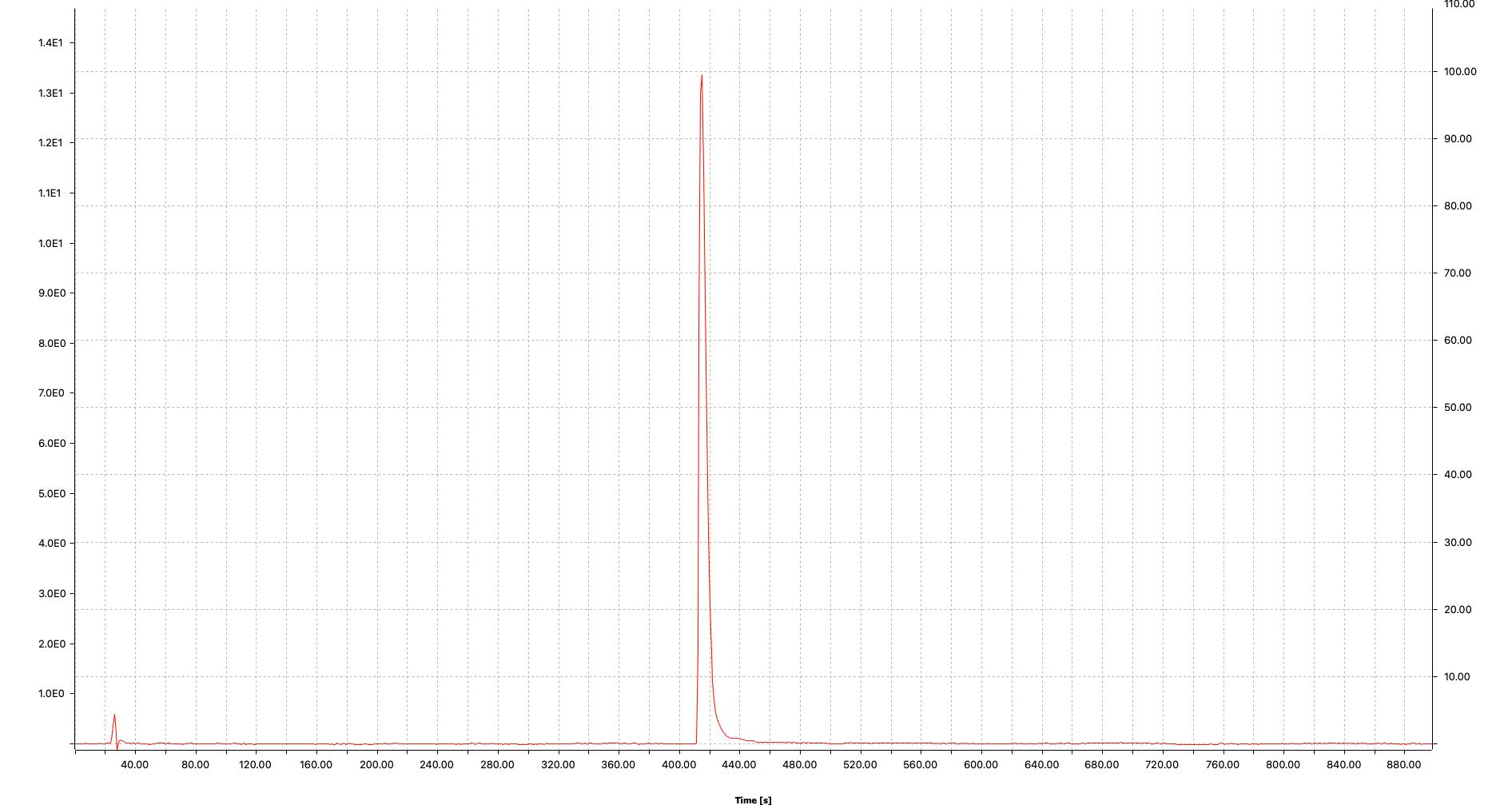


**Fig. S31: HPLC-UV chromatogram of RO0620810**


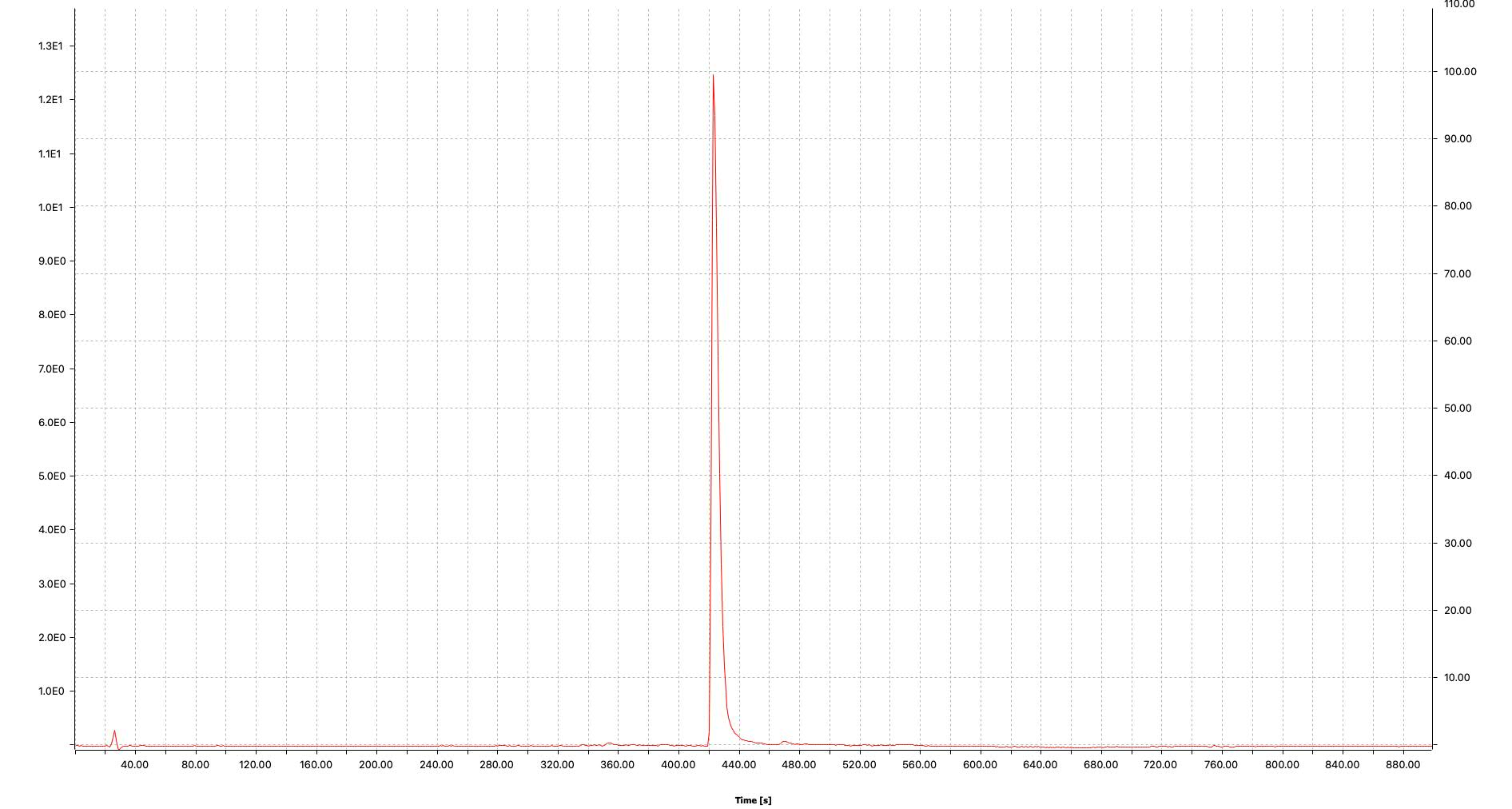


**Fig. S32: HPLC-UV chromatogram of RO0621109**


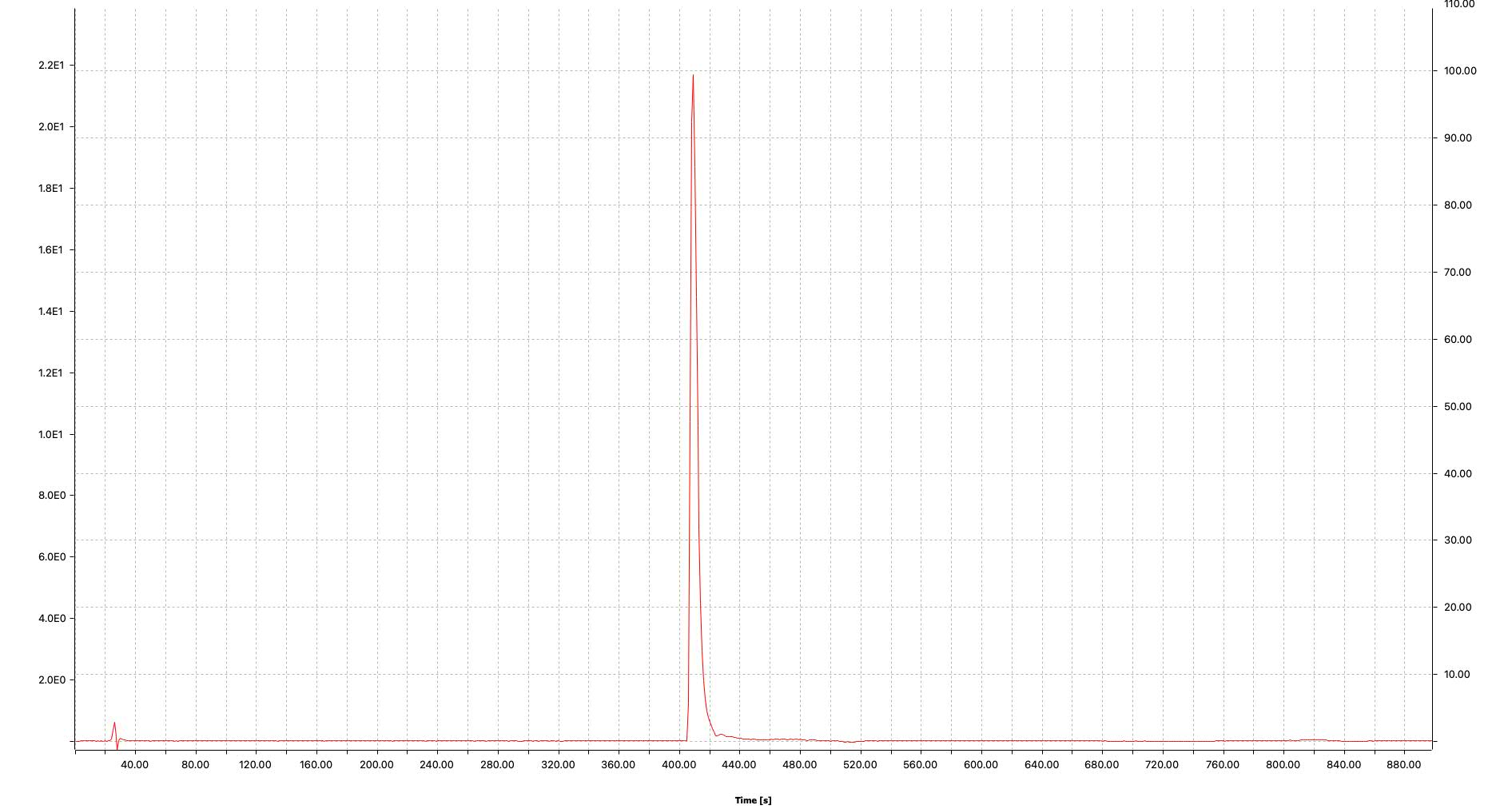


**Fig. S33: HPLC-UV chromatogram of RO0621116**


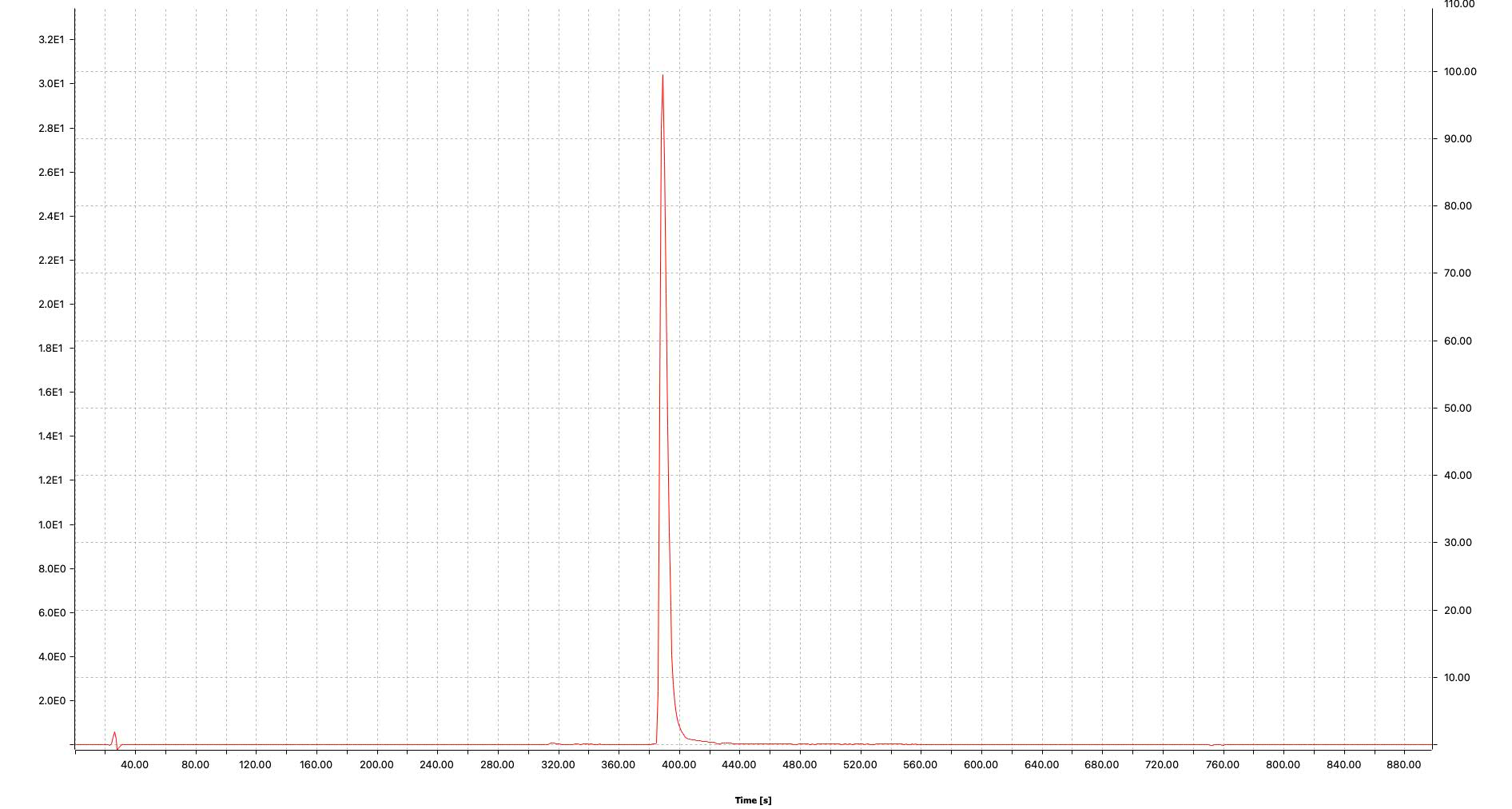


**Fig. S34: HPLC-UV chromatogram of RO0621006**


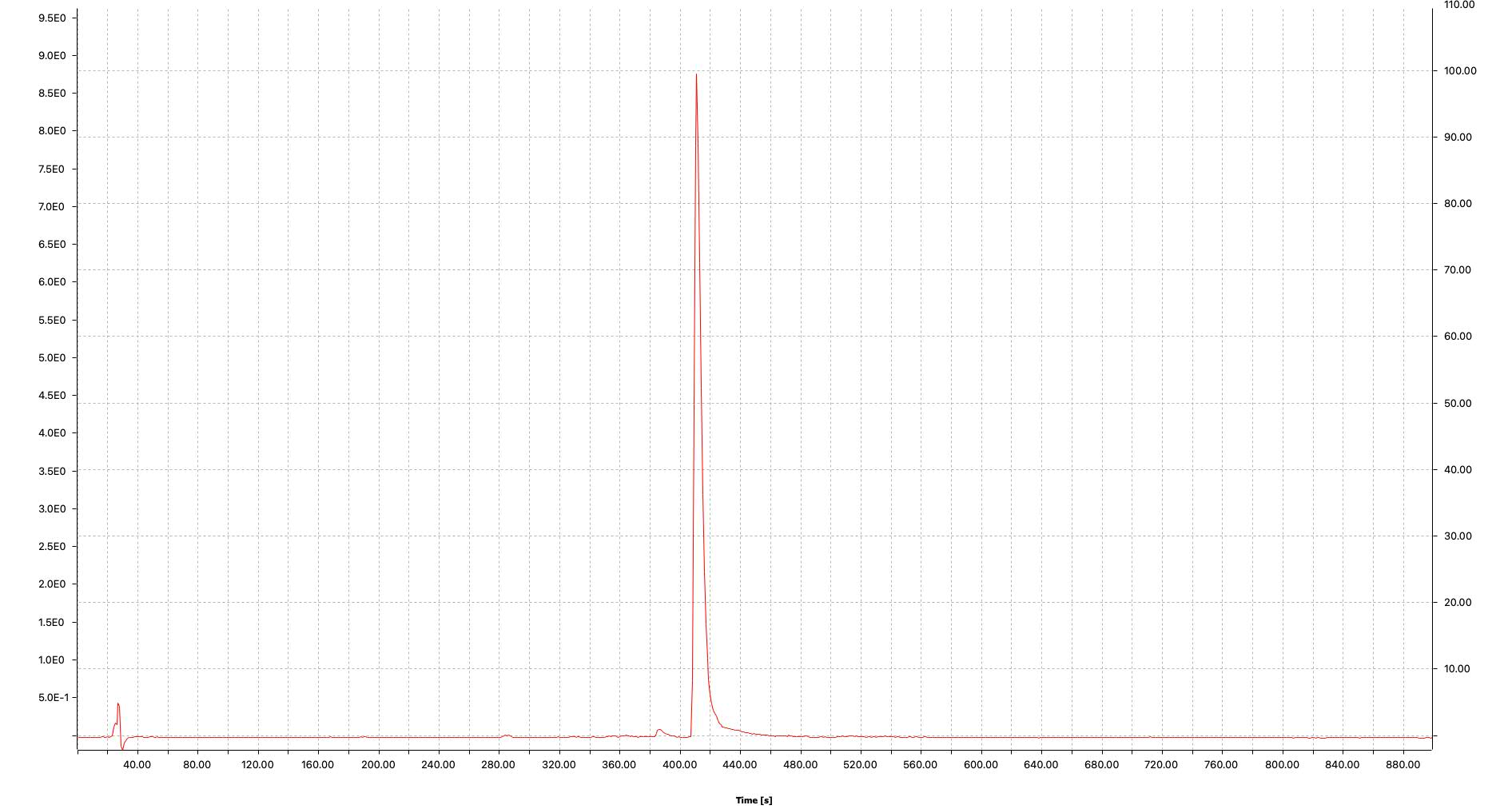


**Fig. S35: HPLC-UV chromatogram of RO0619291**


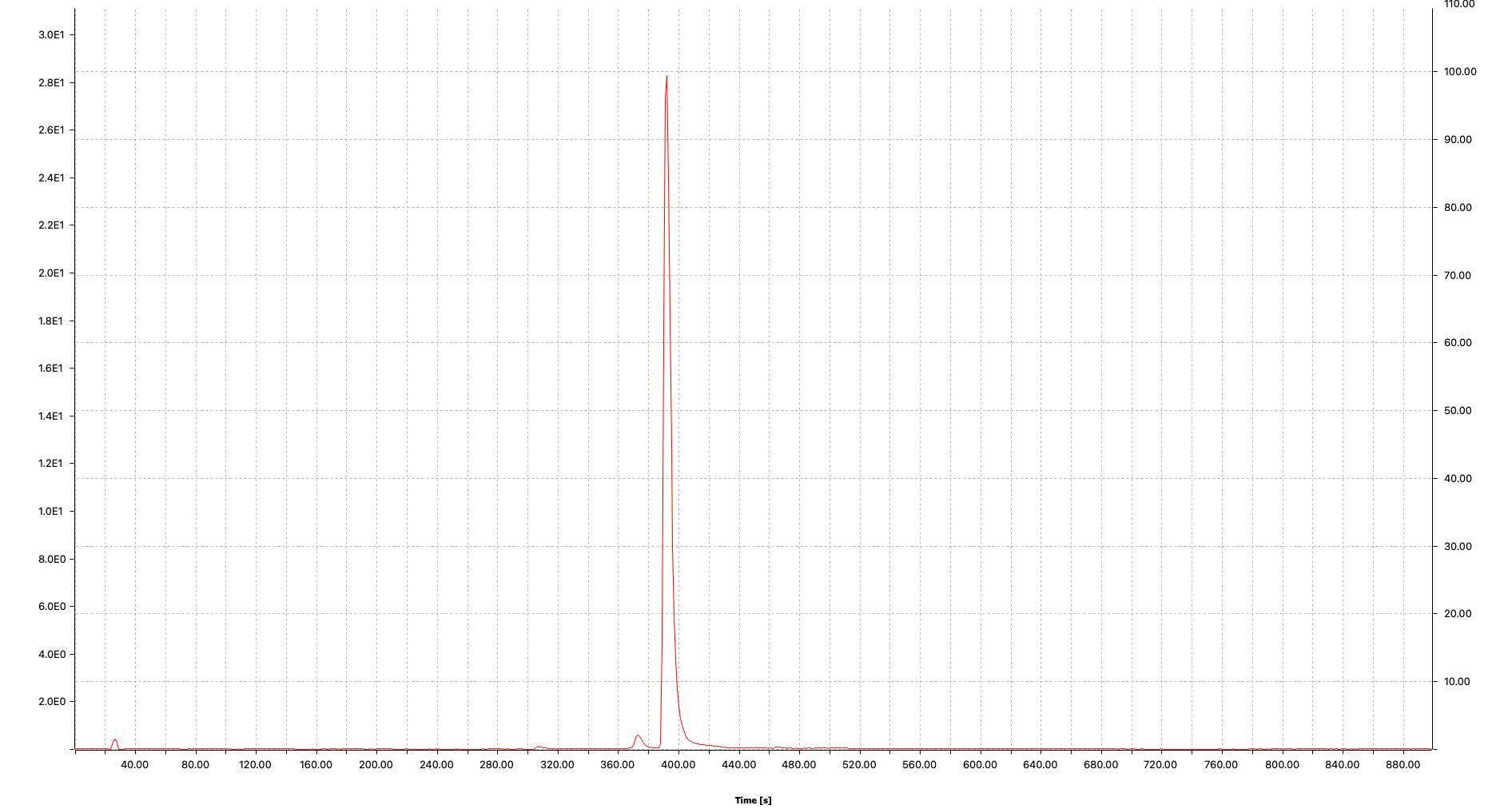


**Fig. S36: HPLC-UV chromatogram of RO0619265**


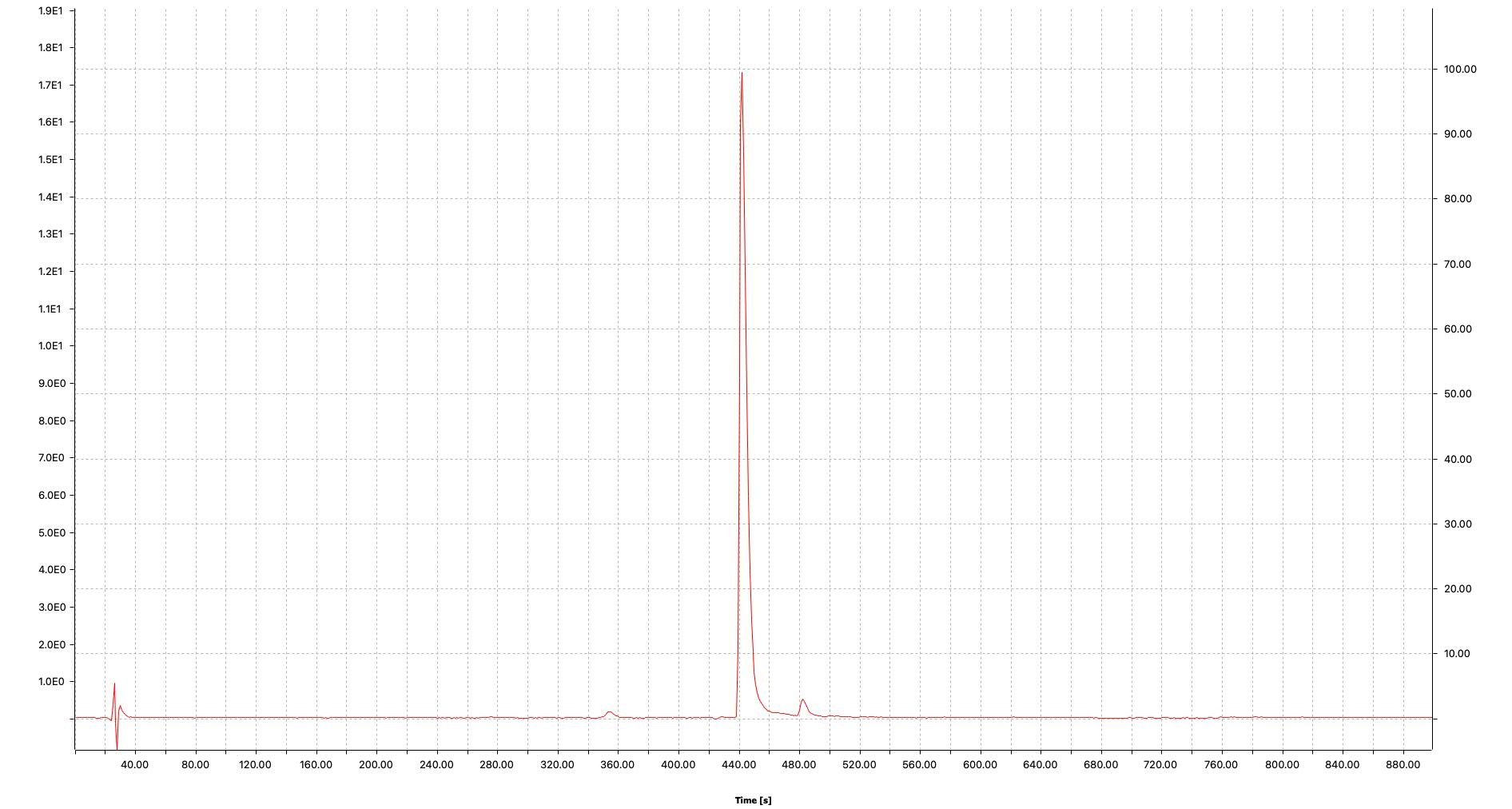


**Fig. S37: HPLC-UV chromatogram of RO0626778**


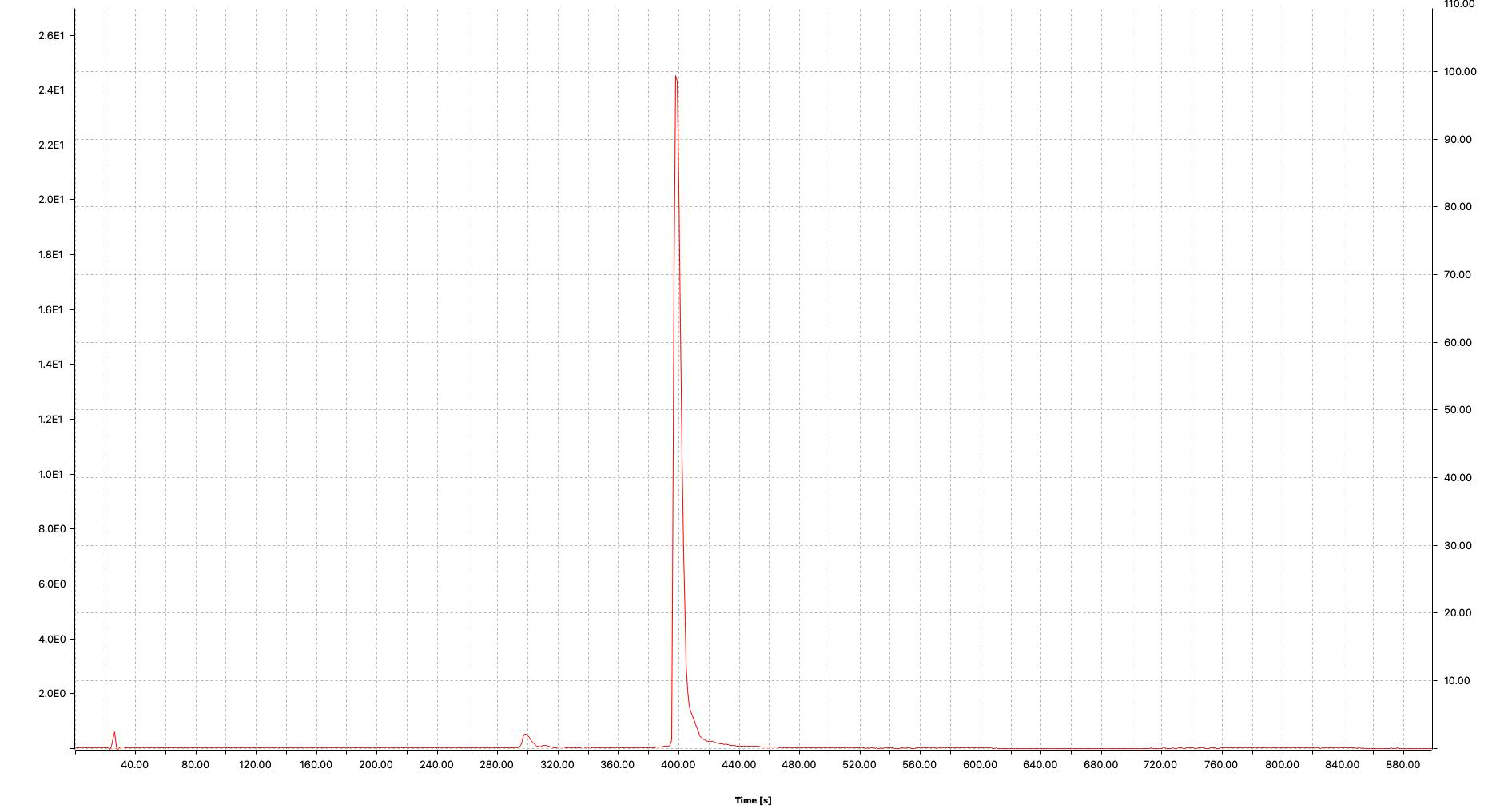


**Fig. S38: HPLC-UV chromatogram of RO0413301**


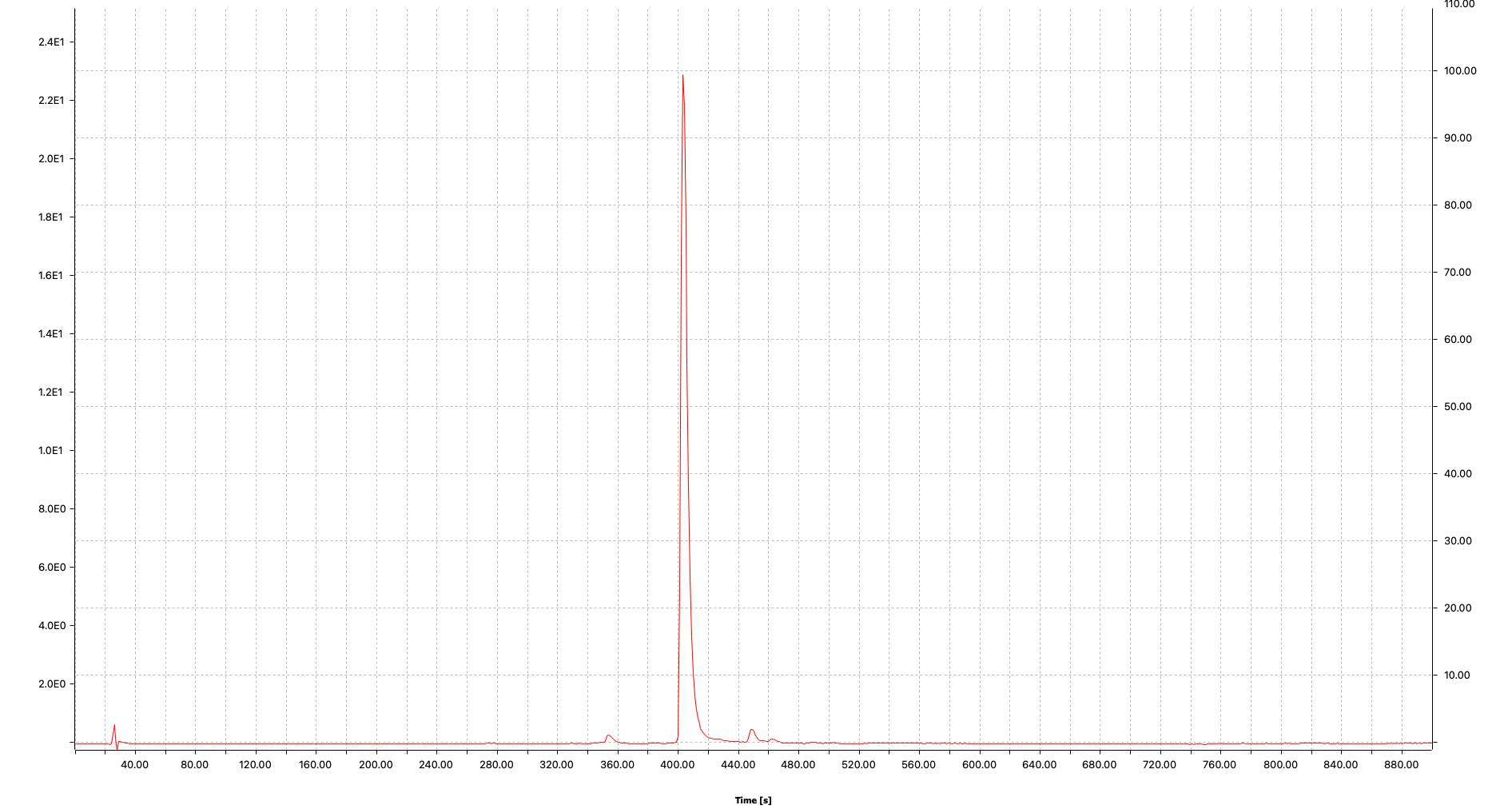


**Fig. S39: HPLC-UV chromatogram of RO0622624**


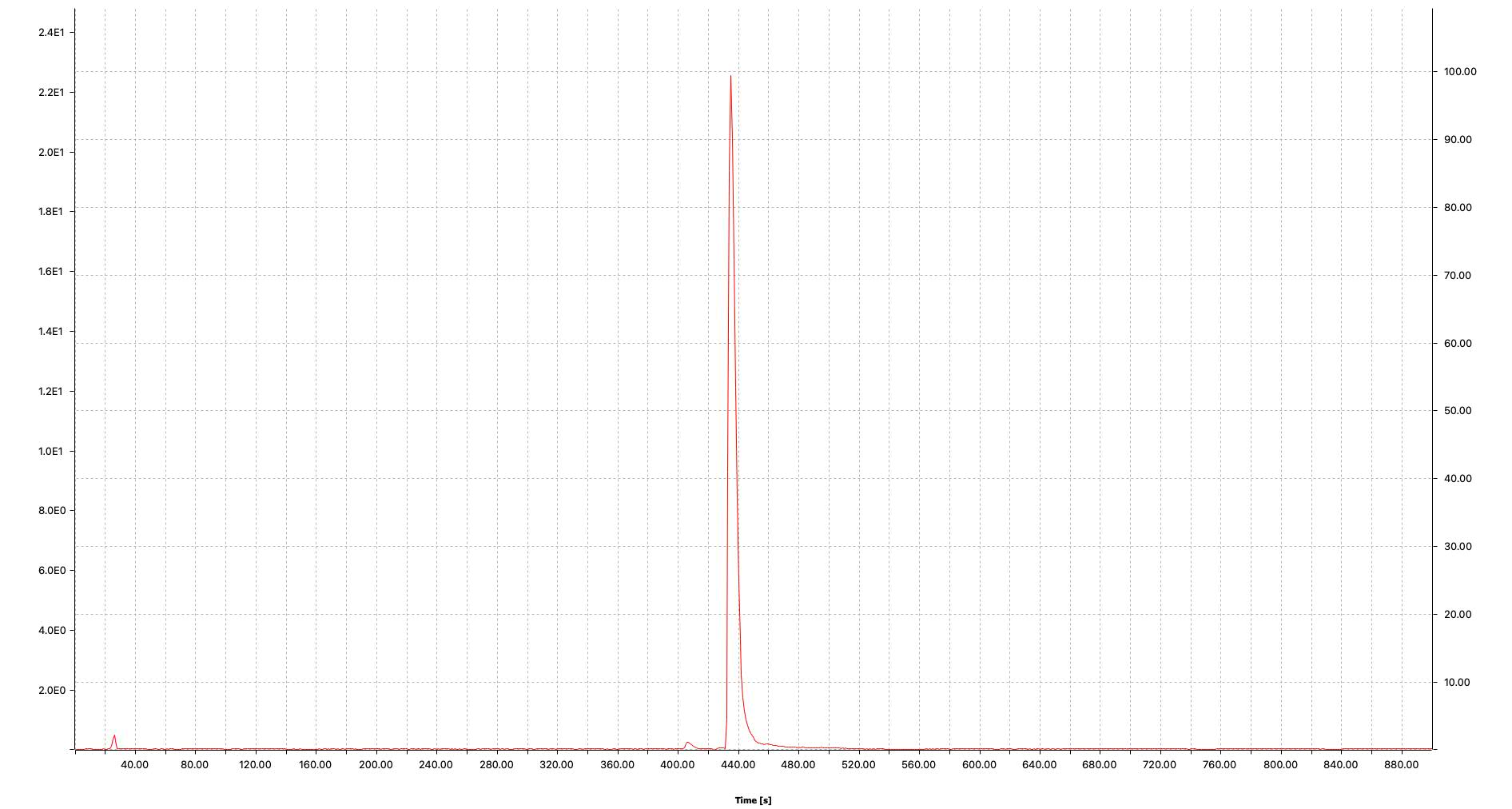


**Fig. S40: HPLC-UV chromatogram of RO0615928**


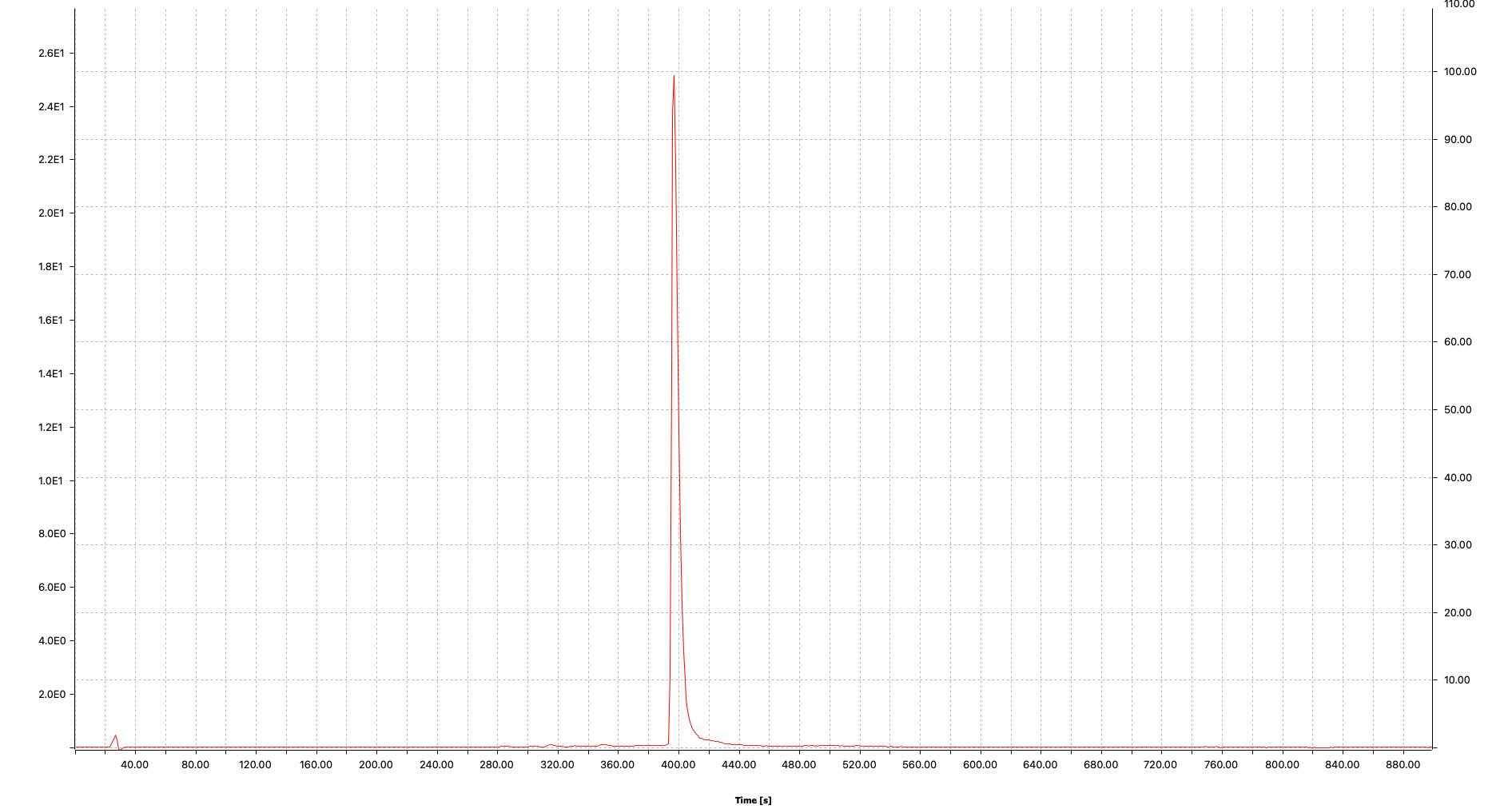


**Fig. S41: HPLC-UV chromatogram of RO0621161**


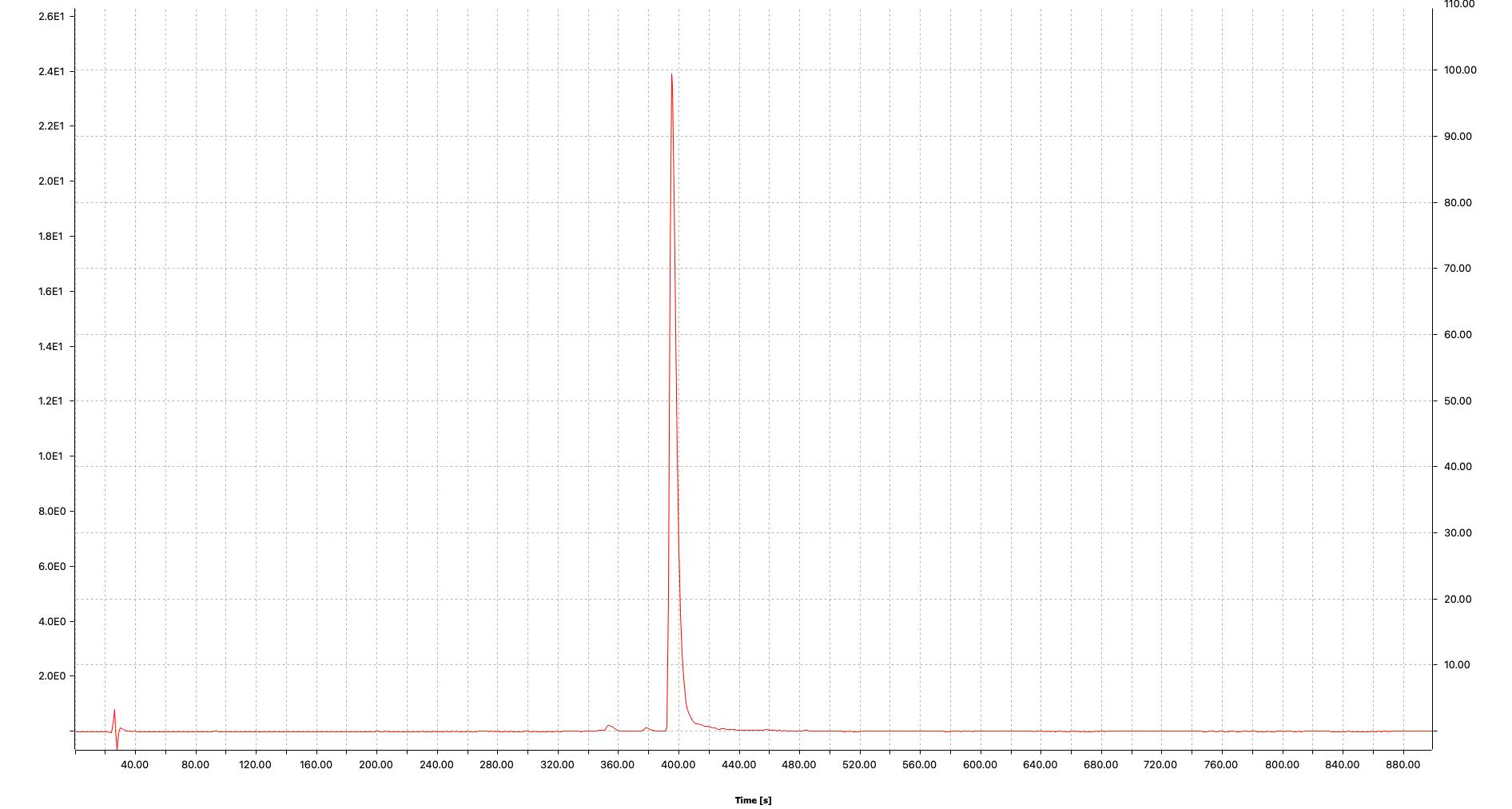


**Fig. S42: HPLC-UV chromatogram of RO0626786**


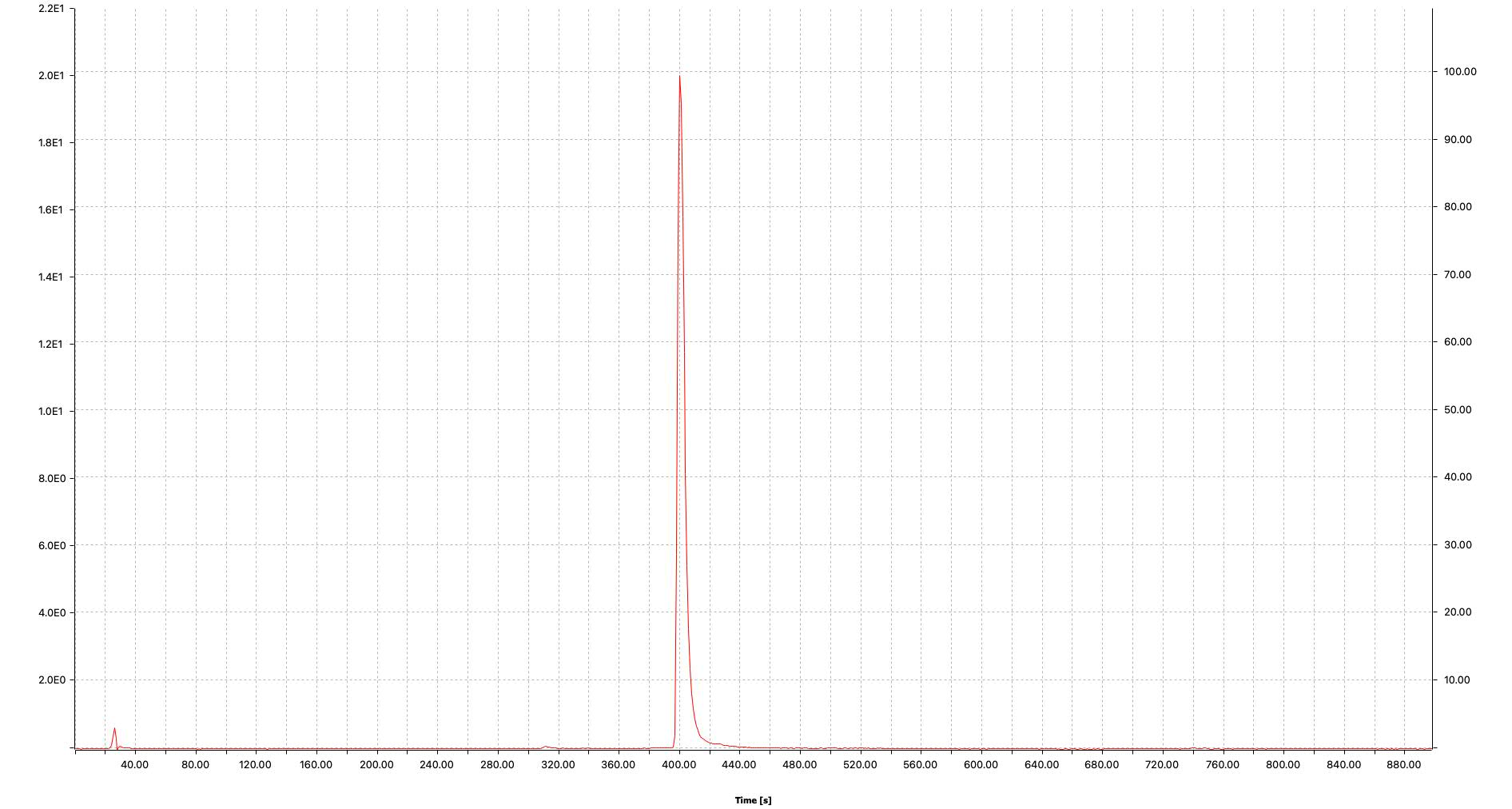


**Fig. S43: HPLC-UV chromatogram of RO0412734**


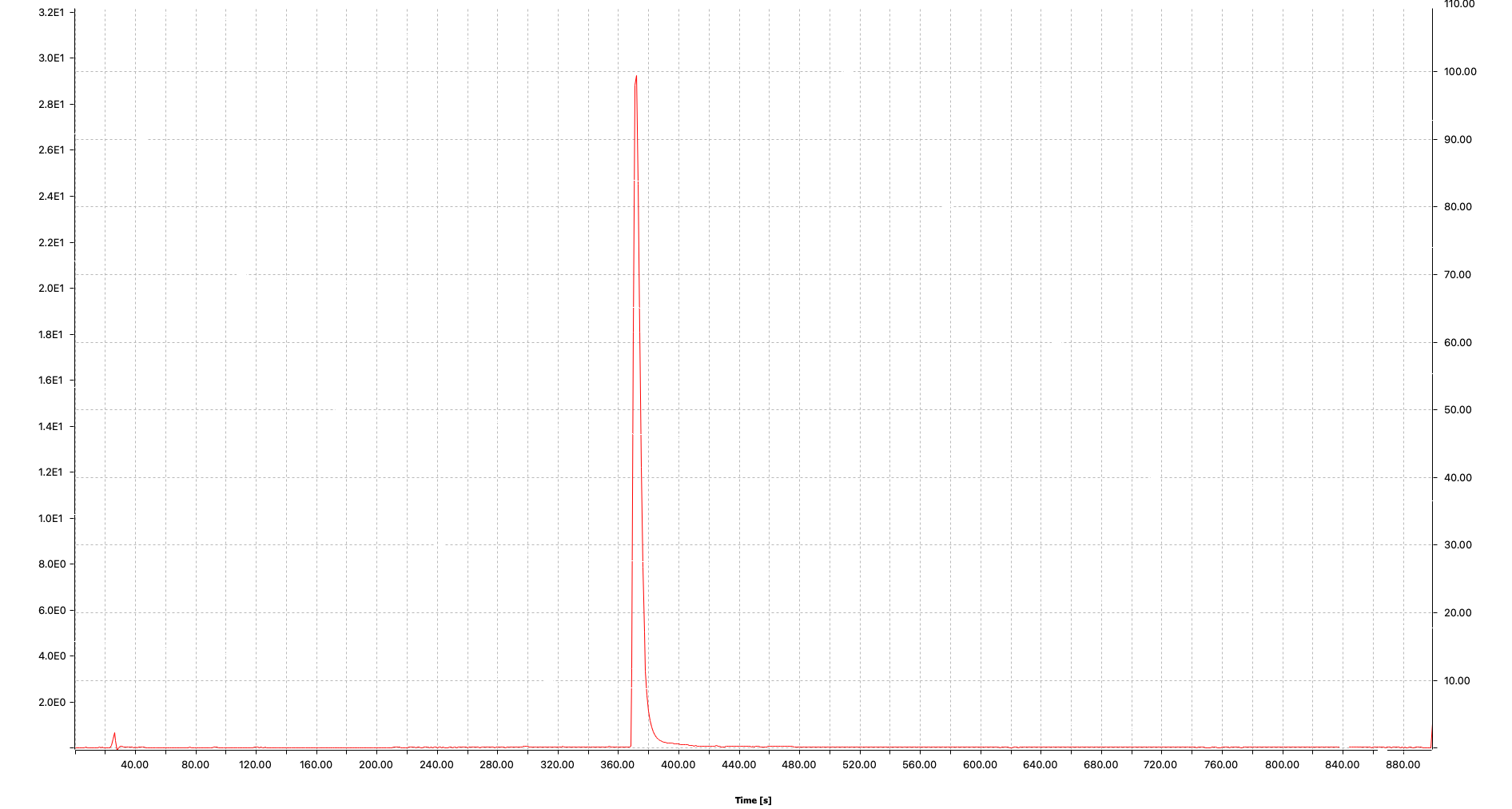


**Fig. S44: HPLC-UV chromatogram of RO0621005**


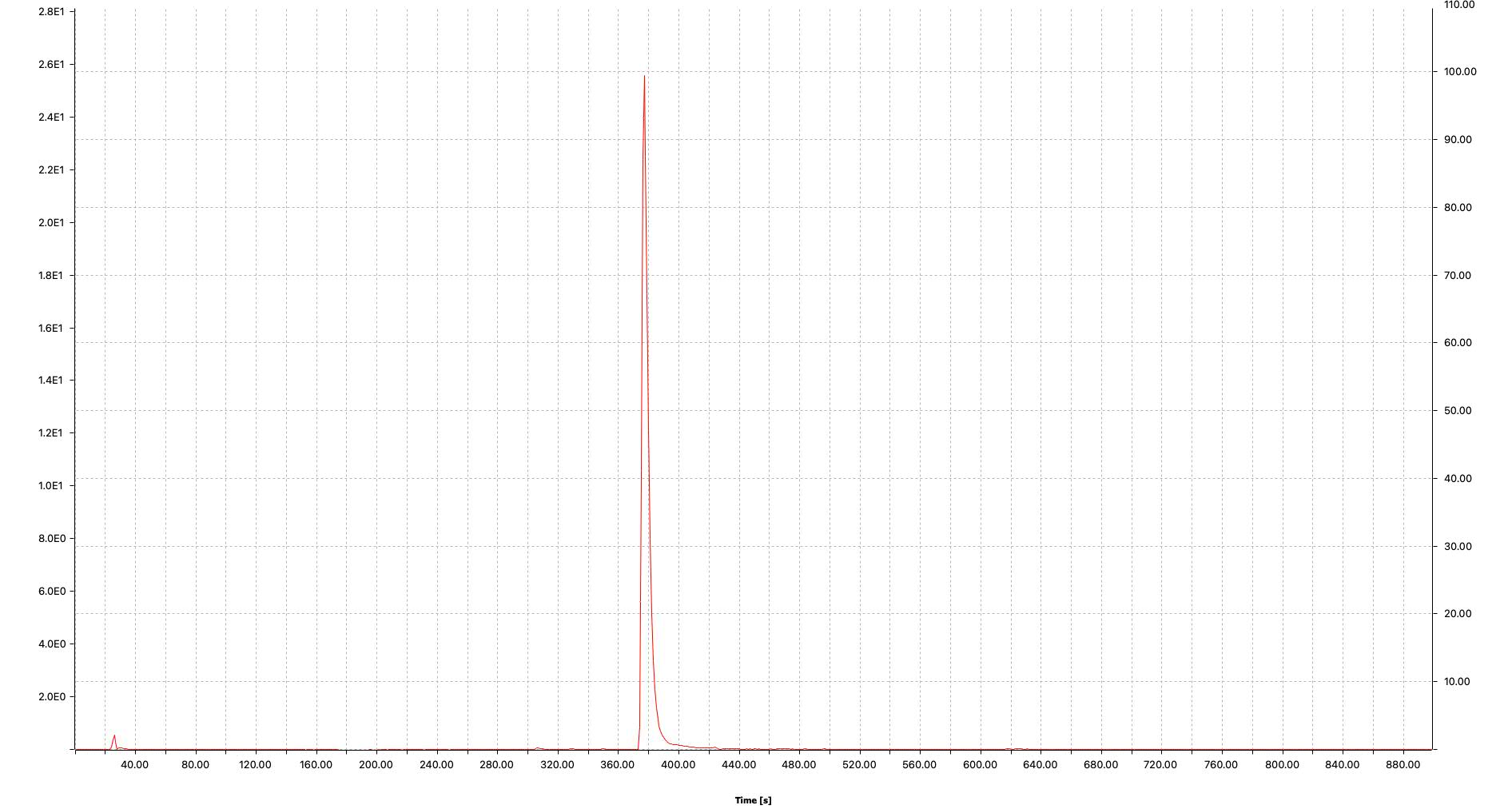


**Fig. S45: HPLC-UV chromatogram of RO0621115**


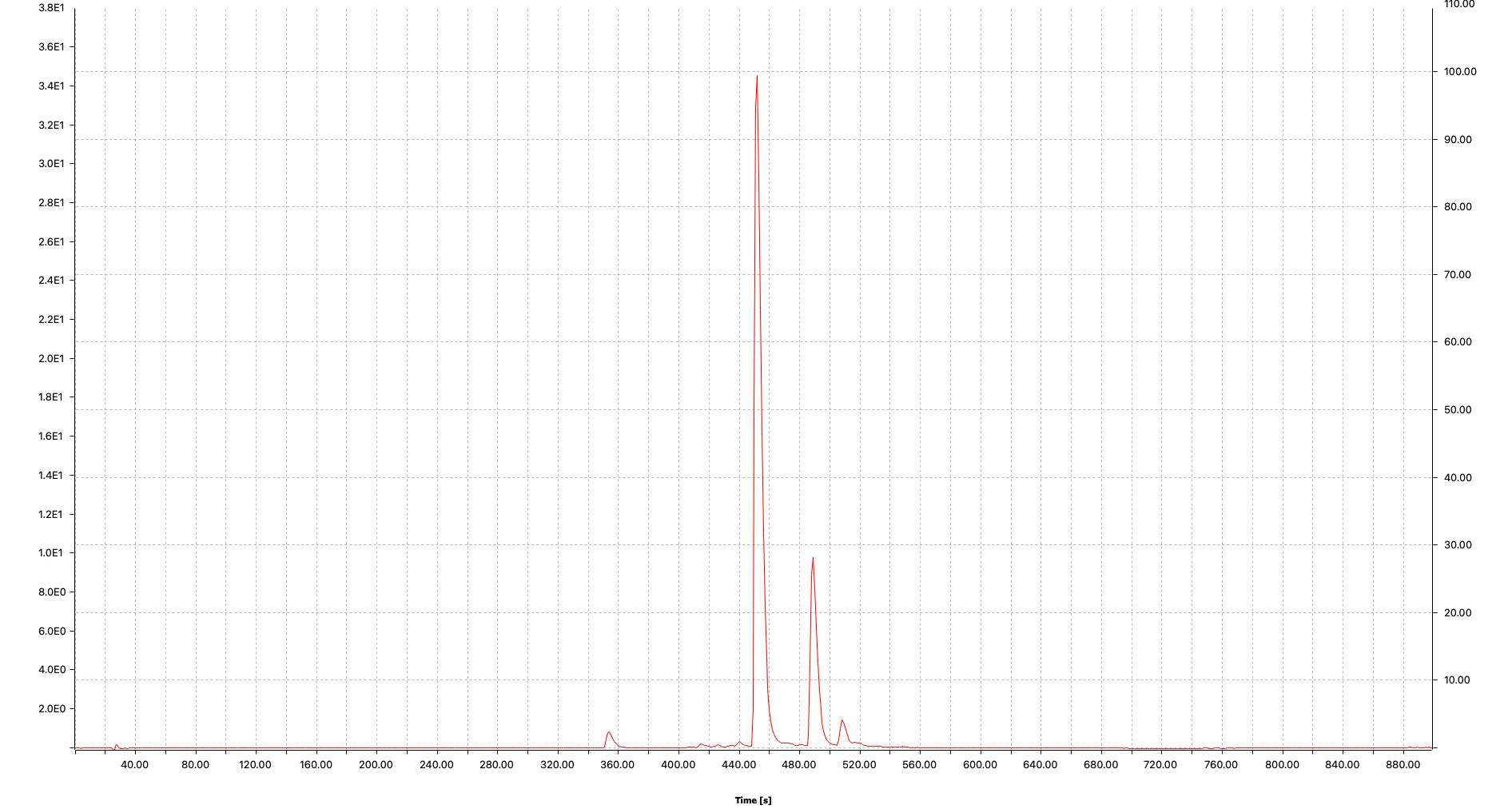


**Fig. S46: HPLC-UV chromatogram of RO0626777**


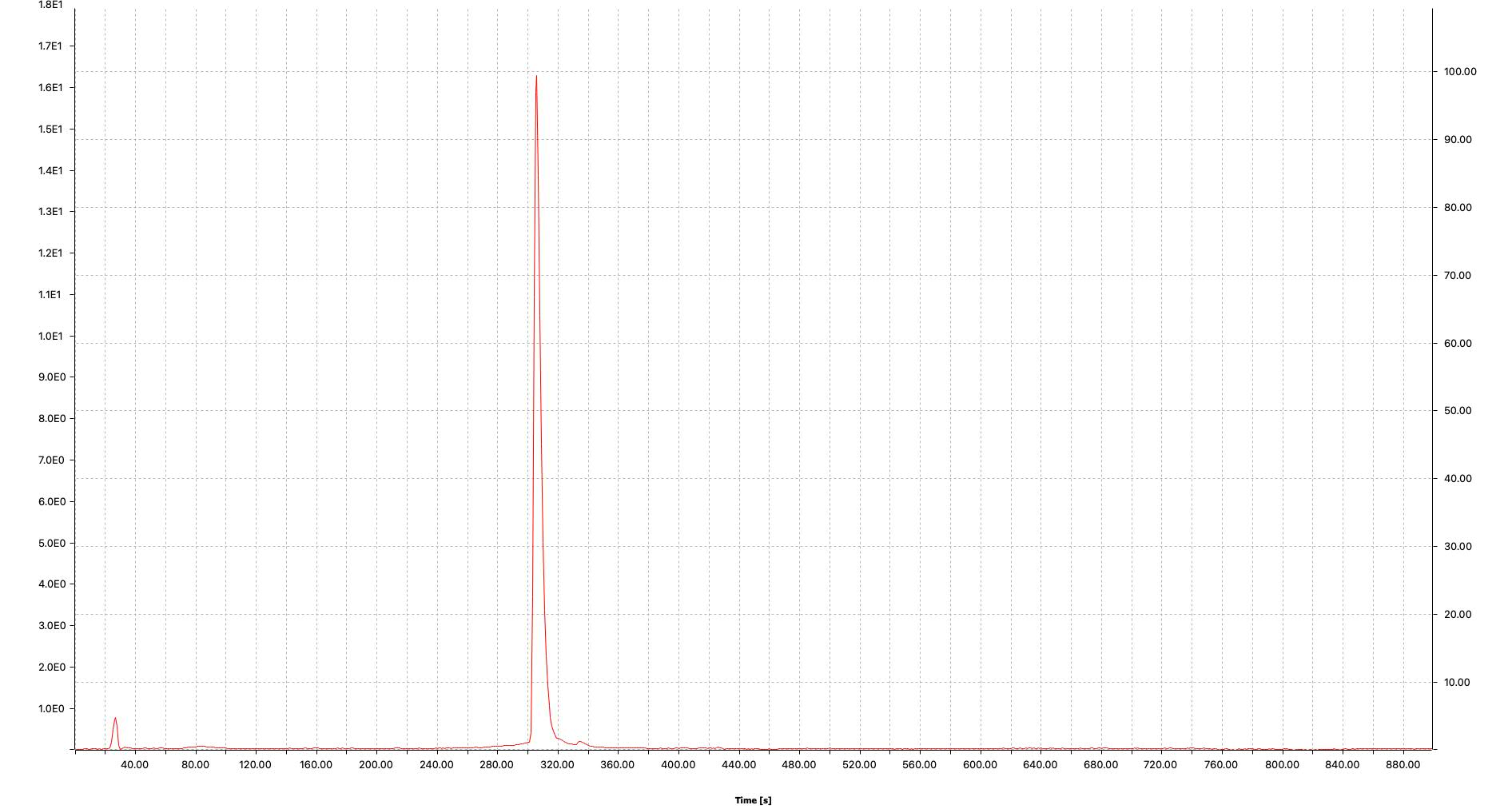


**Fig. S47: HPLC-UV chromatogram of RO0403499**


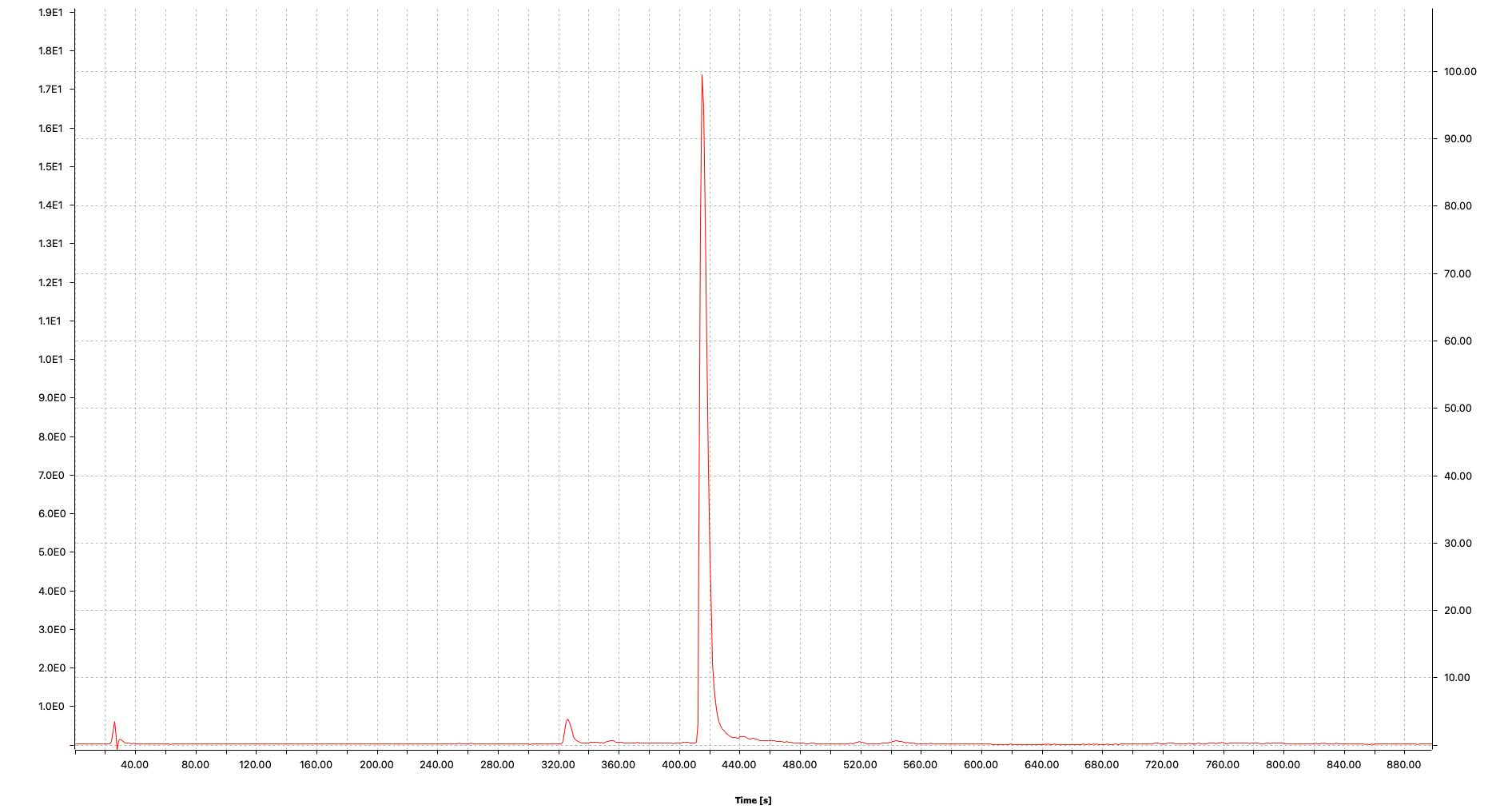


**Fig. S48: HPLC-UV chromatogram of RO0622671**


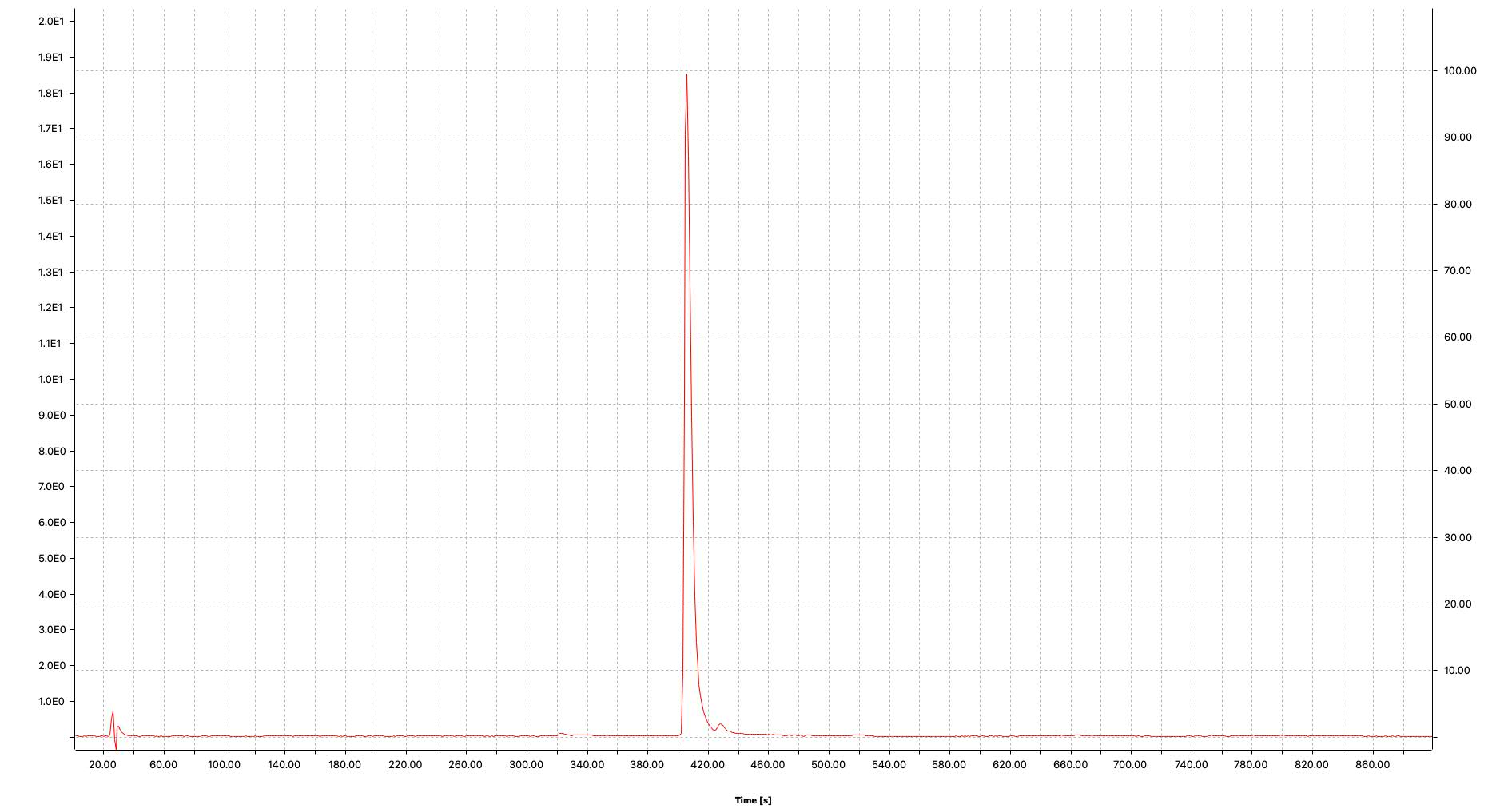


**Fig. S49: HPLC-UV chromatogram of RO0413162**


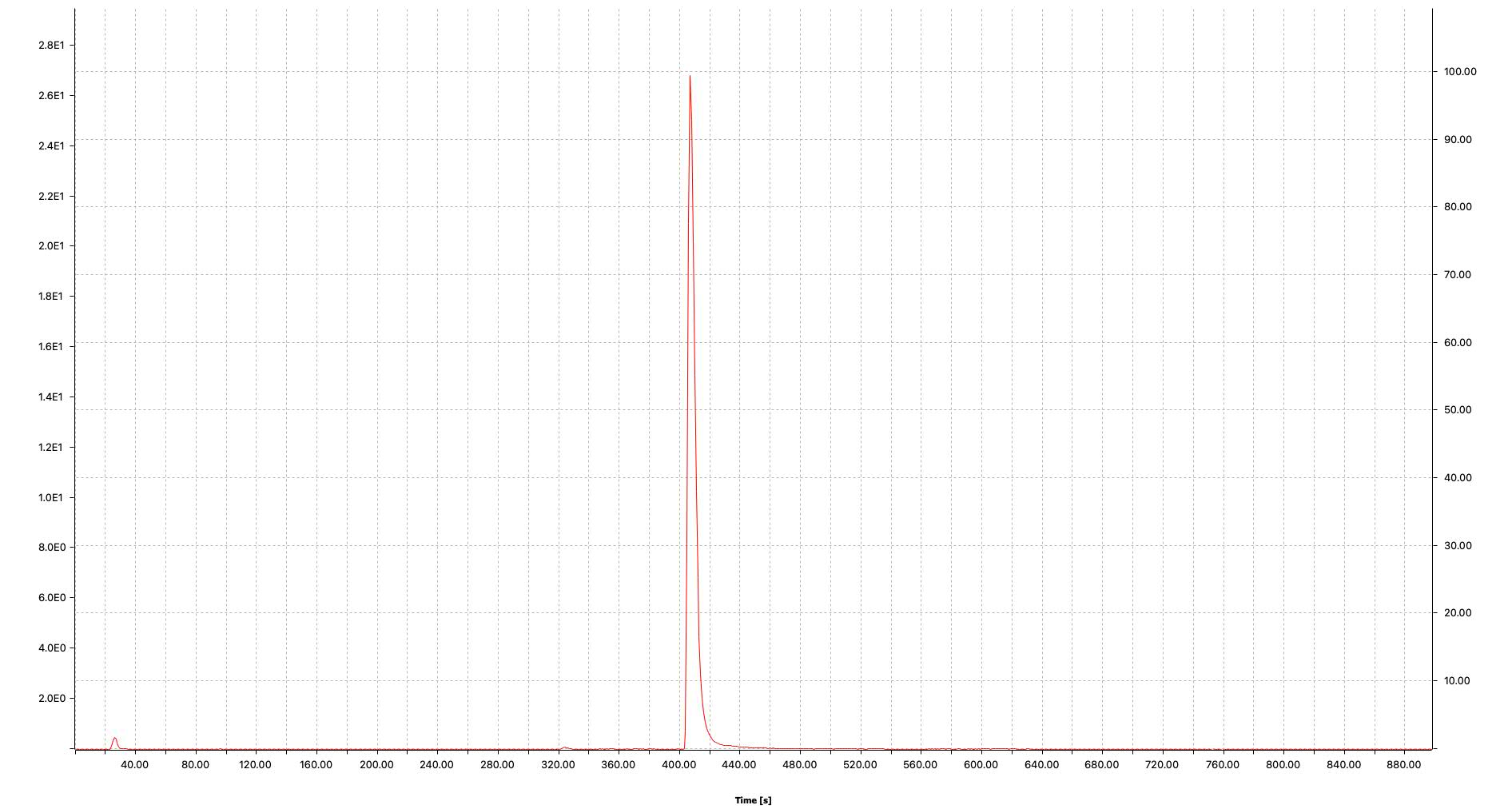


**Fig. S50: HPLC-UV chromatogram of RO0620809**


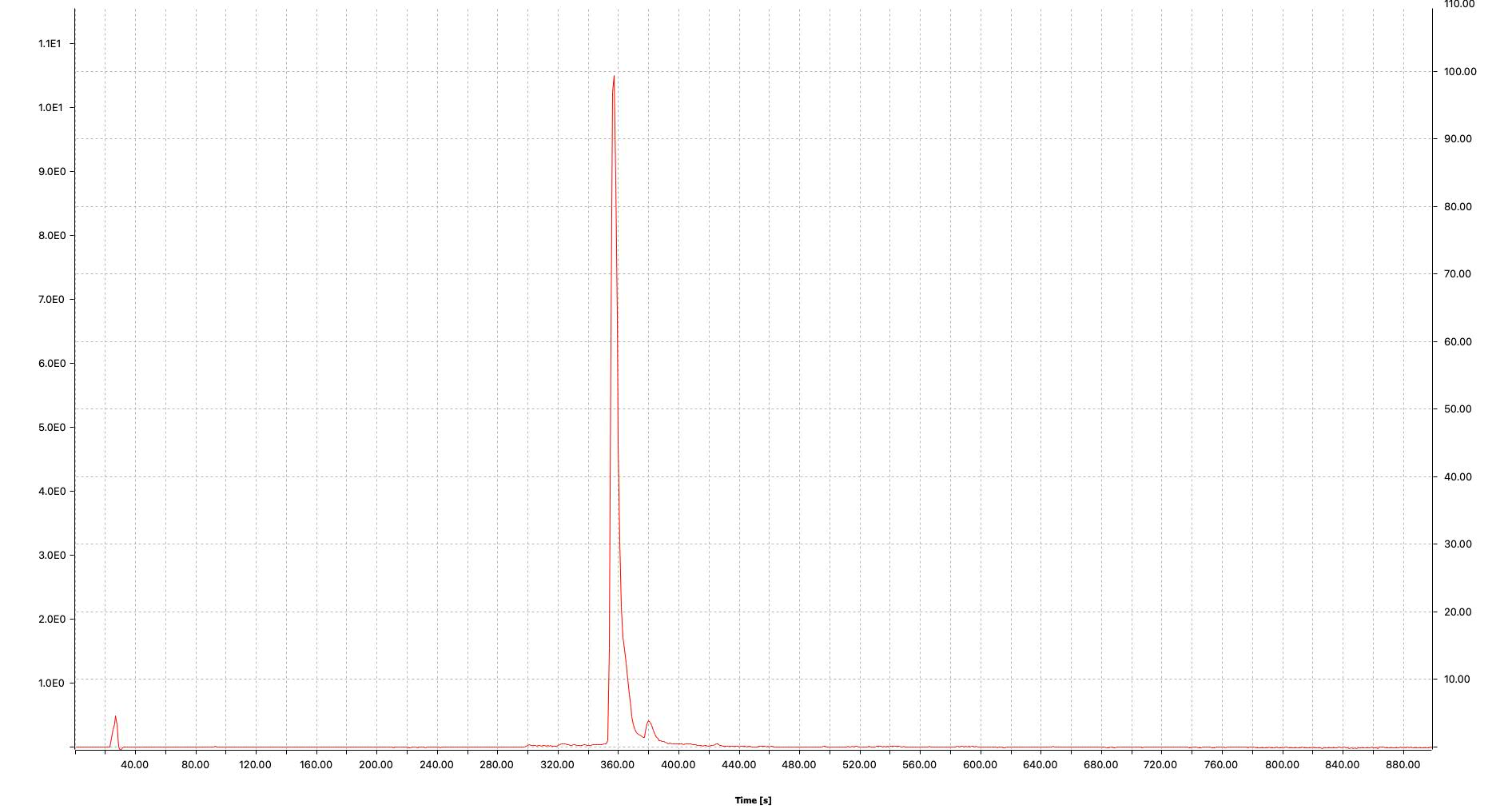


**Fig. S51: HPLC-UV chromatogram of RO0405919**


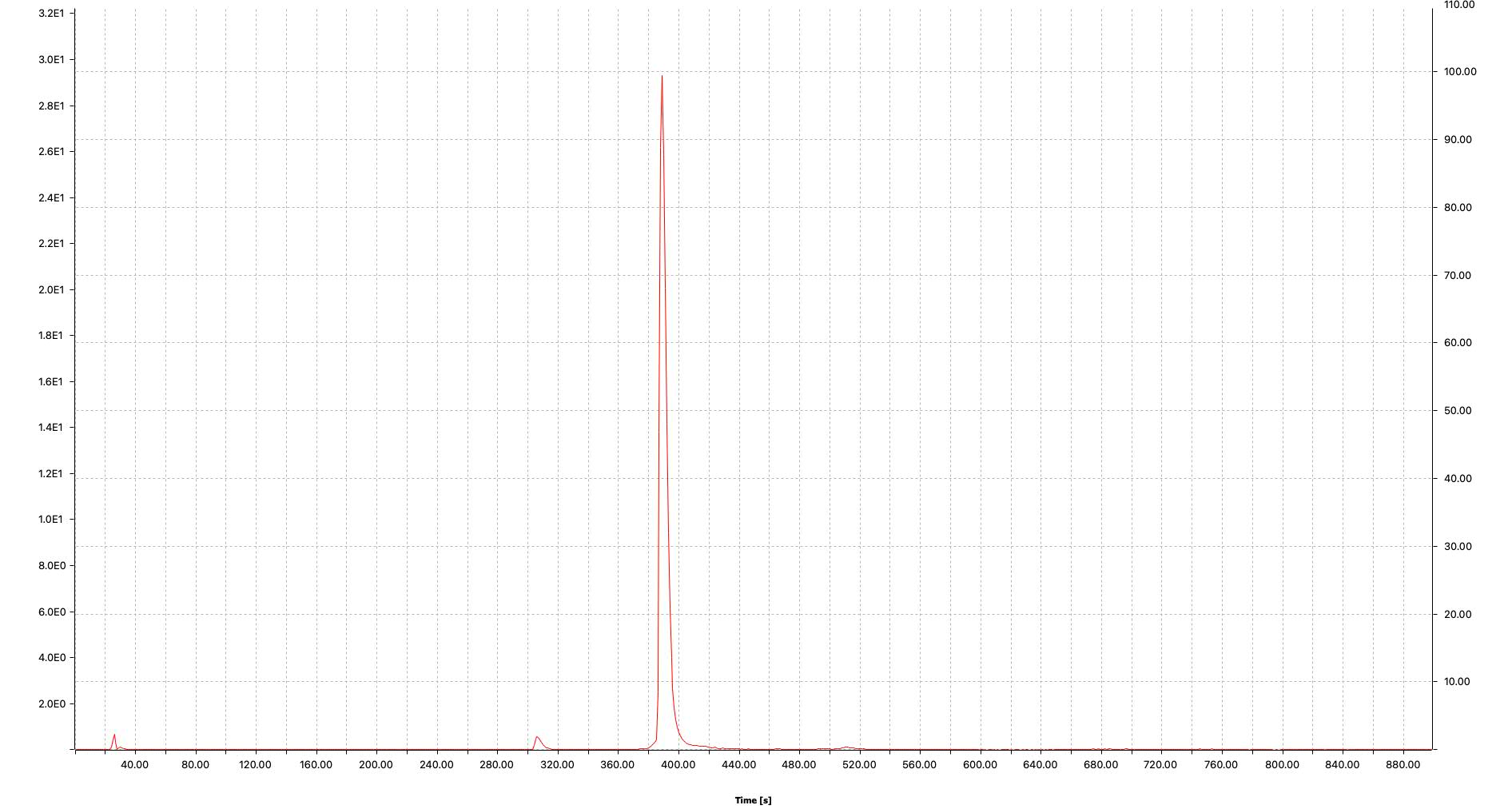


**Fig. S52: HPLC-UV chromatogram of RO0614977**


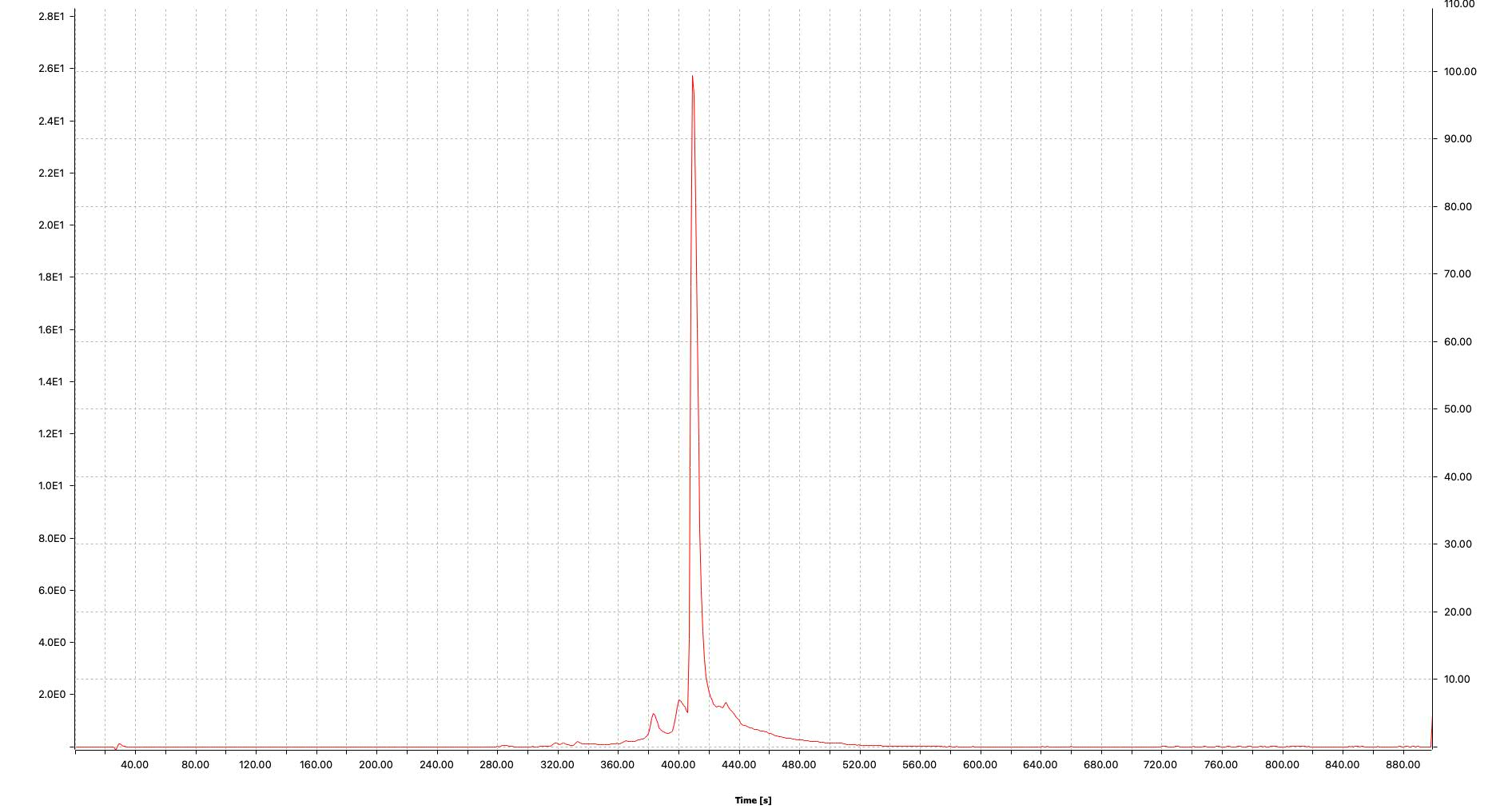


**Fig. S53: HPLC-UV chromatogram of RO0403335**


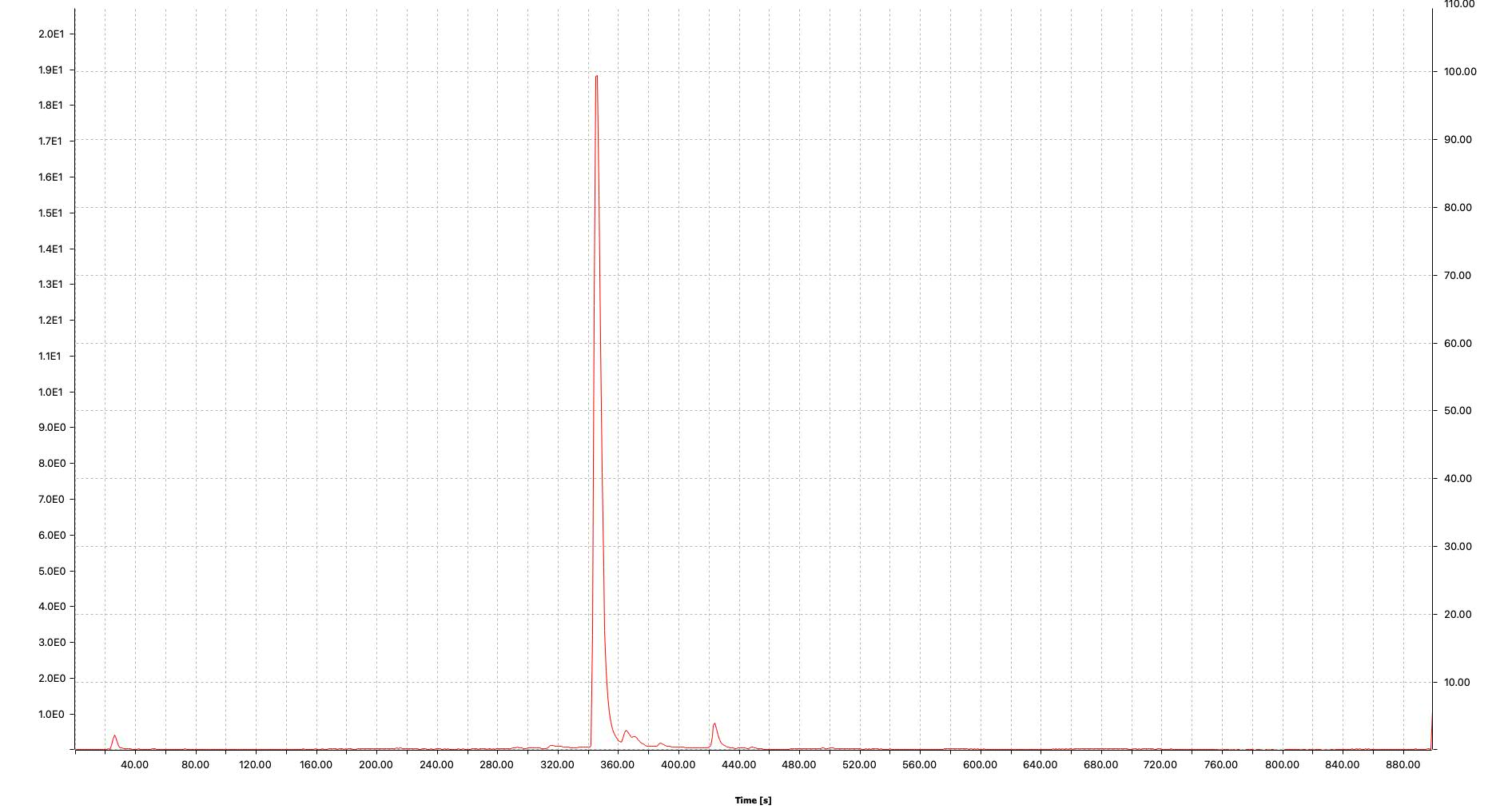


**Fig. S54: HPLC-UV chromatogram of RO0623430**

# References

[1] S. Kuhaudomlarp, E. Gillon, A. Varrot, A. Imberty, in *Lectin Purification and Analysis: Methods and Protocols* (Ed.: J. Hirabayashi), Springer US, New York, NY, **2020**, pp. 257–266.

[2] I. Joachim, S. Rikker, D. Hauck, D. Ponader, S. Boden, R. Sommer, L. Hartmann, A. Titz, *Org Biomol Chem* **2016**, *14*, 7933–7948.

[3] N. Fornstedt, J. Porath, *FEBS Lett* **1975**, *57*, 187–191.

[4] G. Beshr, A. Sikandar, E.-M. Jemiller, N. Klymiuk, D. Hauck, S. Wagner, E. Wolf, J. Koehnke, A. Titz, *Journal of Biological Chemistry* **2017**, *292*, 19935–19951.

[5] S. Kuhaudomlarp, E. Siebs, E. Shanina, J. Topin, I. Joachim, P. da Silva Figueiredo Celestino Gomes, A. Varrot, D. Rognan, C. Rademacher, A. Imberty, A. Titz, *Angewandte Chemie - International Edition* **2021**, *60*, 8104–8114.

[6] W. Kabsch, *Acta Crystallogr D Biol Crystallogr* **2010**, *66*, 125–132.

[7] P. Legrand, *Git hub repository https://github.com/legrandp/xdsme* **2017**, DOI 10.5281/zenodo.837885.

[8] J. Agirre, M. Atanasova, H. Bagdonas, C. B. Ballard, A. Baslé, J. Beilsten-Edmands, R. J. Borges, D. G. Brown, J. J. Burgos-Mármol, J. M. Berrisford, P. S. Bond, I. Caballero, L. Catapano, G. Chojnowski, A. G. Cook, K. D. Cowtan, T. I. Croll, J. Debreczeni, N. E. Devenish, E. J. Dodson, T. R. Drevon, P. Emsley, G. Evans, P. R. Evans, M. Fando, J. Foadi, L. Fuentes-Montero, E. F. Garman, M. Gerstel, R. J. Gildea, K. Hatti, M. L. Hekkelman, P. Heuser, S. W. Hoh, M. A. Hough, H. T. Jenkins, E. Jiménez, R. P. Joosten, R. M. Keegan, N. Keep, E. B. Krissinel, P. Kolenko, O. Kovalevskiy, V. S. Lamzin, D. M. Lawson, A. A. Lebedev, A. G. W. Leslie, B. Lohkamp, F. Long, M. Malý, A. J. McCoy, S. J. McNicholas, A. Medina, C. Millán, J. W. Murray, G. N. Murshudov, R. A. Nicholls, M. E. M. Noble, R. Oeffner, N. S. Pannu, J. M. Parkhurst, N. Pearce, J. Pereira, A. Perrakis, H. R. Powell, R. J. Read, D. J. Rigden, W. Rochira, M. Sammito, F. S. Rodríguez, G. M. Sheldrick, K. L. Shelley, F. Simkovic, A. J. Simpkin, P. Skubak, E. Sobolev, R. A. Steiner, K. Stevenson, I. Tews, J. M. H. Thomas, A. Thorn, J. T. Valls, V. Uski, I. Usón, A. Vagin, S. Velankar, M. Vollmar, H. Walden, D. Waterman, K. S. Wilson, M. D. Winn, G. Winter, M. Wojdyr, K. Yamashita, *Acta Crystallogr D Struct Biol* **2023**, *79*, 449–461.

[9] A. J. McCoy, in *Acta Crystallogr D Biol Crystallogr*, **2007**, pp. 32–41.

[10] G. Langer, S. X. Cohen, V. S. Lamzin, A. Perrakis, *Nat Protoc* **2008**, *3*, 1171–1179.

[11] K. Cowtan, *Acta Crystallogr D Biol Crystallogr* **2006**, *62*, 1002–1011.

[12] G. N. Murshudov, P. Skubák, A. A. Lebedev, N. S. Pannu, R. A. Steiner, R. A. Nicholls, M. D. Winn, F. Long, A. A. Vagin, *Acta Crystallogr D Biol Crystallogr* **2011**, *67*, 355–367.

[13] P. Emsley, B. Lohkamp, W. G. Scott, K. Cowtan, *Acta Crystallogr D Biol Crystallogr* **2010**, *66*, 486–501.

[14] F. Long, R. A. Nicholls, P. Emsley, S. Gražulis, A. Merkys, A. Vaitkus, G. N. Murshudov, *Acta Crystallogr D Struct Biol* **2017**, *73*, 112–122.

[15] P. Wenig, J. Odermatt, *BMC Bioinform.* **2010**, *11*.
